# Trapping Phyllophaga spp. (Coleoptera: Scarabaeidae: Melolonthinae) in the United States and Canada using sex attractants.

**DOI:** 10.1673/031.006.3901

**Published:** 2006-11-15

**Authors:** Paul S. Robbins, Steven R. Alm, Charles. D. Armstrong, Anne L. Averill, Thomas C. Baker, Robert J. Bauernfiend, Frederick P. Baxendale, S. Kris Braman, Rick L. Brandenburg, Daniel B. Cash, Gary J. Couch, Richard S. Cowles, Robert L. Crocker, Zandra D. DeLamar, Timothy G. Dittl, Sheila M. Fitzpatrick, Kathy L. Flanders, Tom Forgatsch, Timothy J. Gibb, Bruce D. Gill, Daniel O. Gilrein, Clyde S. Gorsuch, Abner M. Hammond, Patricia D. Hastings, David W. Held, Paul R. Heller, Rose T. Hiskes, James L. Holliman, William G. Hudson, Michael G. Klein, Vera L. Krischik, David J. Lee, Charles E. Linn, Nancy J. Luce, Kenna E. MacKenzie, Catherine M. Mannion, Sridhar Polavarapu, Daniel A. Potter, Wendell L. Roelofs, Brian M. Royals, Glenn A. Salsbury, Nathan M. Schiff, David J. Shetlar, Margaret Skinner, Beverly L. Sparks, Jessica A. Sutschek, Timothy P. Sutschek, Stanley R. Swier, Martha M. Sylvia, Neil J. Vickers, Patricia J. Vittum, Richard Weidman, Donald C. Weber, R. Chris Williamson, Michael G Villani

**Affiliations:** ^1^ Cornell Univ., New York State Agric. Experiment Station, Geneva, NY psr1@cornell.edu, cel1@cornell.edu, wlr1@cornell.edu; ^2^ Univ. of Rhode Island, Kingston, RI stevealm@uri.edu; ^3^ Univ. of Maine, Orono, ME charlesa@umext.maine.edu; ^4^ Univ. of Massachusetts, Amherst MA aaverill@ent.umass.edu, nluce@psis.umass.edu, pvittum@ent.umass.edu; ^5^ Pennsylvania State Univ., University Park PA tcb10@psu.edu, prh@psu.edu; ^6^ Kansas State Univ., Manhattan, KS rbauernf@oz.oznet.ksu.edu; ^7^ Univ. of Nebraska, Lincoln NE fbaxendale1@unl.edu; ^8^ Georgia Experiment Station, Griffin, GA kbraman@griffin.uga.edu; ^9^ North Carolina State Univ., Raleigh, NC rick_brandenburg@ncsu.edu, brian_royals@ncsu.edu; ^10^ Franklinville, NY dbcash@cnyti.com; ^11^ Cornell Univ. Cooperative Extension, Middletown, NY gjc15@cornell.edu; ^12^ Connecticut Agricultural Experiment Station, Windsor, CT richard.cowles@po.state.ct.us,; ^13^ Texas Department of Agriculture, Austin, TX robert.crocker@agr.state.tx.us; ^14^ Auburn Univ., Auburn, AL zd289coupe@yahoo.com, flandkl@auburn.edu; ^15^ Ocean Spray Cranberries, Babcock, WI tdittl@oceanspray.com; ^16^ Agriculture & Agri-Food Canada, Agassiz, British Columbia, Canada fitzpatricks@agr.gc.ca; ^17^ Bandon, OR osonegro@peoplepc.com; ^18^ Purdue Univ., West Lafayette, IN gibb@purdue.edu; ^19^ Center for Plant Quarantine Pests, Ottawa, Canada gillbd@inspection.gc.ca; ^20^ Cornell Univ. Cooperative Extension, Riverhead, NY dog1@cornell.edu; ^21^ Clemson Univ., Clemson, SC csgorsuch@att.net; ^22^ Louisiana State Univ., Baton Rouge, LA ahammond@agcenter.lsu.edu; ^23^ Rutgers Univ. Cooperative Extension, New Brunswick, NJ hastings@aesop.rutgers.edu, weidman@rcre.rutgers.edu; ^24^ Mississippi State Univ, Biloxi, MS dwh56@ra.msstate.edu; ^25^ Alabama Agricultural Experiment Station, Marion Junction, AL jhollima@acesag.auburn.edu; ^26^ Univ. of Georgia, Tifton, GA wghudson@uga.edu; ^27^ Ohio State Univ., Wooster, OH klein.10@osu.edu; ^28^ Univ. of Minnesota, St. Paul, MN krisc001@tc.umn.edu; ^29^ New York State Tree Nursery, Saratoga Springs, NY djlee@gw.dec.state.ny.us; ^30^ Agriculture & Agri-Food Canada, Kentville, Nova Scotia, Canada mackenziek@agr.gc.ca; ^31^ Univ. of Florida, Homestead, FL cmannion@mail.ifas.ufl.edu; ^32^ Rutgers Univ., Blueberry and Cranberry Research Center, Chatsworth, NJ; ^33^ Univ. of Kentucky, Lexington, KY dapotter@uky.edu; ^34^ Kansas Department of Agriculture, Greensburg, KS gsalsbury@sbcglobal.net; ^35^ USDA Forest Service, Stoneville, MS nschiff@asrr.arsusda.gov; ^36^ Ohio State Univ., Columbus, OH shetlar.1@osu.edu; ^37^ Univ. of Vermont, Burlington, VT margaret.skinner@uvm.edu; ^38^ Univ. of Georgia, Athens, GA bsparks@uga.edu; ^39^ Tarpon Springs, FL jessie_sutschek@yahoo.com; ^40^ Univ. of New Hampshire, Durham, NH stan.swier@unh.edu; ^41^ Univ. of Massachusetts Cranberry Experiment Station, Wareham, MN martys@umext.umass.edu; ^42^ Univ. of Utah, Salt Lake City, UT vickers@biology.utah.edu; ^43^ USDA-ARS, Beltsville, MD weberd@ba.ars.usda.gov; ^44^ Univ. of Wisconsin, Madison, WI rcwillie@entomology.wisc.edu; ^†^Deceased - Sridhar Polavarapu and Michael Villani are greatly missed by family, friends, and colleagues.

## Abstract

The sex pheromone of the scarab beetle, Phyllophaga anxia, is a blend of the methyl esters of two amino acids, L-valine and L-isoleucine. A field trapping study was conducted, deploying different blends of the two compounds at 59 locations in the United States and Canada. More than 57,000 males of 61 Phyllophaga species (Coleoptera: Scarabaeidae: Melolonthinae) were captured and identified. Three major findings included: (1) widespread use of the two compounds [of the 147 Phyllophaga (sensu stricto) species found in the United States and Canada, males of nearly 40% were captured]; (2) in most species intraspecific male response to the pheromone blends was stable between years and over geography; and (3) an unusual pheromone polymorphism was described from P. anxia. Populations at some locations were captured with L-valine methyl ester alone, whereas populations at other locations were captured with L-isoleucine methyl ester alone. At additional locations, the L-valine methyl ester-responding populations and the L-isoleucine methyl ester-responding populations were both present, producing a bimodal capture curve. In southeastern Massachusetts and in Rhode Island, in the United States, P. anxia males were captured with blends of L-valine methyl ester and L-isoleucine methyl ester.

## Resumen

La feromona sexual del escarabajo, Phyllophaga anxia, es una mezcla de los ésteres metílicos de dos aminoácidos, L-valina y L-isoleucina. Se condujo un estudio de campo usando diferentes mezclas de los dos componentes en 59 sitios de Estados Unidos y Canada. Más de 57,000 machos de 61 especies de Phyllophaga fueron capturados e identificados. Tres de los resultados más importantes incluyen: (1) el extenso uso de los dos componentes [de las 147 especies de Phyllophaga (sensu stricto), en Estados Unidos y Canada, fueron capturados machos de cerca del 40% de ellas.]; (2) para la mayoría de las especies, la respuesta intraespecífica de los machos a las combinaciones de los dos aminoácidos fue consistente entre años diferentes, y en todos los sitios geográficos; y (3) un inusual polymorfismo de la feromona fue descrito para P. anxia. Poblaciones de algunos sitios fueron atrapados sólo con valina, mientras que poblaciones de otros sitios fueron atrapados sólo con isoleucina. También se encontraron sitios donde las poblaciones responden a ambos componentes, valina e isoleucina, produciendo una curva de captura bimodal. En el sureste del estado de Massachusetts y en Rhode Island, en Estados Unidos, machos de P. anxia fueron atrapados en trampas con mezclas de valina e isoleucina.

## Introduction

The scarab beetle genus Phyllophaga (sensu lato) is one of the largest genera of animals in the United States (Woodruff and Beck 1989), encompassing 203 described species in 8 subgenera, including 147 species (and 8 subspecies) in the subgenus Phyllophaga (sensu stricto), 39 species in Listrochelus, 7 species in Phytalus, 3 species in Cnemarachis (all non-native and confined to south Florida), 3 species in Eugastra, 2 species in Tostegoptera, 1 species in Chlaenobia, and 1 species in Triodonyx (Evans 2003, Smith and Evans 2005). Their striking genitalic morphology, first described in the late 19^th^ century (Smith 1888), continues to be the most important taxonomic character used to separate species in this group (Luginbill and Painter 1953). See Woodruff and Beck (1989) and Woodruff and Sanderson (2004) to view excellent scanning electron microscopy images of Phyllophaga genitalia.

The economic importance of this genus relates principally to the root feeding habits of the larvae, commonly called white grubs. Larvae of various species of Phyllophaga have been recorded feeding on crops that include, but are not limited to, nursery stock (Andre 1937), corn (Bessin 1999), commercial turfgrass (Brandenburg and Villani 1995; Vittum et al. 1999), cranberries (Dunn and Averill 1996; Eck 1990; Franklin 1950), sugarcane (Gordon and Anderson 1981), sweet potato (Hammond et al. 1997), and pasture (Luginbill and Painter 1953). True to the Greek origin of their generic name (Phyllo-leaf + phaga-eat), adult Phyllophaga in very large flights have been known to defoliate stands of trees. Although adults of some Phyllophaga species are apparently host specific, most are polyphagous (Luginbill and Painter 1953).

P. anxia (LeConte) is the most widely distributed Phyllophaga species in North America (Luginbill and Painter 1953; Woodruff and Beck 1989). Two genitalic morphs are described in this species, the northern form and the southern form (Luginbill and Painter 1953; Woodruff and Beck 1989). The first sex pheromone described from the genus Phyllophaga was identified from virgin P. anxia adults dug in mid-April from a cranberry bog in Carver, Massachusetts, before their May flight. The female-produced sex pheromone was determined to be a 75/25 blend of L-valine methyl ester and L-isoleucine methyl ester (Zhang et al. 1997). L-isoleucine methyl ester was first elucidated from Holotrichia parallela (Leal et al. 1992), an Asian melolonthine species. Holotrichia is regarded by some as being inseparable taxonomically from the Nearctic Phyllophaga (Saylor 1937; Saylor 1939).

L-valine methyl ester and L-isoleucine methyl ester are unusual pheromone compounds in that their precursors are the essential amino acids, L-valine and L-isoleucine. These essential amino acids are available only via sequestration from food plants fed on by the larvae, or perhaps from endo-symbionts. Our future investigations will hopefully determine the source of these amino acids. For the most recent overview of beetle semiochemicals see Francke and Dettner (2005).

Since P. anxia is a common species throughout the Northeast, the pheromone was deployed in the field near Geneva, New York, in 1996. P. anxia males were captured with this blend, as expected. However, another species of Phyllophaga, P. futilis, was also captured in the traps in much smaller numbers. Our interest was piqued by the P. futilis catches because sex pheromones are generally regarded as species-specific mate recognition signals, although studies have demonstrated varying degrees of pheromone specificity between closely related species (Roelofs 1978), as well as pheromone polymorphism in geographically separated conspecifics such as Agrotis segetum (Wu et al. 1999), Ostrinia nubilalis (Glover et al. 1991; Klun and Cooperators 1975), and Hemileuca eglanterina (McElfresh and Millar 2001). Since P. anxia is a common species throughout most of North America, this finding presented an opportunity to examine the response specificity of different populations of P. anxia, as well as responses of other Phyllophaga species, over a large geographic region.

## Materials and Methods

**Figure 1:**
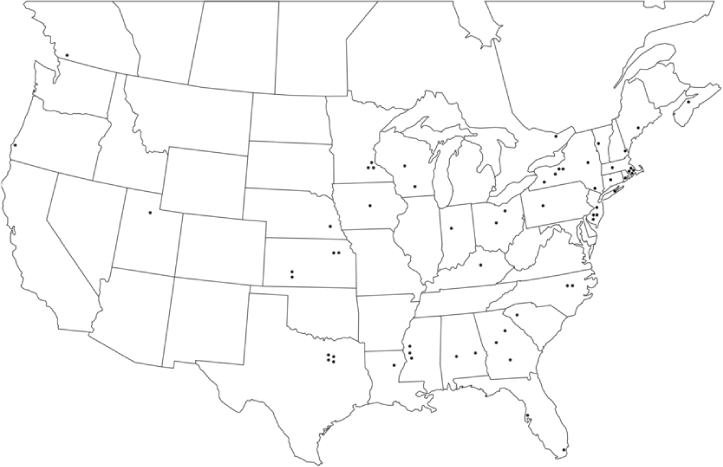
Phyllophaga spp. trapping sites, 1996–2001

Vane traps baited with various blends of L-valine methyl ester and L-isoleucine methyl ester were deployed at 59 different locations in the U.S. and Canada during the years 1996–2001 (Figure 1). At each of these locations, traps were maintained for one to four seasons. Tables 1a and 1b list the trap locations, years during which traps were deployed, and a brief note about the habitat. The trapping sites in Carver, Lakeville, and Plympton, Massachusetts; Chatsworth, New Jersey; Babcock, Wisconsin; Lincolnville Center, Maine; Aggasiz, British Columbia; and Bandon, Oregon, were chosen because the traps could be located adjacent to cranberry acreage. Researchers involved in studies of Phyllophaga infesting turf, pasture, nursery, or other commodities maintained some trapping sites. Other sites were selected because it was likely that they might harbor different Phyllophaga species from those in other geographic areas and where the lures had never been tested.

**Table 1a:**
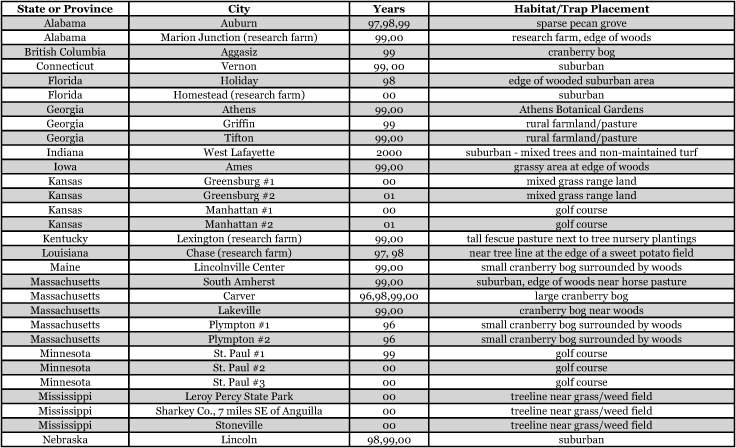


**Table 1b:**
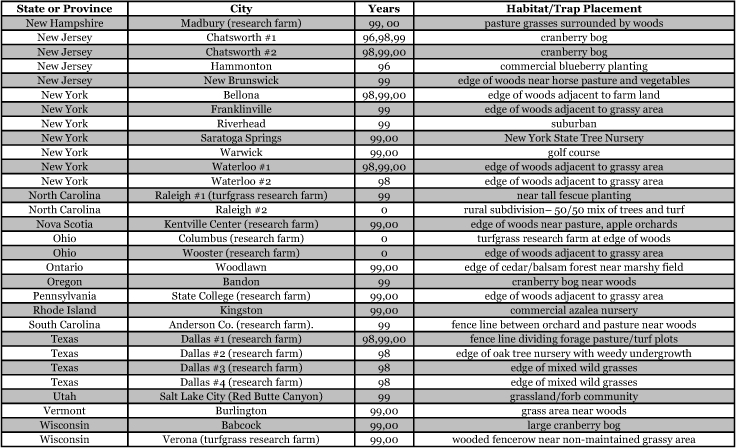


When blends are referred to in this study, it is in the ratio of L-valine methyl ester/L-isoleucine methyl ester. In 1996, five blends were deployed, including 100% L-valine methyl ester, 65/35, 50/50, 35/65, and 100 % L-isoleucine methyl ester. In 1997 and 1998, 95/5 and 5/95 blends were added to the array. In 1999, 2000, and 2001, the eight blends tested included 100% L-valine methyl ester, 90/10, 80/20, 60/40, 40/60, 20/80, 10/90, and 100% L-isoleucine methyl ester. In 1996, the lures were produced in our own laboratory by loading 5 mm rubber stopper septa (Thomas Scientific, www.thomassci.com/index.jsp) with 3 mg each of various blends using hexane as the solvent. From 1997 to 2001, Dr. A.C. Oehlschlager of ChemTica Internacional S.A. (San Jose, Costa Rica, www.chemtica.com) generously supplied the rubber septa lures for the tests. During that time, the lures were loaded with 4 mg of the various blends. At each location a control trap with a blank septum also was deployed.

**Figure 2:**
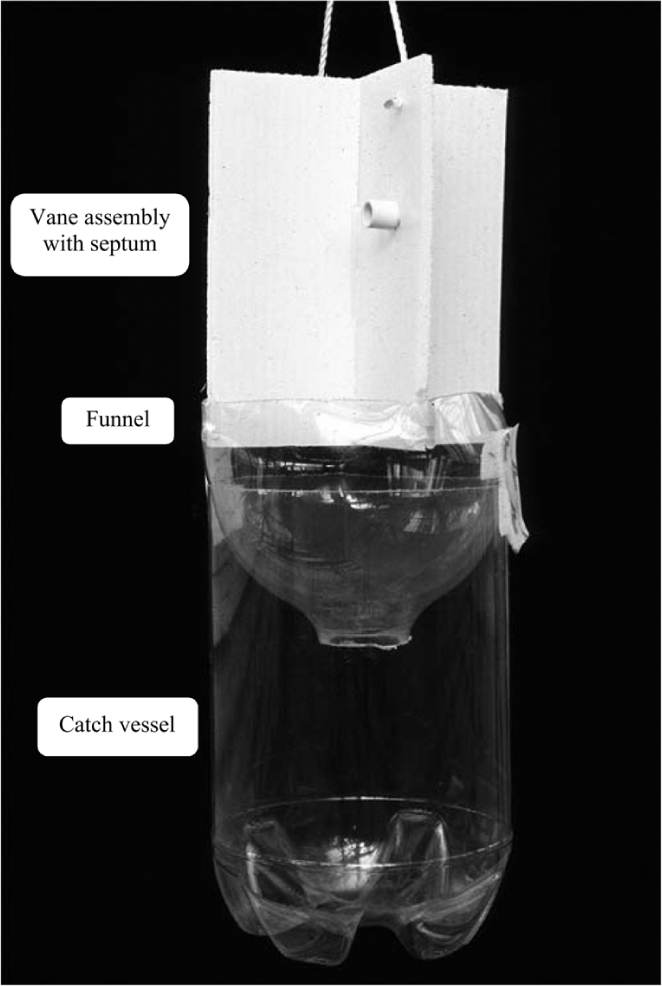
Cross vane trap constructed from 3 liter beverage container. Pheromones were applied to the rubber septum.

In 1996 and 1997, the traps used in the study were either Trécé Japanese beetle vane traps (Trécé Incorporated, www.trece.com/) or Fuji Flavor Company vane traps (Fuji Flavor Company, www.fjf.co.jp/). Beginning in the 1998 season, vane traps were fabricated in the laboratory from three-liter soda bottles and 4 mm white corrugated plastic (Figure 2). When removed from the field during the winter, these traps lasted up to three field seasons.

Traps were set in the field 15–20 meters apart and at heights of 1–2 meters. The traps were checked and re-randomized one to three times each week. Captured beetles were bagged and frozen, or infrequently preserved in ethanol. Plastic bags or bottles marked with the catch date and blend were shipped at the completion of the trapping period to Geneva, NY, for identification.

Phyllophaga species identifications were assigned using a number of published sources (Luginbill and Painter 1953;Ratcliffe 1991;Riley 1988, Saylor 1939; Saylor 1940; Woodruff and Beck 1989), comparisons with Phyllophaga species in the Cornell University insect collection, and consultations with and verifications by E. Richard Hoebeke (Cornell University, Ithaca, NY), Dr. Paul Lago (University of Mississippi, University, MS), Edward C. Riley (Texas A & M University, College Station, TX), and William B. Warner (Farnam Companies, Inc., Phoenix, AZ). In 1998, Dr. Robert Crocker (then at Texas A & M University, Dallas, TX) did identifications of the Texas catch and sent the results to Geneva NY. In 2000 and 2001, Dr. Robert Bauernfiend (Kansas State University, Manhattan, KS) did the same for the Manhattan, Kansas catches. Whenever possible, a series of each species from the various locations was pinned for later vouchering in the Cornell University insect collection.

## Results and Discussion

### General observations

The following outline condenses the large number of figures and tables found in this publication into general groupings for the convenience of the reader.

**Table:**
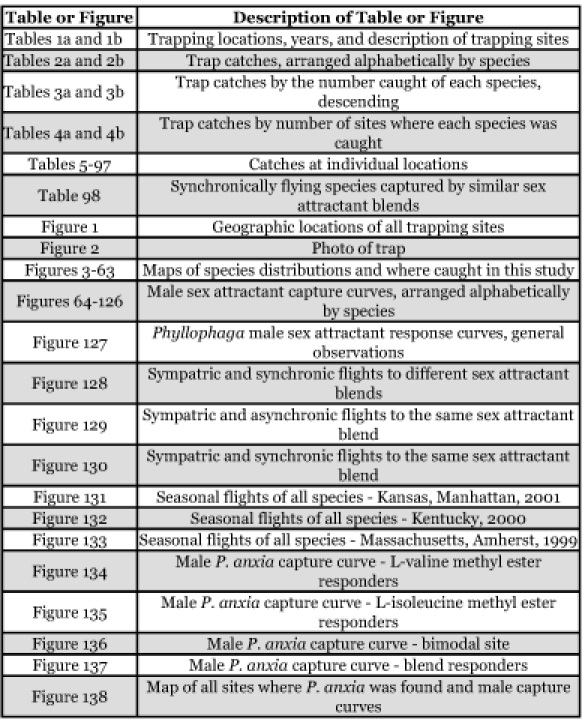


A total of 57,129 Phyllophaga individuals were examined and identified in the course of this study. The 215 captured females represented only 0.38% of the total catch, suggesting that the attractants do not function as aggregation pheromones in any of the species. A total of 145 males were counted from the control traps, amounting to only 0.25% of the total catch.

No Phyllophaga beetles were captured in Aggasiz, British Columbia; Bandon, Oregon; or Homestead, Florida. In British Columbia and Oregon, the traps were located at cranberry bogs. A light trap operated at the Oregon cranberry bog site also captured no Phyllophaga. Despite extensive sampling for root weevils in northwest commercial cranberry bogs, no white grubs have been reported (D. C. Weber, unpublished data). Moreover, when the Phyllophaga species distribution maps in Luginbill and Painter (1953) are examined, only four species of Phyllophaga are found listed from Oregon. Regarding the Homestead, Florida site, Woodruff (1961) indicates that very few of the Florida Phyllophaga species occur in Miami or in the Florida Keys. In the same publication, he points out that “The soil in the Miami area is extremely shallow and underlain by öolitic limestone and, in general, is not a good soil for white grubs.” Only 4 of the 42 species of Phyllophaga recorded from Florida have been collected in that area.

Tables 2a and 2b list alphabetically the Phyllophaga species captured, the number of males captured in each species, the number of discrete locations at which they were captured, and the total number of site-years for each species [site-years = (site A x number of years that species was captured at that site) + (site B x number of years that species was captured at that site) + .......]. Tables 3a and 3b are sorted by descending catch numbers, beginning with the Phyllophaga species caught in the greatest number. Tables 4a and 4b are sorted by site, listing in descending order the number of sites at which a particular species was recorded.

**Table 2a.**
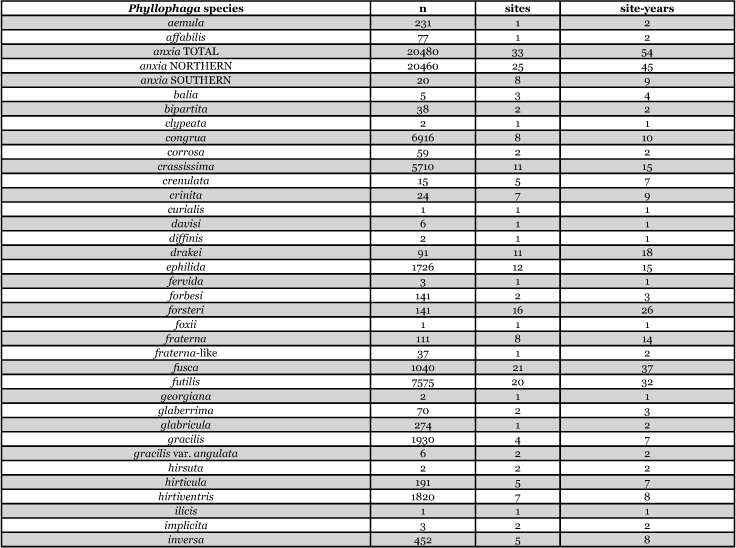
Male catches, sites, and sites-years sorted by species.

**Table 2b:**
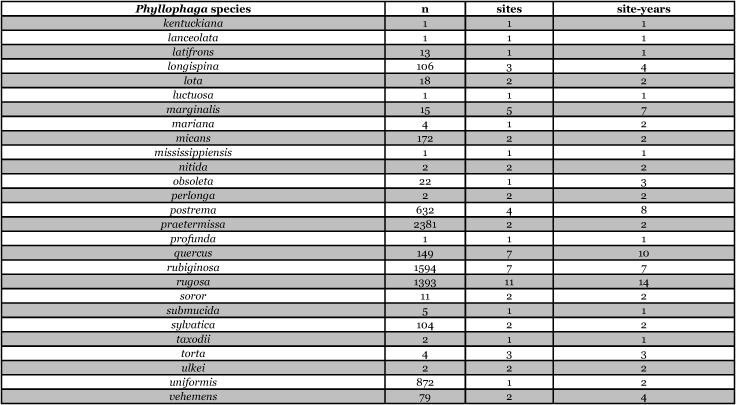
Male catches, sites, and sites-years sorted by species.

**Table 3a:**
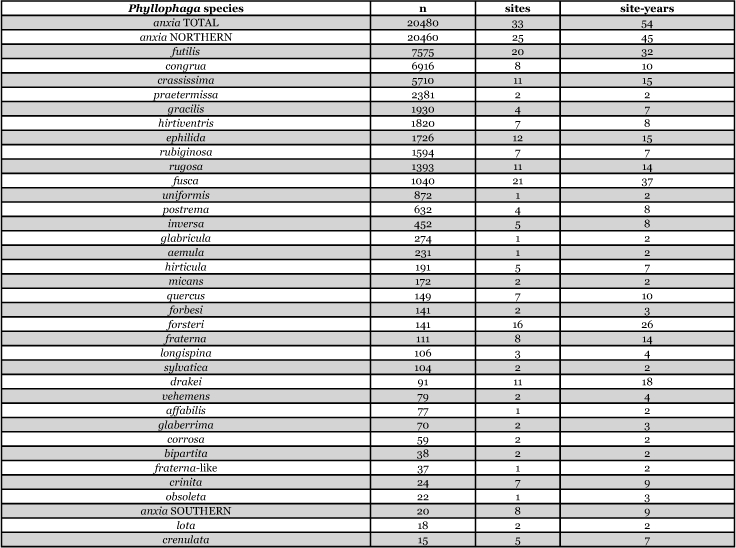
Male catches, sites, and sites-years sorted by n.

**Table 3b:**
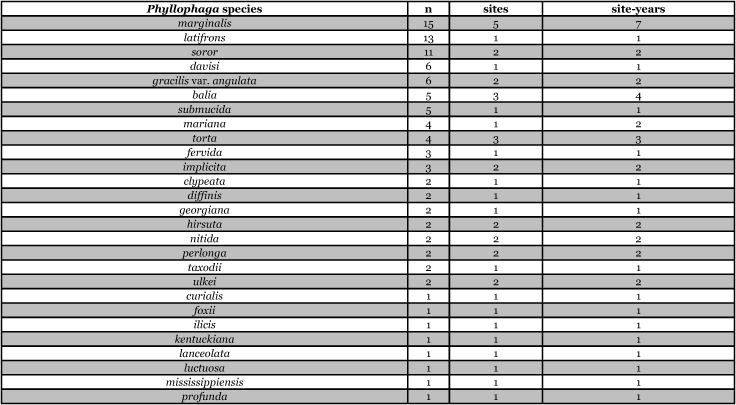
Male catches, sites, and sites-years sorted by n.

**Table 4a:**
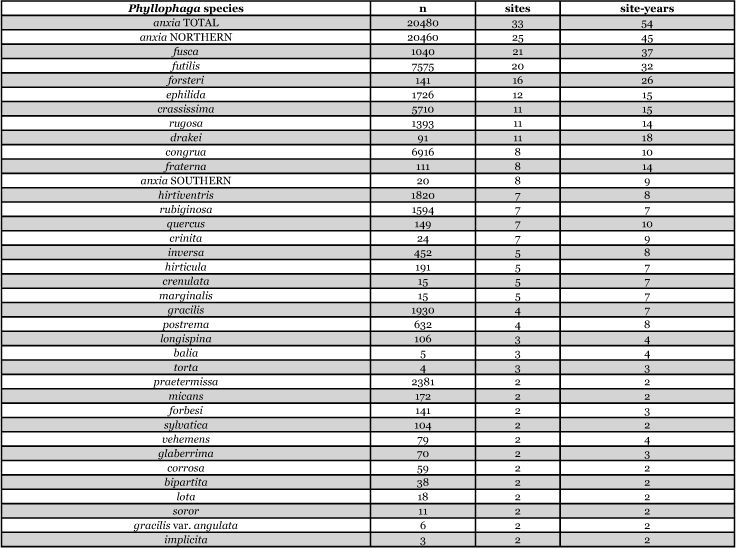
Male catches, sites, and sites-years sorted by sites.

**Table 4b:**
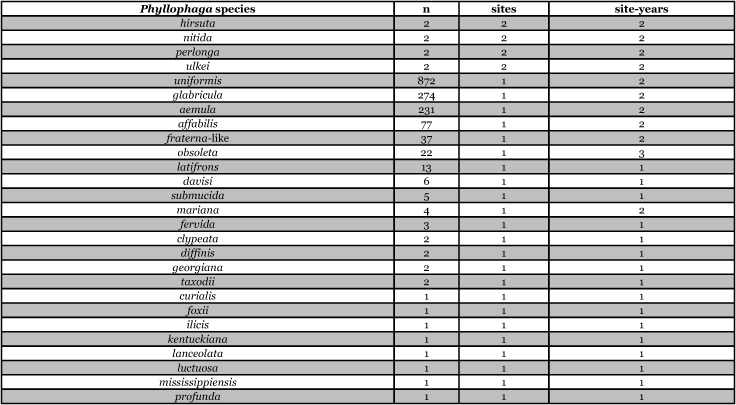
Male catches, sites, and sites-years sorted by sites.

Of all site-years when and where beetles were captured, only two (NJ Chatsworth #2, 1999 Table 59 and UT Salt Lake City, 1999, Table 91) recorded a single species of Phyllophaga during a flight season. The average number of species captured during a site-year was 4.15. Three sites (AL Auburn, 1998, Table 6; KS Manhattan #1, 2000, Table 27; and KS Manhattan #2, 2001, Table 28) recorded more than 10 species of Phyllophaga captured during the flight season.

The species that was taken in the greatest number (20,480), in the greatest number of sites (33), and in the greatest number of site-years (54) was P. anxia. This is not surprising given that, as was indicated previously, it is the most widespread species of Phyllophaga in North America. Luginbill and Painter (1953) list it as occurring in every state in the United States except Arizona, California, Florida, Nevada, West Virginia, and Wyoming. They also report P. anxia from all ten Canadian provinces. Woodruff and Beck (1989) have subsequently reported it from Florida. W. B. Warner (personal communication) has examined a specimen of P. anxia from California and has reports of P. anxia in Arizona.

Males of ten other Phyllophaga species were also captured in numbers >1000. These include P. futilis, P. congrua, P. crassissima, P. praetermissa, P. gracilis, P. hirtiventris, P. ephilida, P. rubiginosa, P. rugosa, and P. fusca. Males of 13 species were captured in numbers >100 but <1000 and males of 15 species were captured in numbers >10 but <100 (Tables 3a and 3b). Eighteen species of Phyllophaga were recorded at ≥5 locations and 8 species were recorded at ≥11 locations (Tables 4a and 4b).

### Geographical distributions and range extensions

Geographical distribution maps of Phyllophaga species captured during this study are found in Figures 3–63, arranged alphabetically by species. Shaded areas in these figures indicate the geographical ranges conveyed by Luginbill and Painter (1953) or Woodruff and Beck (1989) [P. (Phytalus) georgiana and P. (Phytalus) obsoleta only].

**Figure 3:**
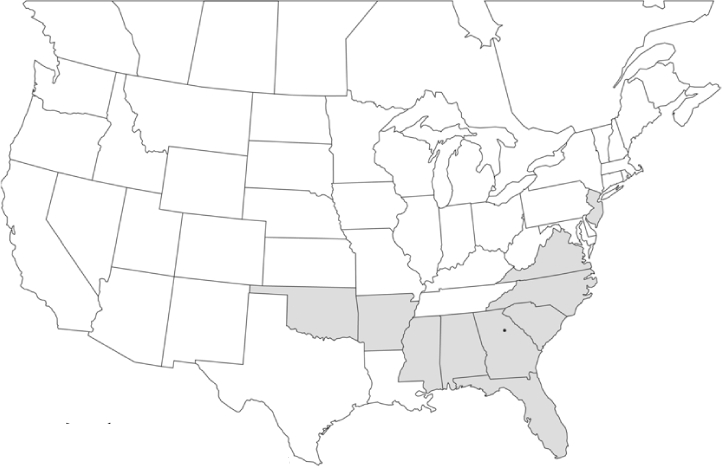
**P. aemula** • = catch sites, n = 231 beetles Shaded areas = distributions from Luginbill and Painter, 1953

Most captures of Phyllophaga species recorded during this study were found within the geographical species distributions reported by Luginbill and Painter (1953). There are, however, several range extensions to report. The following species were found in locations in addition to those reported by Luginbill and Painter: P. curialis (Figure 14), P. drakei (Figure 17), P. forbesi (Figure 20), P. foxii (Figure 22), P. futilis (Figure 25), P. gracilis (Figure 29), P. gracilis var. angulata (Figure 30), P. hirtiventris (Figure 33), P. longispina (Figure 40), P. lota (Figure 41), P. marginalis (Figure 43), P. (fraterna) mississippiensis (Figure 46), P. postrema (Figure 50), P. praetermissa (Figure 51), P. quercus (Figure 53), and P. taxodii (Figure 59). Riley (1988) had previously noted range extensions of P. forbesi, P. quercus, and P. taxodii into Louisiana.

**Figure 4:**
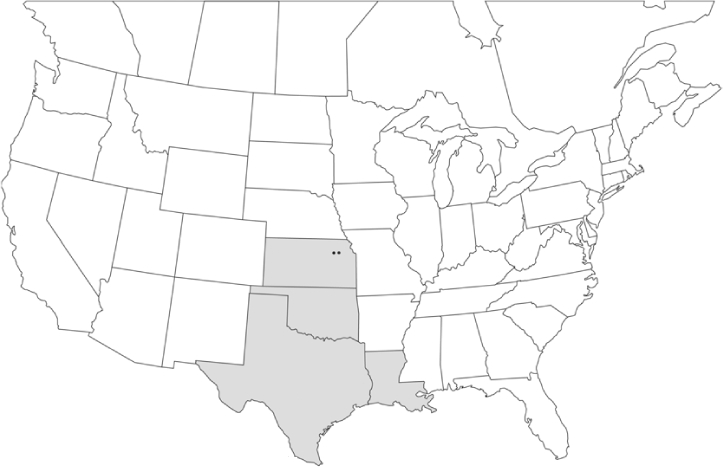
**P. affabilis** • = catch sites, n = 77 beetles Shaded areas = distributions from Luginbiil and Painter, 1953

**Figure 5:**
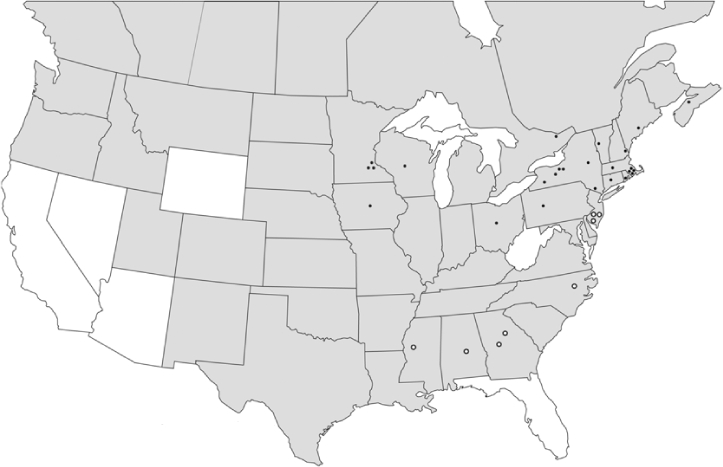
**P. anxia** • = catch siles-norlhem form, n = 20,640 beetles ○ = catch sites-southern form, n = 20 beetles Shaded areas = distributions from Luginbill and Painter, 1953

**Figure 6:**
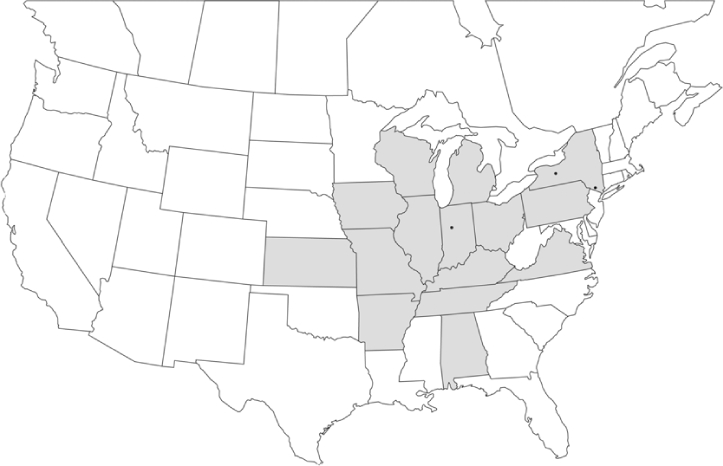
**P. balia** • = catch sites, n = 5 beetles Shaded areas = distributions from Luginbill and Painter, 1953

**Figure 7:**
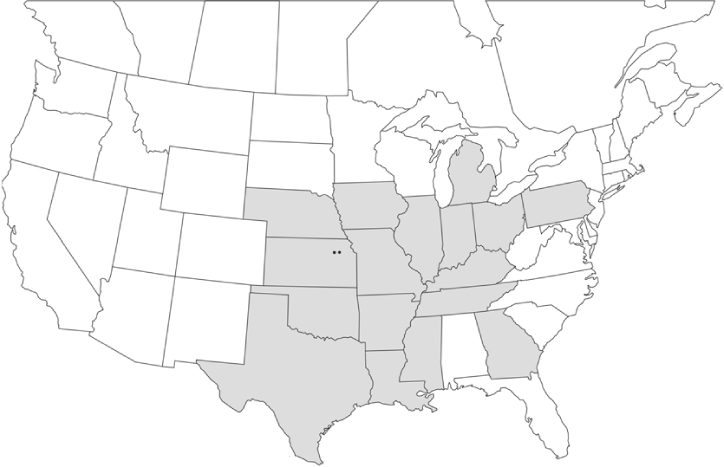
**P. bipartita** • = catch sites, n = 38 beetles Shaded areas = distributions from Luginbill and Painter, 1953

**Figure 8:**
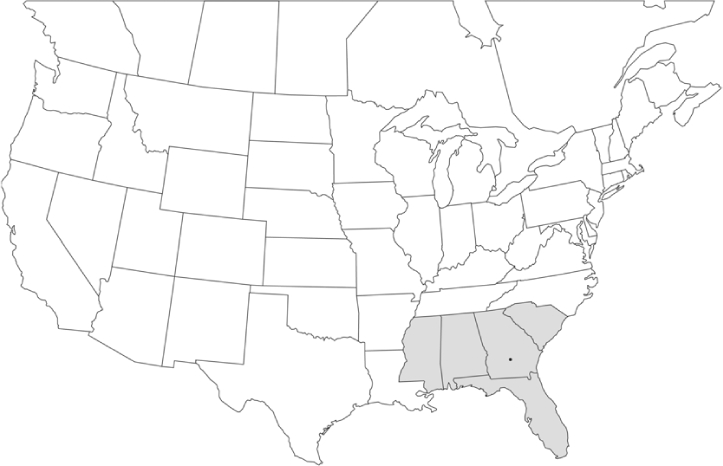
**P. clypeata** • = catch sites, n = 2 beetles Shaded areas = distributions from Luginbill and Painter, 1953

**Figure 9:**
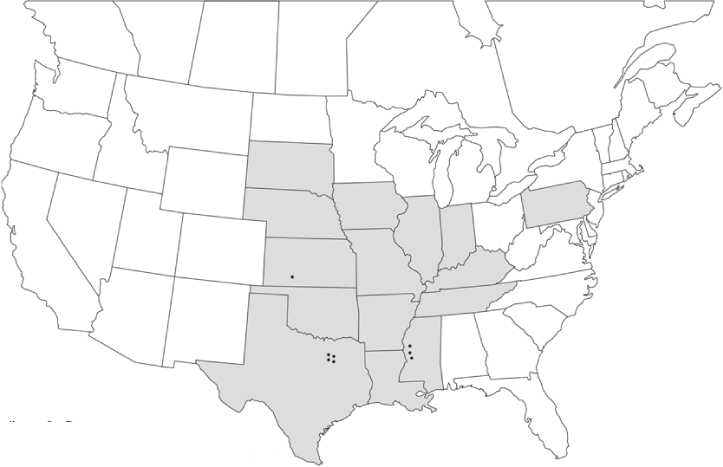
**P. congrua** •= catch sites, n = 6916 beetles Shaded areas = distributions from Luginbill and Painter, 1953

**Figure 10:**
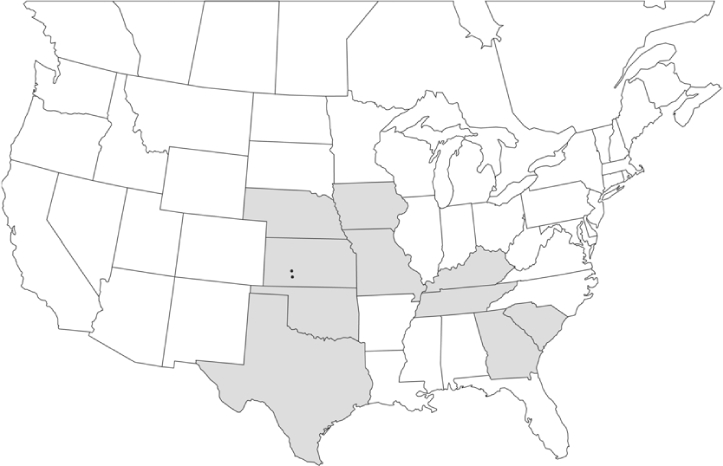
**P. corrosa** • = catch sites, n = 59 beetles Shaded areas = distributions from Luginbill and Painter, 1953

**Figure 11:**
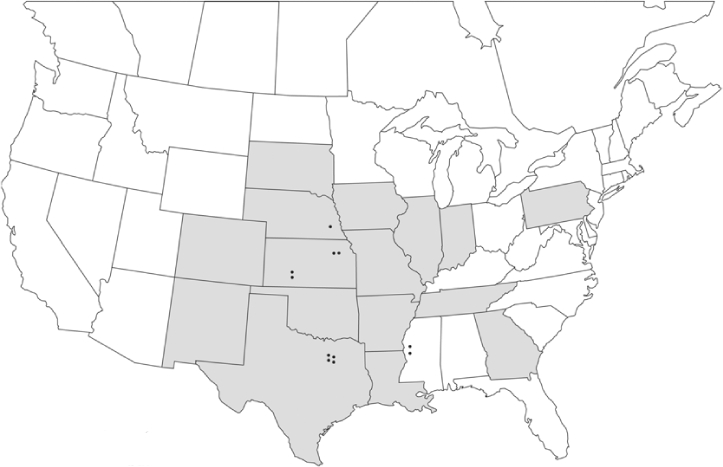
**P. crassissima** • = catch sites, n = 5710 beetles Shaded areas = distributions from Luginbill and Painter, 1953

**Figure 12:**
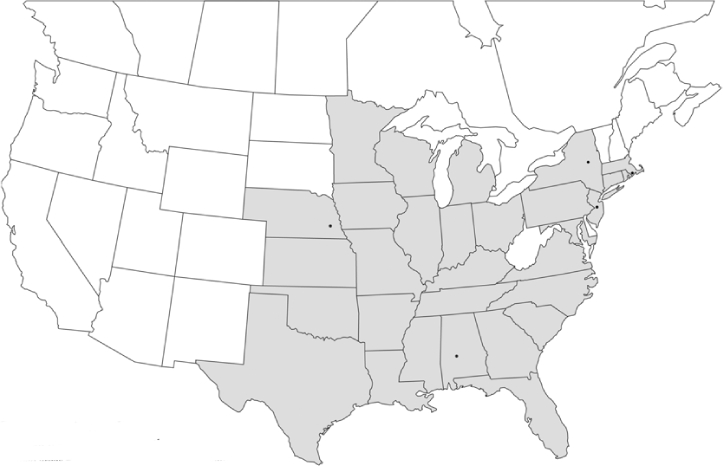
**P. crenulata** • = catch sites, n = 15 beetles Shaded areas = distributions from Luginbill and Painter, 1953

**Figure 13:**
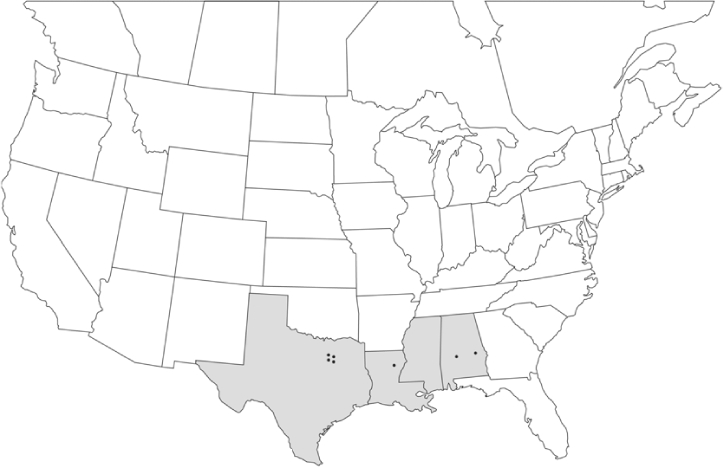
**P. crinita** • = catch sites, n = 24 beetles Shaded areas = distributions from Luginbill and Painter, 1953

**Figure 14:**
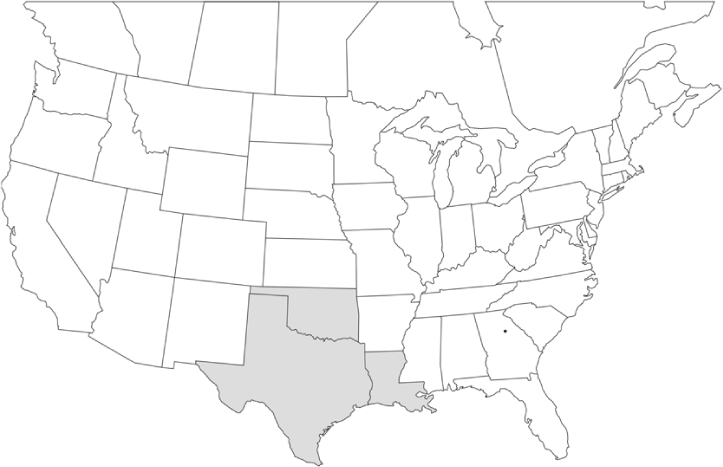
**P. curialis** • = catch sites, n = 1 beetle Shaded areas = distributions from Luginbill and Painter, 1953

**Figure 15:**
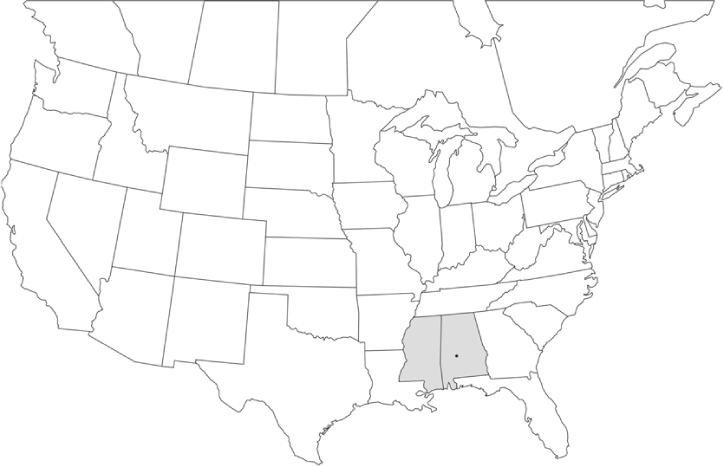
**P. davisi** • = catch sites, n = 6 beetles Shaded areas = distributions from Luginbill and Painter, 1953

**Figure 16:**
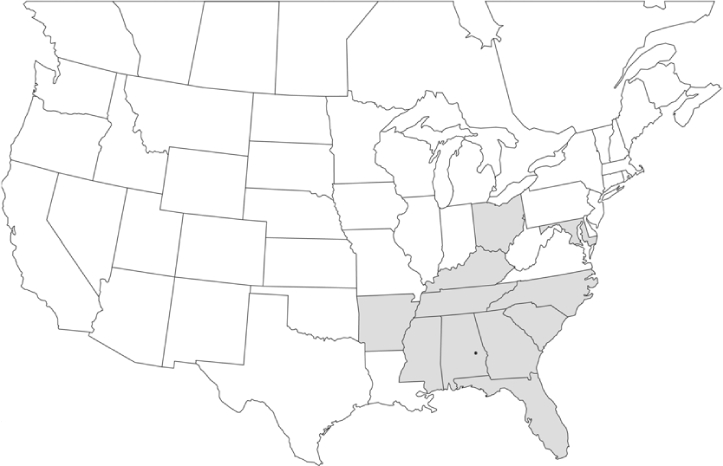
**P. diffinis** • = catch sites, n = 2 beetles Shaded areas = distributions from Luginbill and Painter, 1953

**Figure 17:**
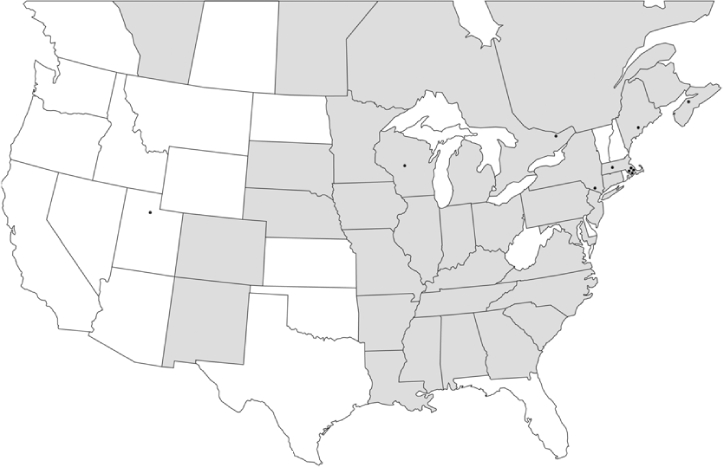
**P. drakei** • = catch sites, n = 91 beetles Shaded areas = distributions from Luginbill and Painter, 1953

**Figure 18:**
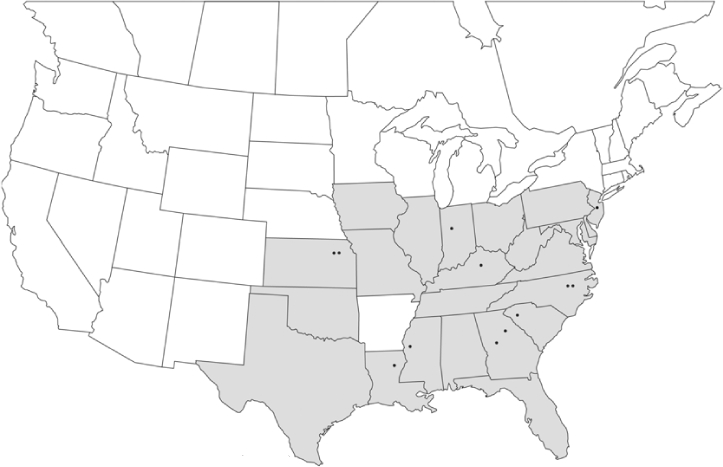
**P. ephilida** • = catch sites, n = 1726 beetles Shaded areas = distributions from Luginbill and Painter, 1953

**Figure 19:**
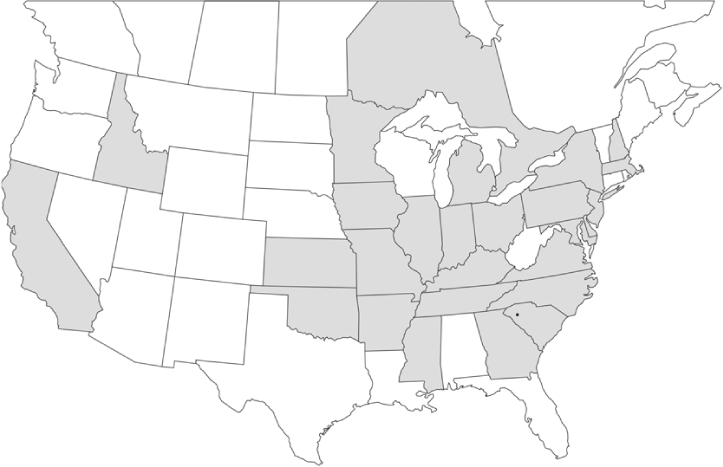
**P.fervida** • = catch sites, n = 3 beetles Shaded areas = distributions from Luginbill and Painter, 1953

**Figure 20:**
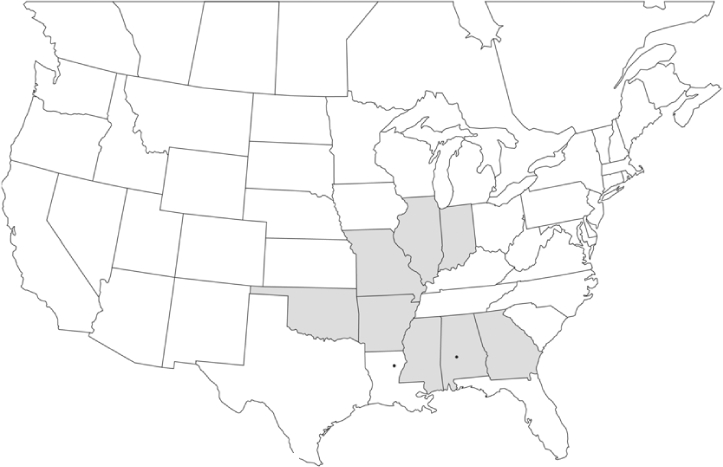
**P. forbesi** • = catch sites, n = 141 beetles Shaded areas = distributions from Luginbill and Painter, 1953

**Figure 21:**
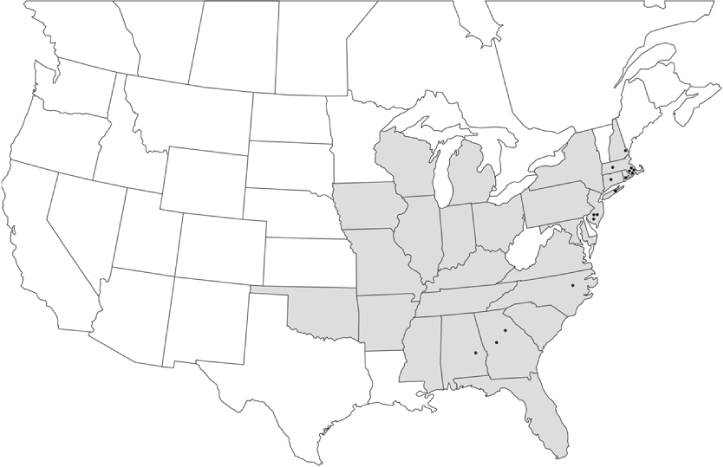
**P. forsteri** • = catch sites, n = 141 beetles Shaded areas = distributions from Luginbill and Painter, 1953

**Figure 22:**
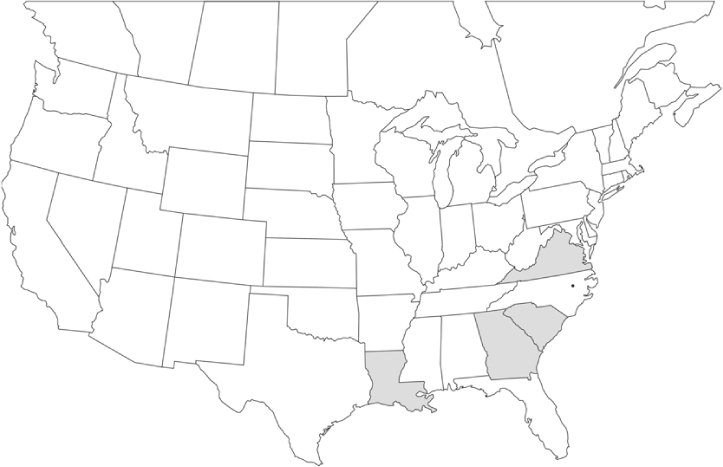
**P. foxii** • = catch sites, n = 1 beetle Shaded areas = distributions from Luginbill and Painter, 1953

**Figure 23:**
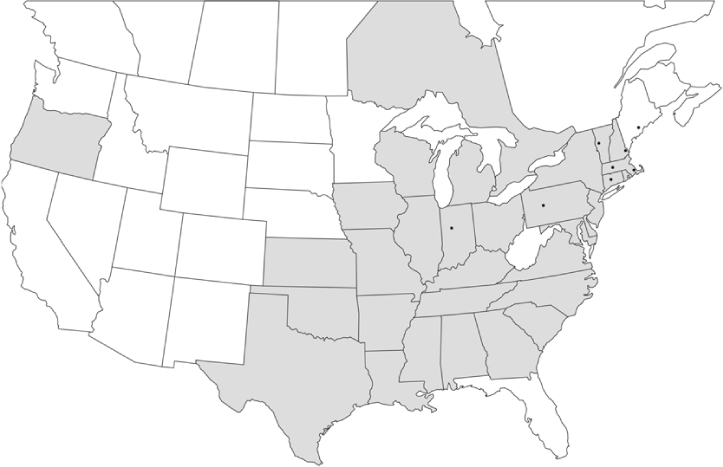
**P. fraterna** • = catch sites, n = 111 beetles Shaded areas = distributions from Luginbill and Painter, 1953

**Figure 24:**
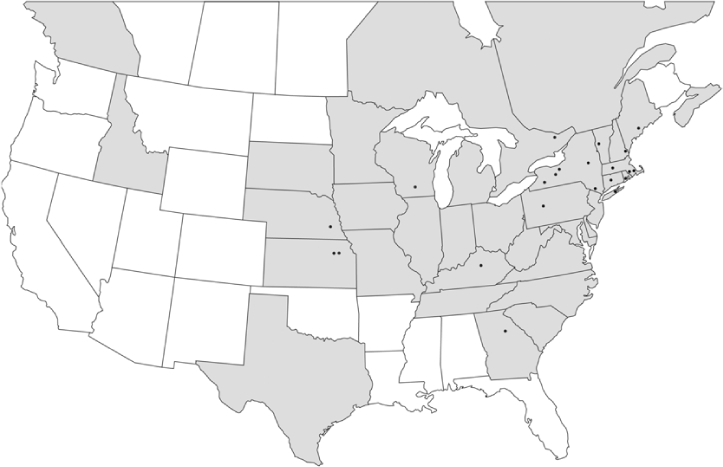
**P. fusca** • = catch sites, n = 1040 beetles Shaded areas = distributions from Luginbill and Painter, 1953

**Figure 25:**
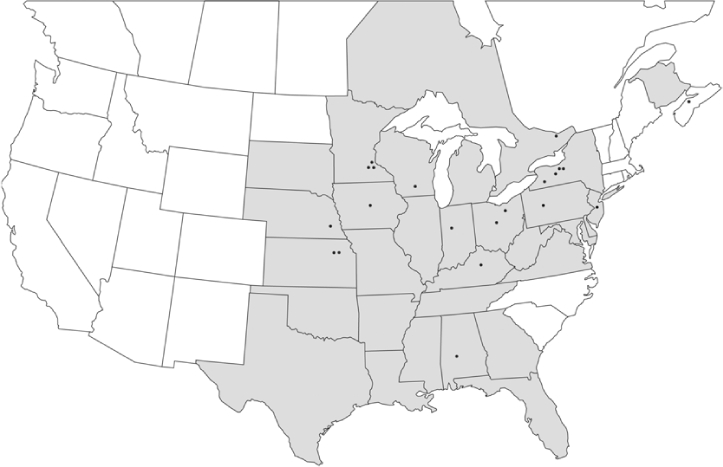
**P. futilis** • = catch sites, n = 7575 beetles Shaded areas = distributions from Luginbill and Painter, 1953

**Figure 26:**
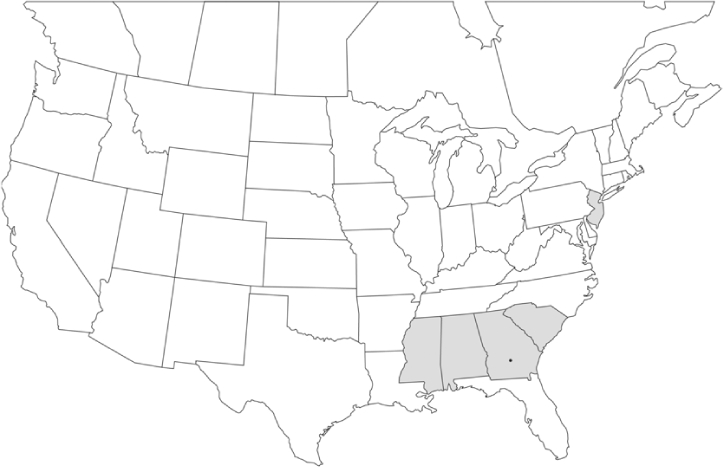
**P. (Phytalus) georgiana** • = catch sites, n = 2 beetles Shaded areas = distributions from Woodruff and Beck, 1988

**Figure 27:**
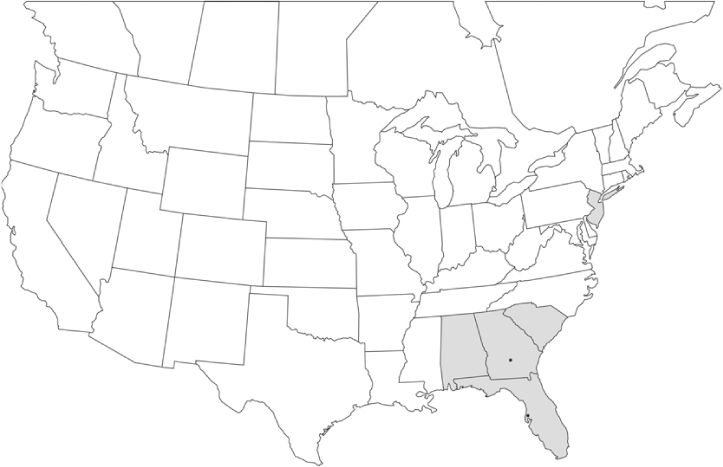
**P glaberrima** • = catch sites, n = 70 beetles Shaded areas = distributions from Luginbill and Painter, 1953

**Figure 28:**
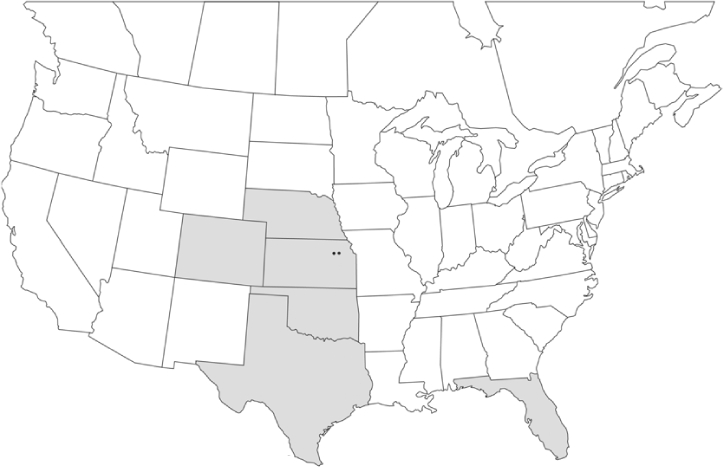
**P glabricula** • = catch sites, n = 274 beetles Shaded areas = distributions from Luginbill and Painter, 1953

**Figure 29:**
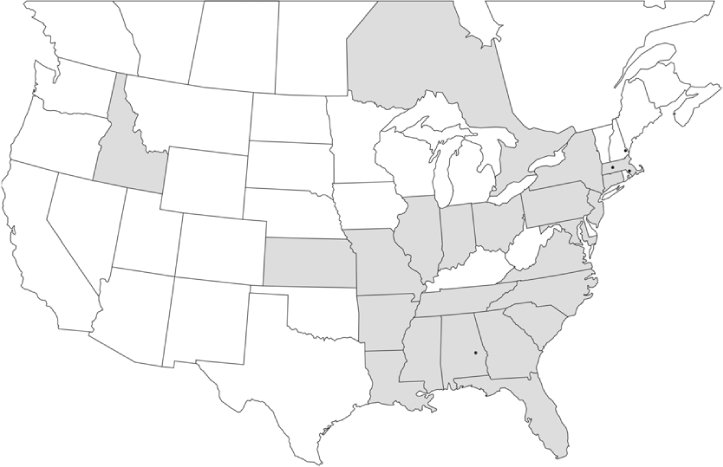
**P. gracilis** • = catch sites, n= 1930 beetles Shaded areas = distributions from Luginbill and Painter, 1953

**Figure 30:**
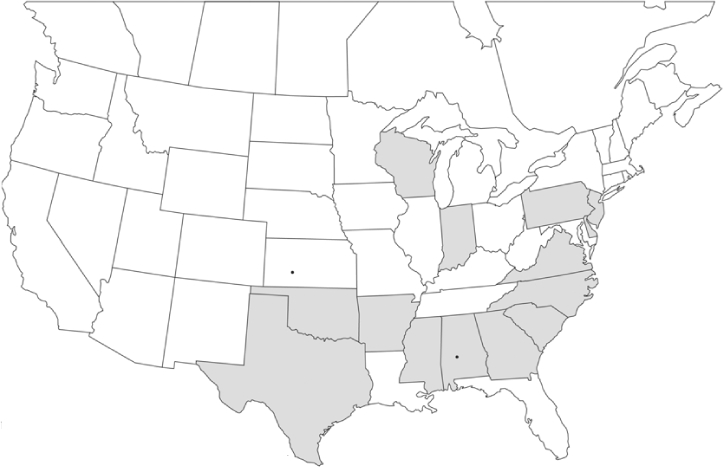
**P. gracilis** var **angulata** • = catch sites, n = 6 beetles Shaded areas = distributions from Luginbill and Painter, 1953

**Figure 31:**
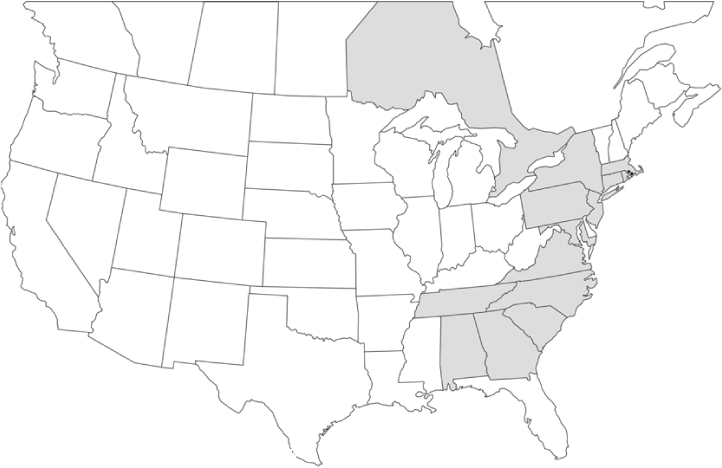
**P. hirsuta** • = catch sites, n = 2 beetles Shaded areas = distributions from Luginbill and Painter, 1953

**Figure 32:**
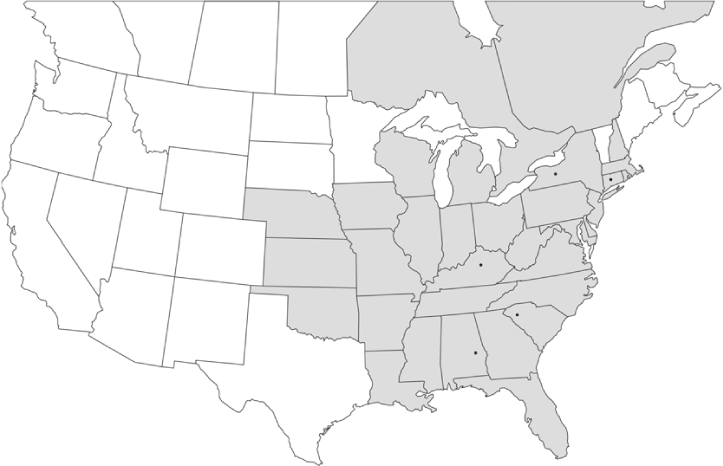
**P. hirticula** • = catch sites, n = 191 beetles Shaded areas = distributions from Luginbill and Painter, 1953

**Figure 33:**
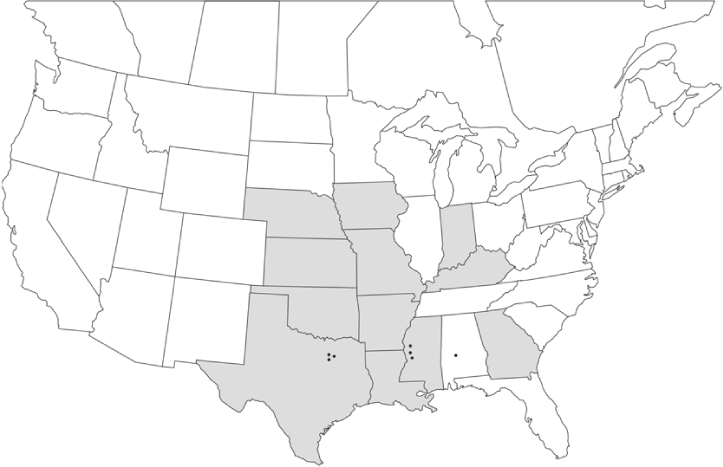
**P. hirtiventris** • = catch sites, n = 1820 beetles Shaded areas = distributions from Luginbill and Painter, 1953

**Figure 34:**
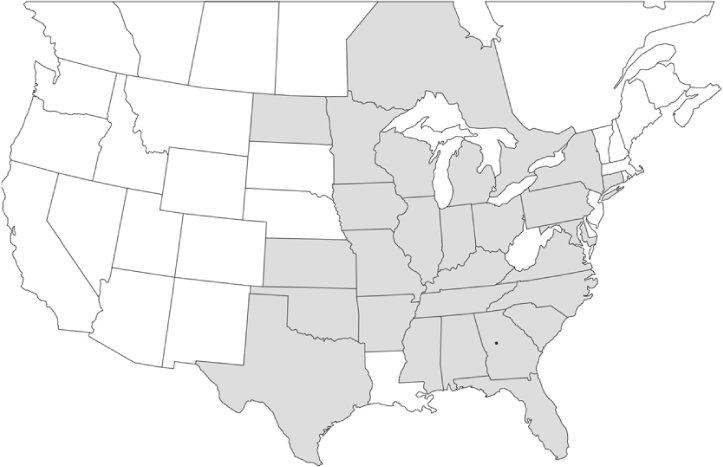
**P. ilicis** • = catch sites, n = 1 beetle Shaded areas = distributions from Luginbill and Painter, 1953

**Figure 35:**
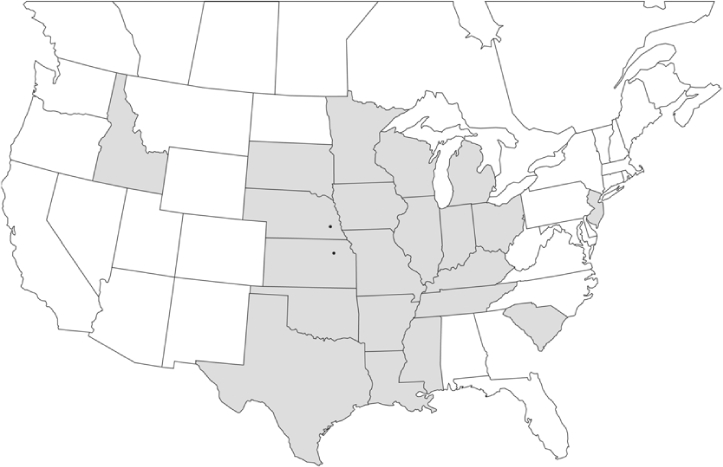
**P. implicita** • = catch sites, n = 3 beetles Shaded areas = distributions from Luginbill and Painter, 1953

**Figure 36:**
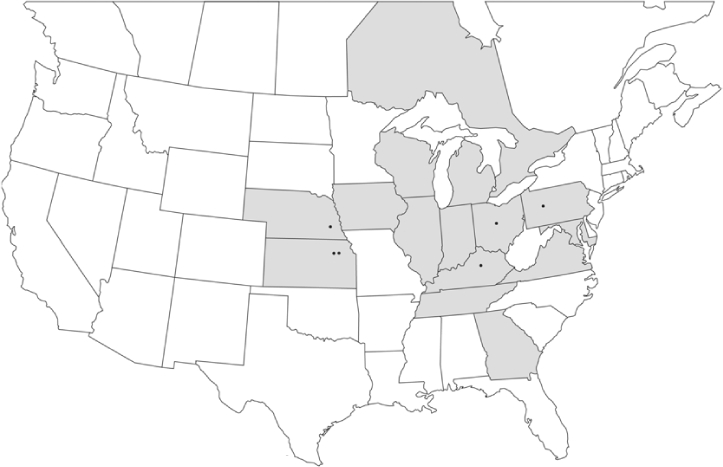
**P. inversa** • = catch sites, n = 452 beetles Shaded areas = distributions from Luginbill and Painter, 1953

**Figure 37:**
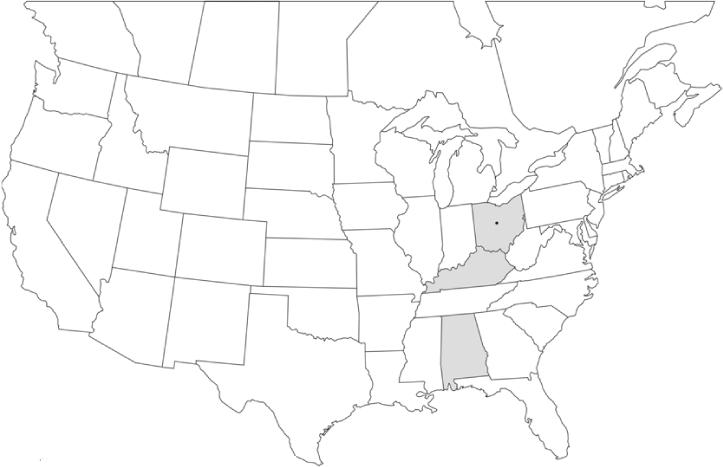
**P. kentuckiana** • = catch sites, n = 1 beetle Shaded areas = distributions from Luginbill and Painter, 1953

**Figure 38:**
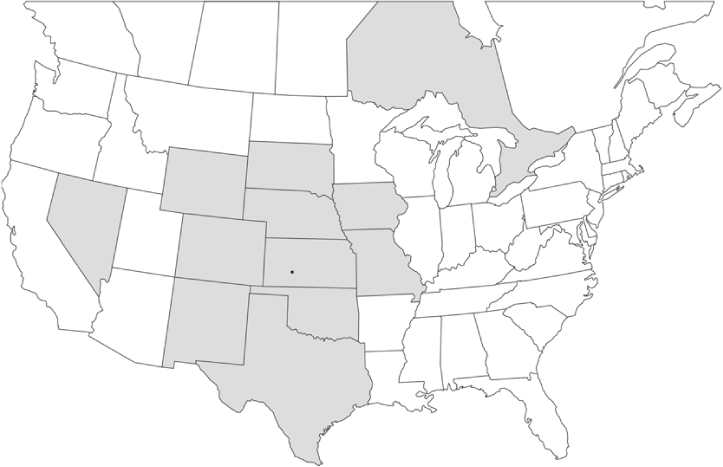
**P. lanceolata** • = catch sites, n = 1 beetle Shaded areas = distributions from Luginbill and Painter, 1953

**Figure 39:**
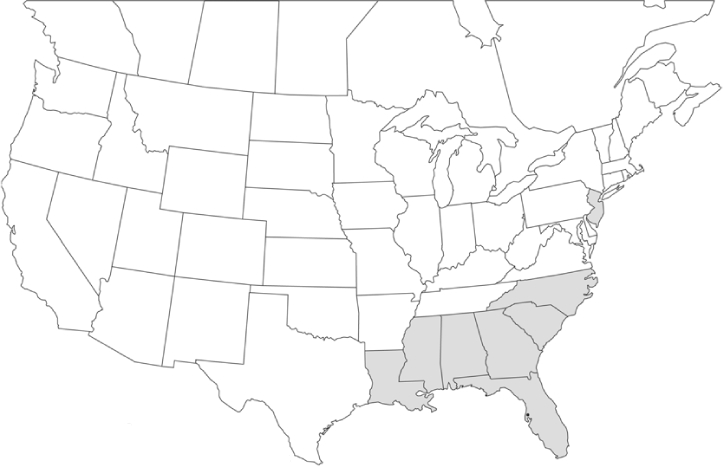
**P. latifrons** • = catch sites, n = 13 beetles Shaded areas = distributions from Luginbill and Painter, 1953

**Figure 40:**
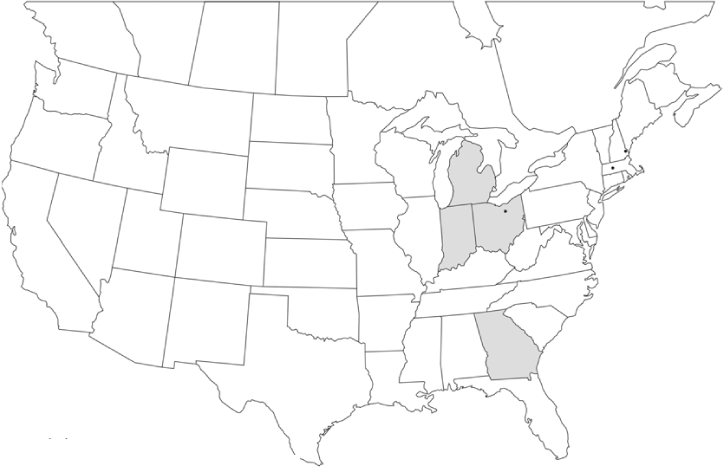
**P. longispina** • = catch sites, n = 106 beetles Shaded areas = distributions from Luginbill and Painter, 1953

**Figure 41:**
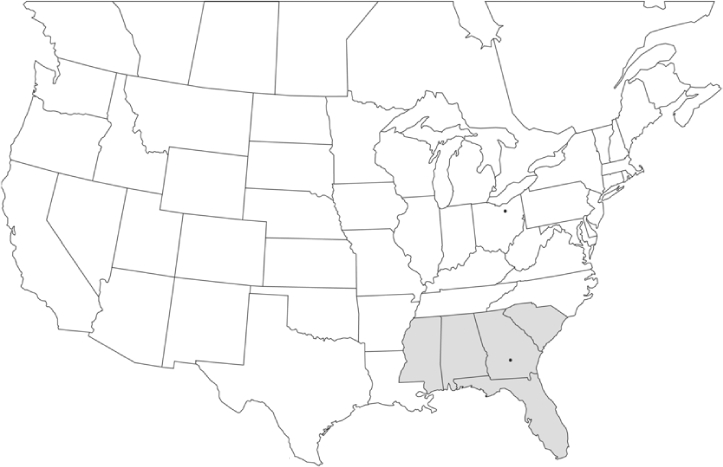
**P. lota** • = catch sites, n = 18 beetles Shaded areas = distributions from Luginbill and Painter, 1953

**Figure 42:**
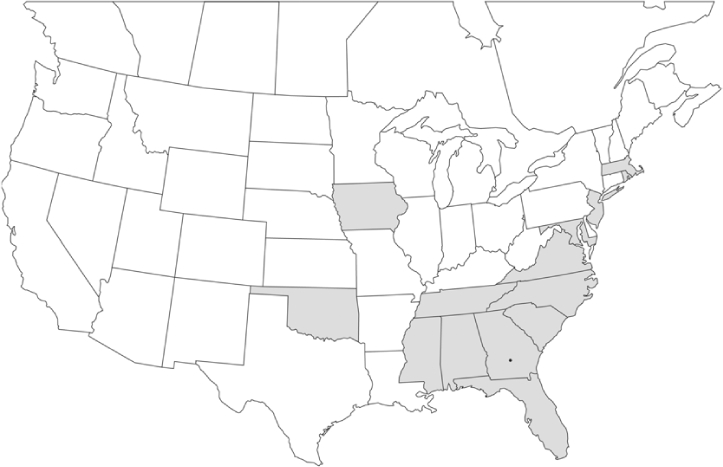
**P. luctuosa** • = catch sites, n = 1 beetle Shaded areas = distributions from Luginbill and Painter, 1953

**Figure 43:**
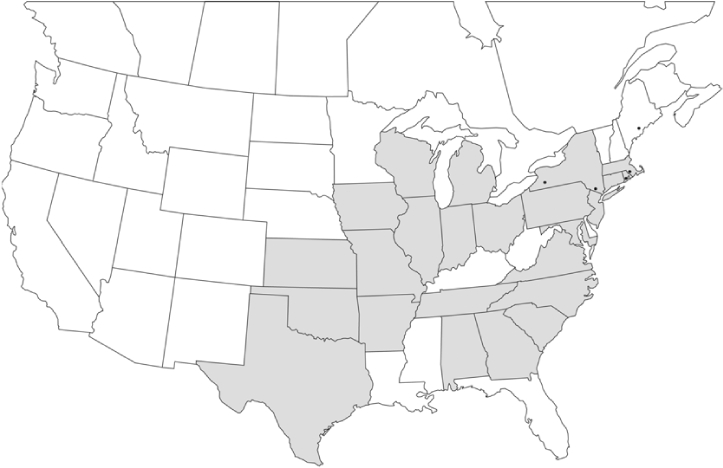
**P. marginalis** • = catch sites, n = 15 beetles Shaded areas = distributions from Luginbill and Painter, 1953

**Figure 44:**
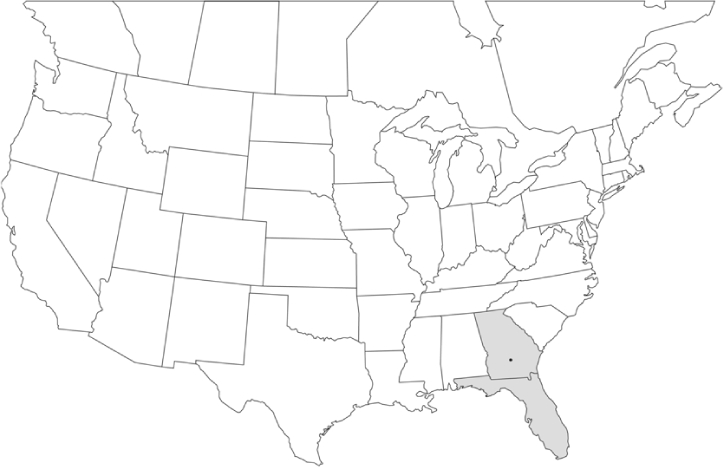
**P. mariana** • = catch sites, n = 4 beetles Shaded areas = distributions from Luginbill and Painter, 1953

**Figure 45:**
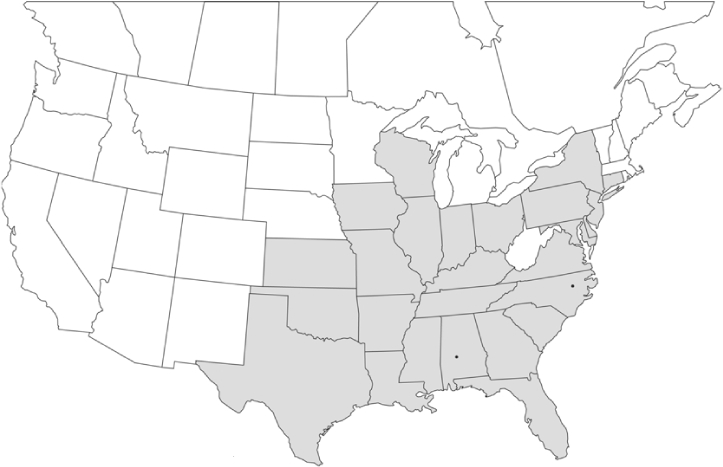
**P. micans** • = catch sites, n = 172 beetles Shaded areas = distributions from Luginbill and Painter, 1953

**Figure 46:**
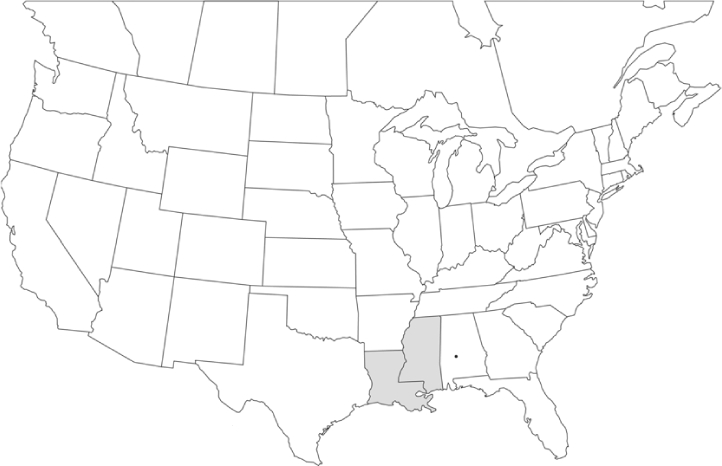
**P. (fraterna) mississippiensis** • = catch sites, n = 1 beetle Shaded areas = distributions from Luginbill and Painter, 1953

**Figure 47:**
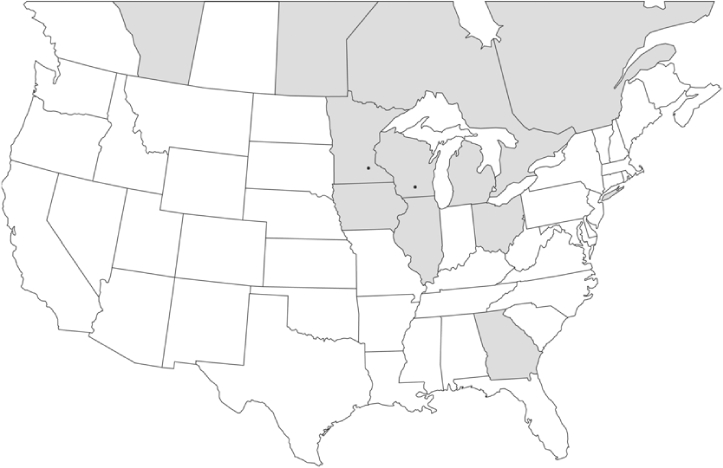
**P. nitida** • = catch sites, n = 2 beetles Shaded areas = distributions from Luginbill and Painter, 1953

**Figure 48:**
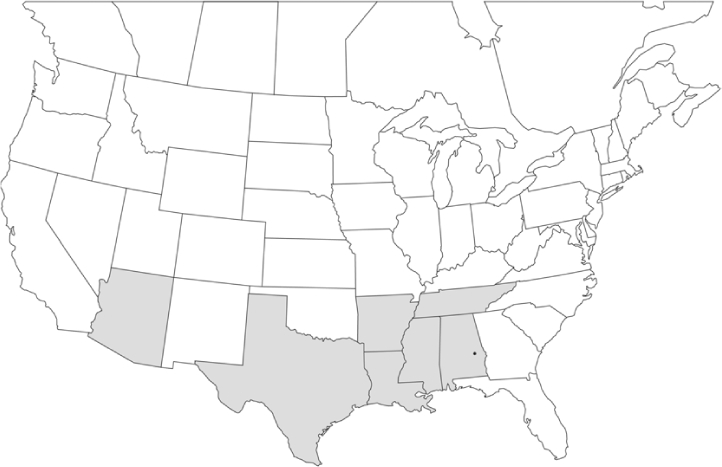
**P. obsoleta** • = catch sites, n = 22 beetles Shaded areas = distributions from Woodruff and Beck, 1988

**Figure 49:**
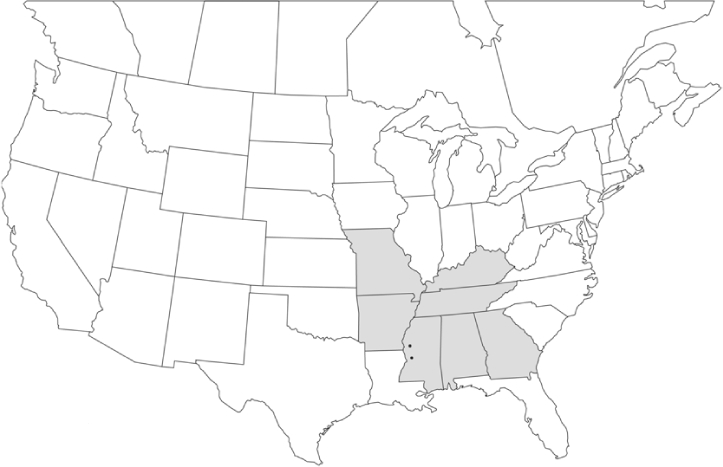
**P. perlonga** • = catch sites, n = 2 beetles Shaded areas = distributions from Luginbill and Painter, 1953

**Figure 50:**
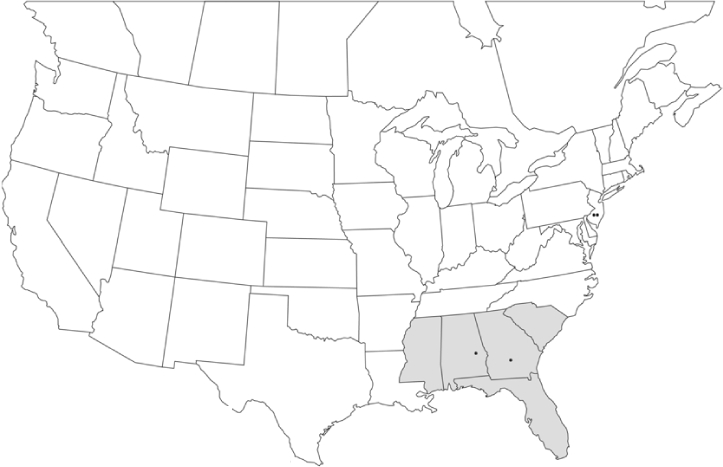
**P. postrema** • = catch sites, n = 632 beetles Shaded areas = distributions from Luginbill and Painter, 1953

**Figure 51:**
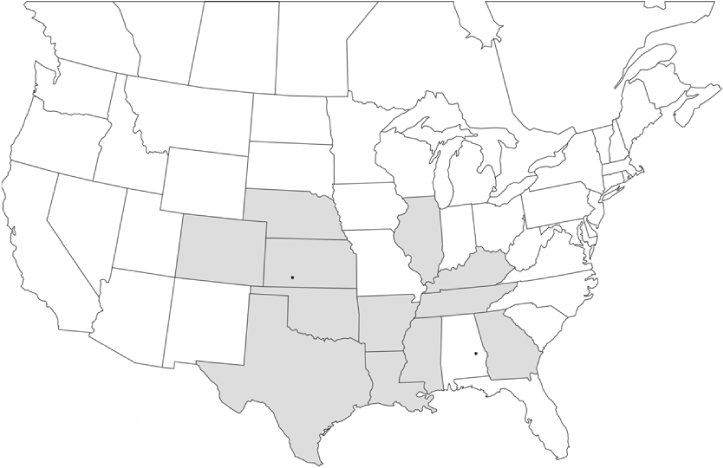
**P. praetermissa** • = catch sites, n = 2381 beetles Shaded areas = distributions from Luginbill and Painter, 1953

**Figure 52:**
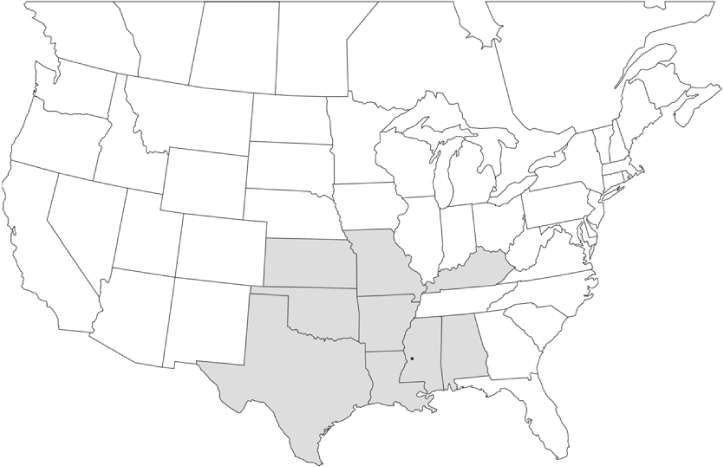
**P. profunda** • = catch sites, n = 1 beetle Shaded areas = distributions from Luginbill and Painter, 1953

**Figure 53:**
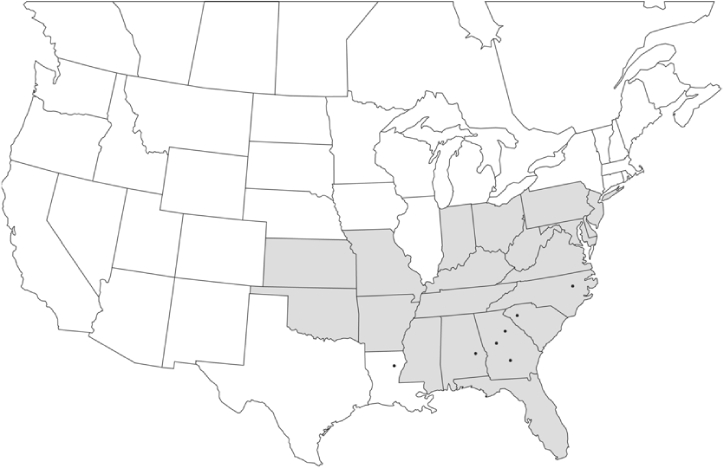
**P. quercus** • = catch sites, n = 149 beetles Shaded areas = distributions from Luginbill and Painter, 1953

**Figure 54:**
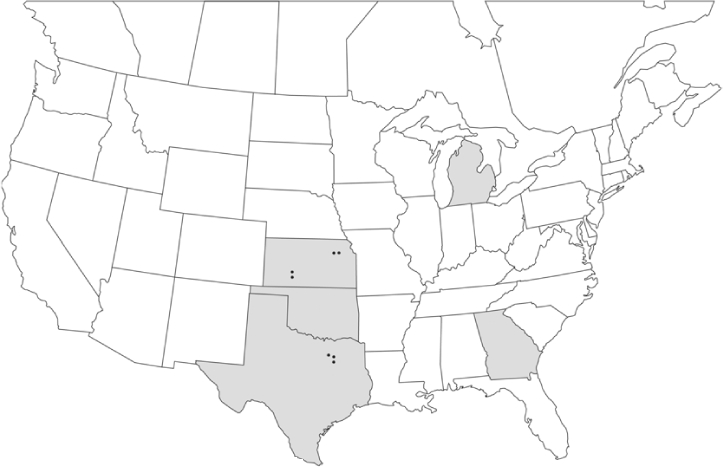
**P. rubiginosa** • = catch sites, n = 1594 beetles Shaded areas = distributions from Luginbill and Painter, 1953

**Figure 55:**
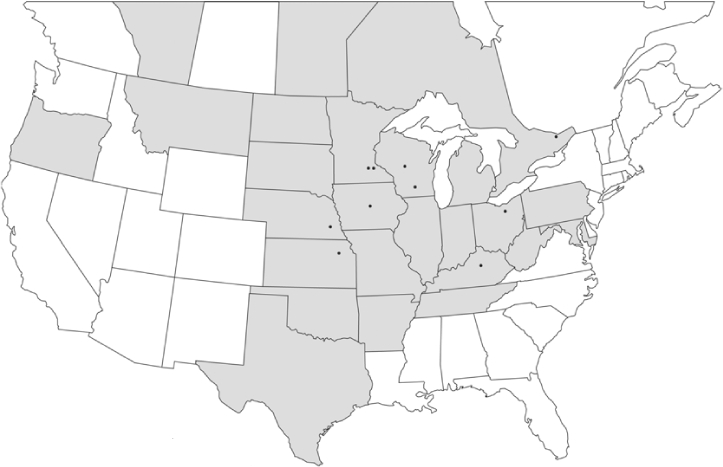
**P. rugosa** • = catch sites, n = 1393 beetles Shaded areas = distributions from Luginbill and Painter, 1953

**Figure 56:**
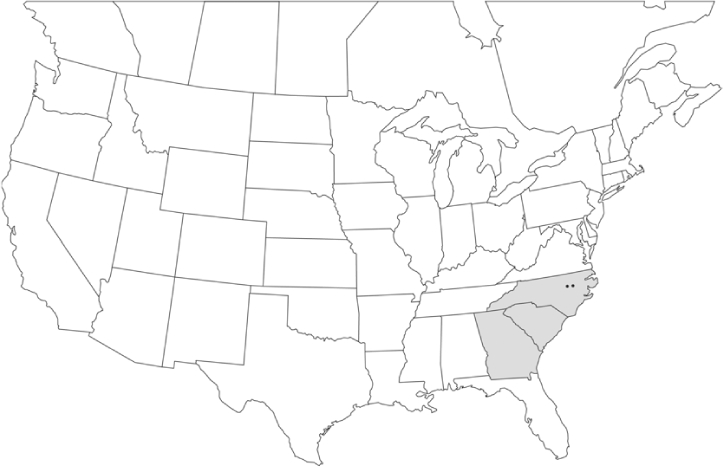
**P. soror** • = catch sites, n = 11 beetles Shaded areas = distributions from Luginbill and Painter, 1953

**Figure 57:**
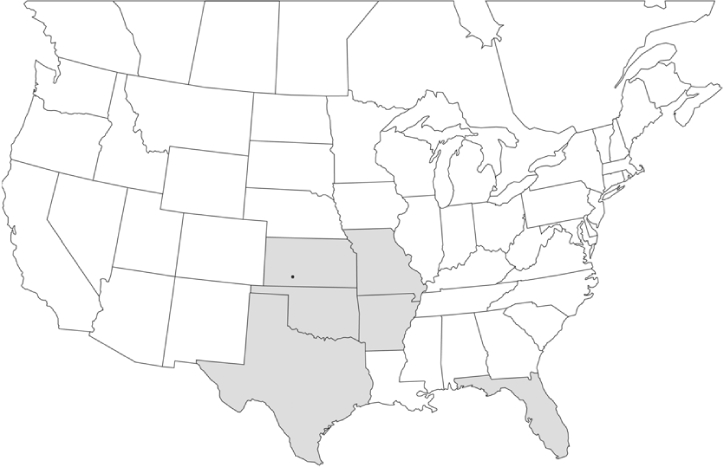
**P. submucida** • = catch sites, n = 5 beetles Shaded areas = distributions from Luginbill and Painter, 1953

**Figure 58:**
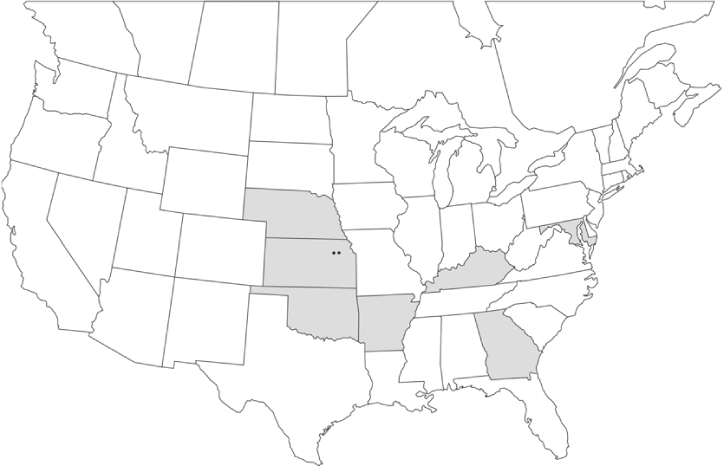
**P. sylvatica** • = catch sites, n = 104 beetles Shaded areas = distributions from Luginbill and Painter, 1953

**Figure 59:**
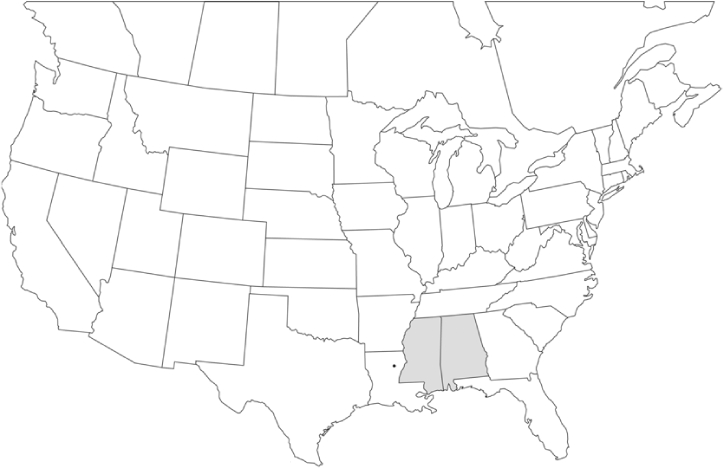
**P. taxodii** • = catch sites, n = 2 beetles Shaded areas = distributions from Luginbill and Painter, 1953

**Figure 60:**
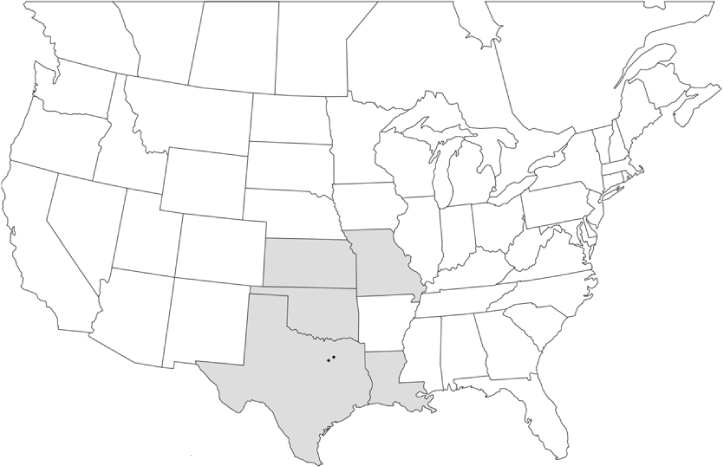
**P. torta** • = catch sites, n = 4 beetles Shaded areas = distributions from Luginbill and Painter, 1953

**Figure 61:**
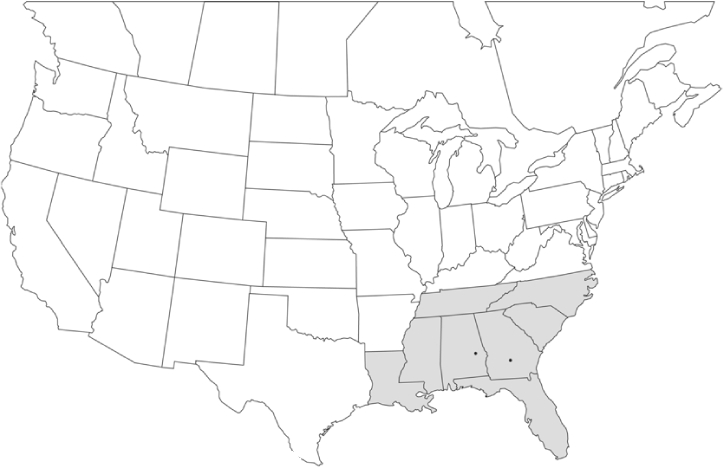
**P. ulkei** • = catch sites, n = 2 beetles Shaded areas = distributions from Luginbill and Painter, 1953

**Figure 62:**
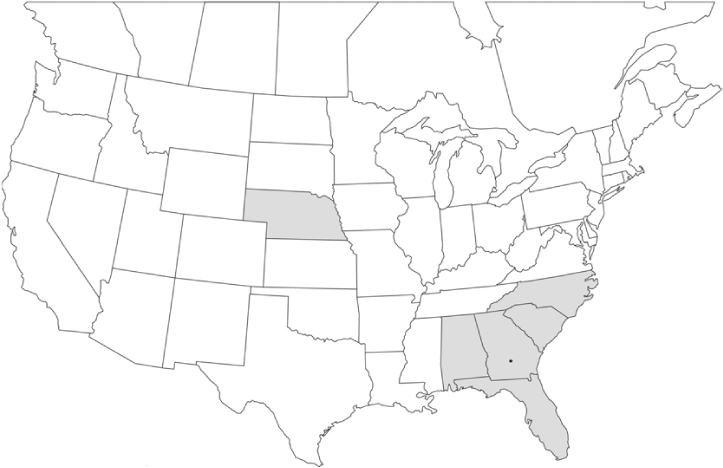
**P. uniformis** • = catch sites, n = 872 beetles Shaded areas = distributions from Luginbill and Painter, 1953

**Figure 63:**
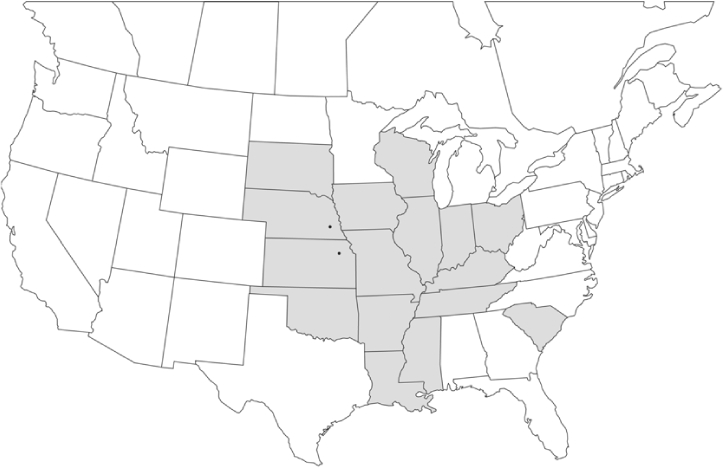
**P. vehemens** • = catch sites, n = 79 beetles Shaded areas = distributions from Luginbill and Painter, 1953

### Changes in population levels from year to year within sites

Numbers of beetles of a particular species changed dramatically from year to year at the same trapping location. For example, traps were maintained at the Auburn, Alabama site during the 1997, 1998, and 1999 seasons for approximately the same time period each year. The numbers of P. gracilis captured declined from 1123 in 1997, to 710 in 1998, and to 98 in 1999 (Tables 5, 6, 7). In Lexington, Kentucky, from 1999 to 2000, the numbers of each of the six species captured more than doubled (Tables 29, 30).

**Table 5.**
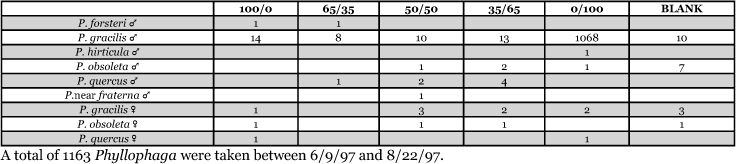
Alabama, Auburn 1997 Blends indicate the ratio of the methyl esters of L-valine/L-isoleucine

**Table 6.**
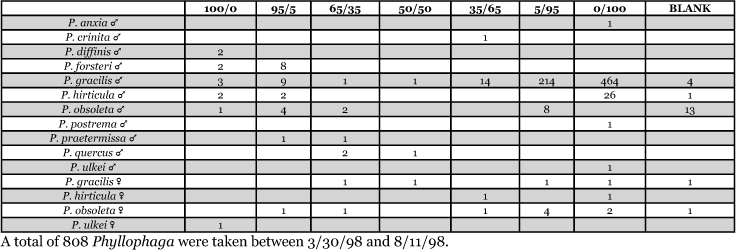
Alabama, Auburn 1998 Blends indicate the ratio of the methyl esters of L-valine/L-isoleucine

**Table 7.**
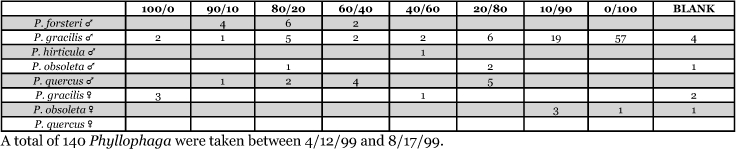
Alabama, Auburn 1999 Blends indicate the ratio of the methyl esters of L-valine/L-isoleucine

**Table 8.**
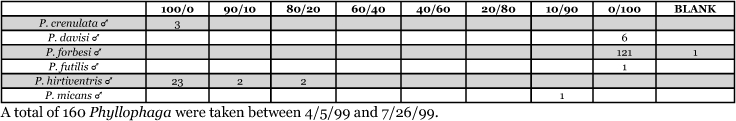
Alabama, Marion Junction 1999 Blends indicate the ratio of the methyl esters of L-valine/L-isoleucine

**Table 9.**
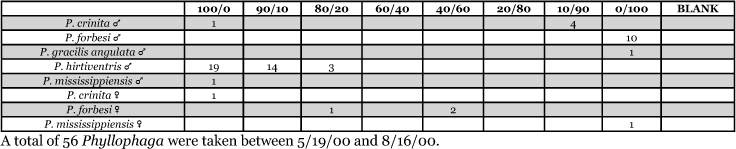
Alabama, Marion Junction 2000 Blends indicate the ratio of the methyl esters of L-valine/L-isoleucine

**Table 10.**
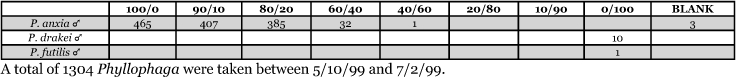
Canada, Nova Scotia, Kentville Center 1999 Blends indicate the ratio of the methyl esters of L-valine/L-isoleucine

**Table 11.**
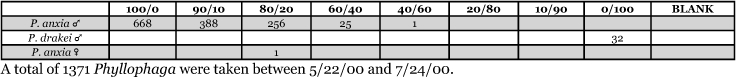
Canada, Nova Scotia, Kentville Center 2000 Blends indicate the ratio of the methyl esters of L-valine/L-isoleucine

**Table 12.**
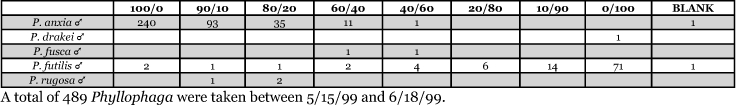
Canada, Ontario, Woodlawn 1999 Blends indicate the ratio of the methyl esters of L-valine/L-isoleucine

**Table 13.**
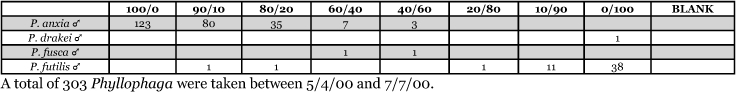
Canada, Ontario, Woodlawn 2000 Blends indicate the ratio of the methyl esters of L-valine/L-isoleucine

**Table 14.**
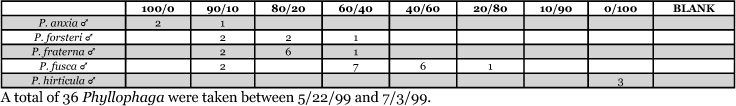
Connecticut, Vernon 1999 Blends indicate the ratio of the methyl esters of L-valine/L-isoleucine

**Table 15.**
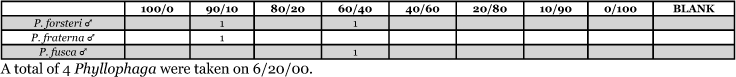
Connecticut, Vernon 2000 Blends indicate the ratio of the methyl esters of L-valine/L-isoleucine

**Table 16.**
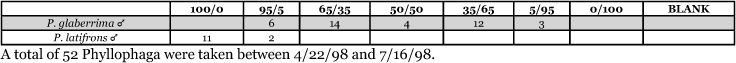
Florida, Holiday 1998 Blends indicate the ratio of the methyl esters of L-valine/L-isoleucine

**Table 17.**
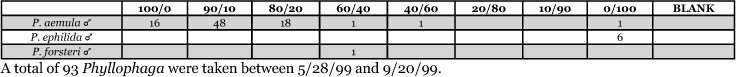
Georgia, Athens 1999 Blends indicate the ratio of the methyl esters of L-valine/L-isoleucine

**Table 18.**
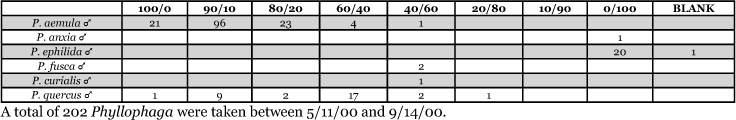
Georgia, Athens 2000 Blends indicate the ratio of the methyl esters of L-valine/L-isoleucine

**Table 19.**
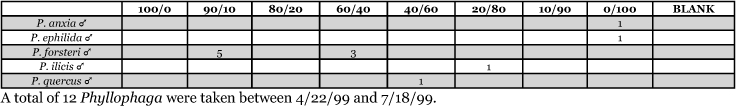
Georgia, Griffin 1999 Blends indicate the ratio of the methyl esters of L-valine/L-isoleucine

**Table 20.**
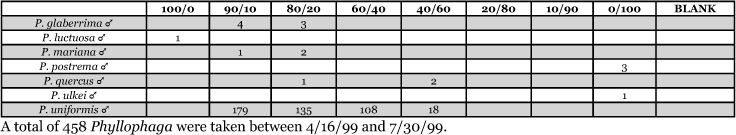
Georgia, Tifton 1999 Blends indicate the ratio of the methyl esters of L-valine/L-isoleucine

**Table 21.**
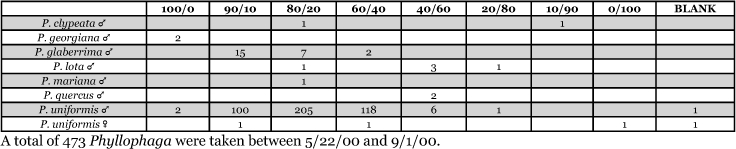
Georgia, Tifton 2000 Blends indicate the ratio of the methyl esters of L-valine/L-isoleucine

**Table 22.**
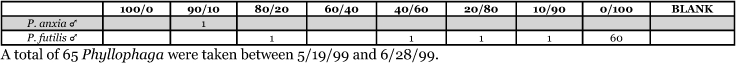
Iowa, Ames, East Reactor Woods 1999 Blends indicate the ratio of the methyl esters of L-valine/L-isoleucine

**Table 23.**
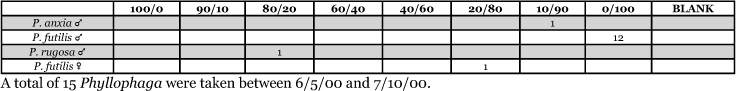
Iowa, Ames, East Reactor Woods 2000 Blends indicate the ratio of the methyl esters of L-valine/L-isoleucine

**Table 24.**
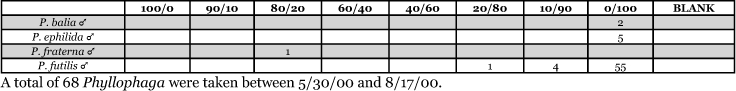
Indiana, West Lafayette 2000 Blends indicate the ratio of the methyl esters of L-valine/L-isoleucine

**Table 25.**
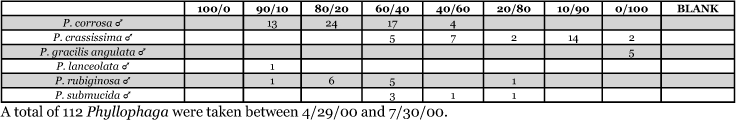
Kansas, Greensburg #1 2000 Blends indicate the ratio of the methyl esters of L-valine/L-isoleucine

**Table 26.**
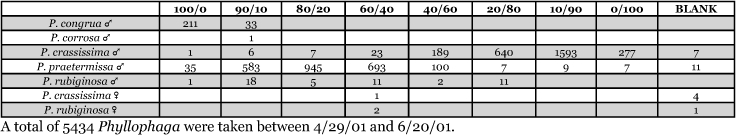
Kansas, Greensburg #2 2001 Blends indicate the ratio of the methyl esters of L-valine/L-isoleucine

**Table 27.**
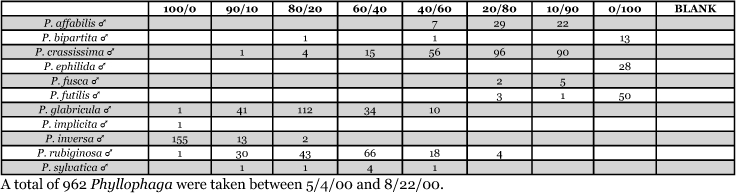
Kansas, Manhattan #1 2000 Blends indicate the ratio of the methyl esters of L-valine/L-isoleucine

**Table 28.**
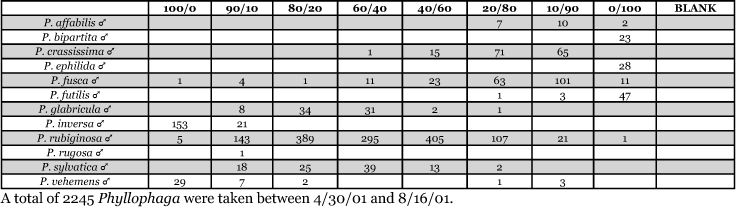
Kansas, Manhattan #2 2001 Blends indicate the ratio of the methyl esters of L-valine/L-isoleucine

**Table 29.**
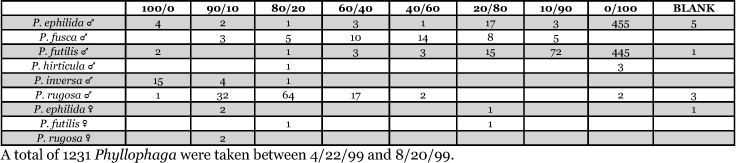
Kentucky, Lexington 1999 Blends indicate the ratio of the methyl esters of L-valine/L-isoleucine

**Table 30.**
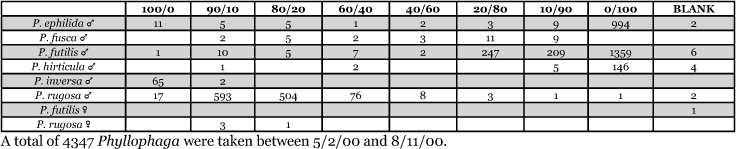
Kentucky, Lexington 2000 Blends indicate the ratio of the methyl esters of L-valine/L-isoleucine

Factors affecting the size of the population include not only weather during the flight period, but soil moisture and tilth conditions suitable for oviposition, egg hatch, and growth of larvae during the previous year or years that it takes for development to adults. Quality and quantity of larval host plants, soil textural characteristics, and species' preferences for soil types also factor into population size and distributions (Katovich et al. 1998). Changes in population size of other species at other trapping sites may be seen in Tables 5–97. Species captured at a particular site changed from year to year as well, indicating that multiyear studies will yield a more realistic picture of population sizes and species distributions than will a single year of captures.

### Male captures with sex attractants

The most important finding of the present study was the demonstration of the extensive use of the methyl esters of L-valine and L-isoleucine as sex attractants in the mate recognition systems of the Phyllophaga. In 56 discrete locations across the US and Canada, during 94 observation periods (some locations were trapped for multiple years), 61 species of Phyllophaga were captured. The overwhelming majority (58) of these species are found in the Phyllophaga (*sensu stricto*) subgenus. Since there are 147 species in this subgenus in America north of Mexico (Evans 2003; Smith and Evans 2005), 39% of the species in this group were captured in traps during the course of this study.

**Figure 64:**
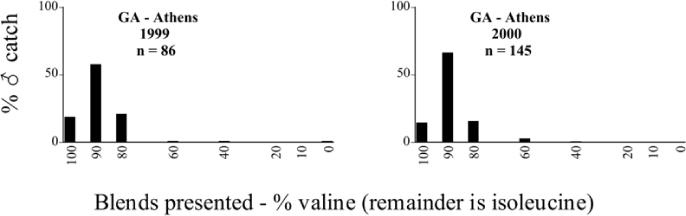
P. aemula ♂ catches

**Figure 65:**
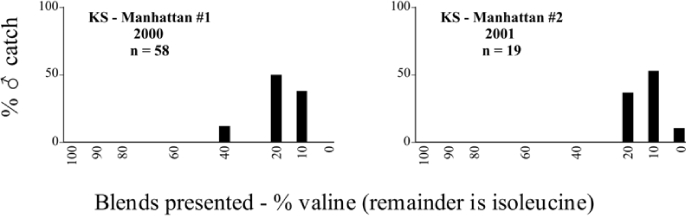
P. ajjabilis ♂ catches

Male trap captures are graphically illustrated in Figures 64–126. The figures are arranged alphabetically by Phyllophaga species. Within each species figure, graphs are arranged alphabetically by state or province abbreviation. The graphs demonstrate three general patterns of species-specific male responses to a particular blend or group of blends. The three general patterns are displayed in Figure 127. First, some species, such as P. vehemens, flew primarily to the 100/0 L-valine methyl ester/L-isoleucine methyl ester lure and were sensitive to increasing amounts of L-isoleucine methyl ester in the other blends, its presence significantly reducing captures^1^. P. congrua, however, had a broader response profile and was captured not only with the 100 % L-valine methyl ester lure, but also with blends containing 10%–20% L-isoleucine methyl ester (Figure 71). A similar situation was seen in responses of male P. hirtiventris (Figure 96). The second case (Figure 127) involved species such as P. forbesi that were captured primarily with the 100% L-isoleucine methyl ester lure. Increasing titers of L-valine methyl ester resulted in reduced or no captures^2^. In the third case (Figure 127), some species of Phyllophaga, such as P. glabricula, required the presence of both compounds before captures occurred^3^. An examination of these male- response curves reveals that, whereas certain species had a rather broad response range to the L-valine methyl ester/L-isoleucine methyl ester blends,^4^ others exhibited response curves occupying a narrower range of blends^5^.

### Specificity of response over time and space

A striking aspect of the intra-specific male flight responses to the sex attractants was their consistency between years and across geographic locations. Several species were recorded at only one site, but were captured at that site for two consecutive years^6^. The response profiles for each species in both years at those sites were nearly identical.

The majority of Phyllophaga species were recorded at more than one site (Tables 4a and 4b). As with the across-years comparison above, the intra-specific male-response curves from different geographic locations are similar as well. For instance, only five specimens of P. balia were captured during this study, but they were captured at three different locations (Figure 6) and all with the 100% L-isoleucine methyl ester lure (Figure 68). Nearly 7000 P. congrua were captured at eight different sites (Figure 9) and all sites exhibited similar response curves (Figure 71). Comparable results are seen when other species are examined^7^.

Some species were captured at only one location and only during a single year, but multiple catches over time in the same or nearby blends furnish a series of independent observations that support responses to a particular lure despite the small numbers. For instance, six specimens of P. davisi Langston were captured in Marion Junction, Alabama in 1999, all in traps baited with the 100% L-isoleucine methyl ester lure (Figure 77) on six different dates between 4/5 and 4/28. P. davisi is a species that Luginbill and Painter (1953) indicate is rare, with “Only a few specimens seen.”

In Auburn, AL in 1998 two specimens of P. diffinis (Blanchard) were captured with the 100% L-valine methyl ester lure (Figure 78) - one taken on 4/16 and one taken on 4/21.

Another uncommon species, P. (Phytalus) georgiana Horn, was captured in Tifton, Georgia, in 2000. Two individuals of this species flew to the 100% L-valine methyl ester lure (Figure 89), one on 7/17 and one on 7/21. Woodruff and Beck (1989) report that no adult host plants are recorded, the larva is undescribed, and the life cycle is unknown. This species is one of seven North American species in the subgenus Phytalus. Members of this subgenus can be discriminated from the Phyllophaga (*sensu stricto*) by their cleft tarsal claws.

P. mariana is reported as a very rare species (Luginbill and Painter 1953). Four specimens were captured in Tifton, Georgia, in 2000 and 2001, on four different dates, in traps baited with the 90/10 or 80/20 L-valine methyl ester/L-isoleucine methyl ester blends (Figure 107).

Two specimens of P. taxodii Langston were captured in Louisiana in 1997 in the 35/65 L-valine methyl ester/L-isoleucine methyl ester blend (Figure 122), one on 7/3 and the other on 8/15. Luginbill and Painter (1953) list this species as uncommon, having been captured only in AL and MS. However, Riley (1988) reports that this species is not frequently taken at lights and that fair numbers have been captured in flight-intercept traps 50 feet above the ground in cypress stands. Riley concludes that a lack of light trap catches gives the impression of rarity but that the method of collection may be more important.

Similarly, R. J. Bauernfiend in Manhattan, Kansas indicated that in many years of light trapping he had never captured P. sylvatica, and was surprised to capture 104 individuals of this species during two years of sex attractant trapping (Figure 121).

Of some interest is the capture of both P.gracilis and P.gracilis variety angulata in traps baited with 100% L-isoleucine methyl ester (Figures 92 and 93). Woodruff and Beck (1989) indicate that “The exact status of this form (angulata) awaits further study”. The different forms of the male genitalia easily separate these populations of P. gracilis. Photos of the two genitalic forms can be seen in Luginbill and Painter (1953). The two forms are sympatric over a large range (Figures 29 and 30). Langston (1927) provides illustrations of the genitalia indicating what appears to be a form intermediate between P. gracilis and P. gracilis variety angulata.

### Intra-location species interactions

The location tables (Tables 5–97) list the blends presented at each site in a particular trapping year and the numbers of Phyllophaga captured with each blend. At each site, the listed species are, by definition, sympatric. At some sites, sympatric species displayed asynchronous flight patterns, including species captured days, weeks, or in some cases, months apart. At other sites, different species of Phyllophaga were synchronic as well as sympatric, but may or may not have been captured with the same sex attractant blends. The distinction of whether males of different species were or were not captured in the same blends is important because it can aid in identifying locations where inter-specific competition for pheromonal space may be occurring. Congeneric males flying to the same sex attractant blends indicate situations where there is potential for inter-specific mating interactions that afford opportunities for investigation into reproductive isolation involving additional species-specific sex attractant compounds, close-range mating behavior, and/or mating at different times.

**Table 31.**
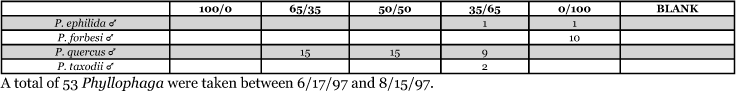
Louisiana, Chase 1997 Blends indicate the ratio of the methyl esters of L-valine/L-isoleucine

**Table 32.**
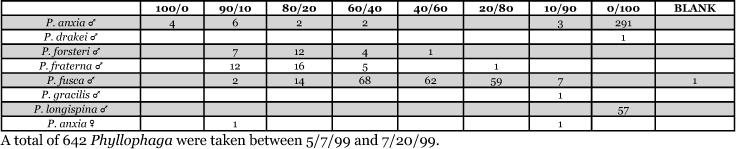
Massachusetts, South Amherst 1999 Blends indicate the ratio of the methyl esters of L-valine/L-isoleucine

**Table 33.**
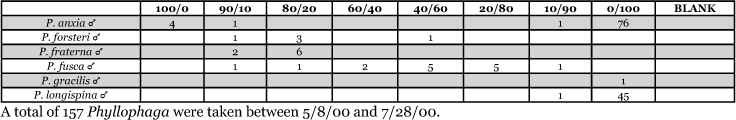
Massachusetts, South Amherst 2000 Blends indicate the ratio of the methyl esters of L-valine/L-isoleucine

**Table 34.**
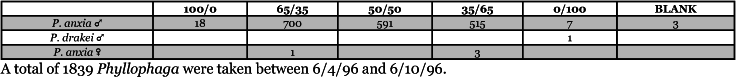
Massachusetts, Carver 1996 Blends indicate the ratio of the methyl esters of L-valine/L-isoleucine

**Table 35.**
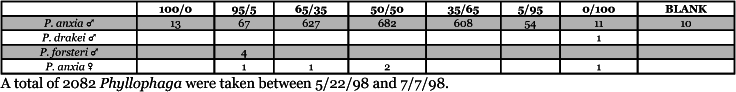
Massachusetts, Carver 1998 Blends indicate the ratio of the methyl esters of L-valine/L-isoleucine

**Table 36.**
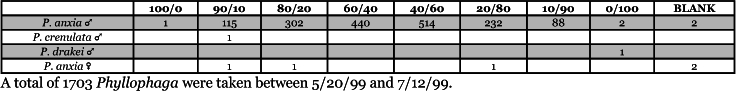
Massachusetts, Carver 1999 Blends indicate the ratio of the methyl esters of L-valine/L-isoleucine

**Table 37.**
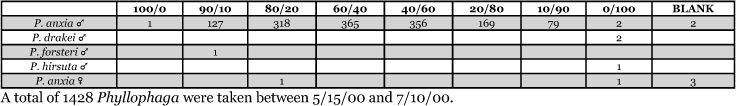
Massachusetts, Carver 2000 Blends indicate the ratio of the methyl esters of L-valine/L-isoleucine

**Table 38.**
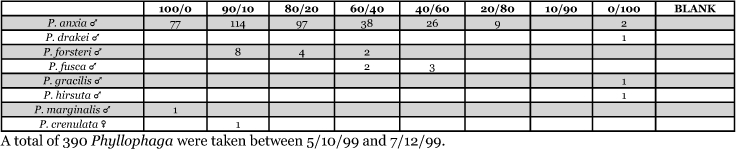
Massachusetts, Lakeville 1999 Blends indicate the ratio of the methyl esters of L-valine/L-isoleucine

**Table 39.**
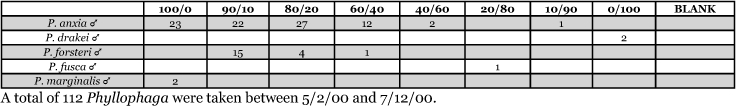
Massachusetts, Lakeville 2000 Blends indicate the ratio of the methyl esters of L-valine/L-isoleucine

**Table 40.**
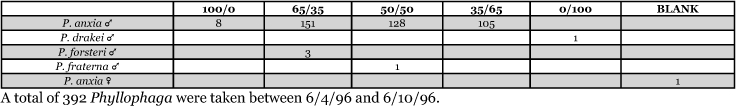
Massachusetts, Plympton #1 1996 Blends indicate the ratio of the methyl esters of L-valine/L-isoleucine

**Table 41.**
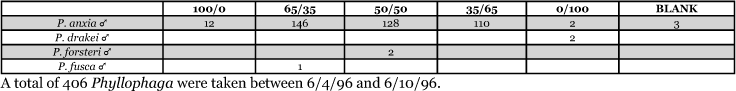
Massachusetts, Plympton #2 1996 Blends indicate the ratio of the methyl esters of L-valine/L-isoleucine

**Table 42.**
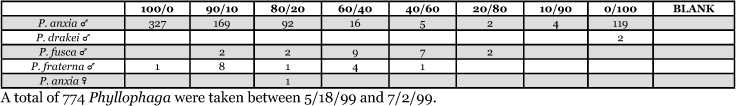
Maine, Lincolnville Center 1999 Blends indicate the ratio of the methyl esters of L-valine/L-isoleucine

**Table 43.**
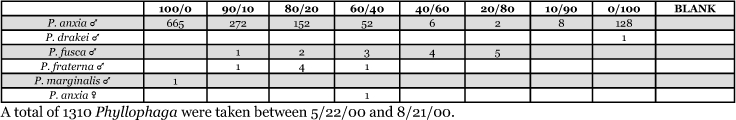
Maine, Lincolnville Center 2000 Blends indicate the ratio of the methyl esters of L-valine/L-isoleucine

**Table 44.**
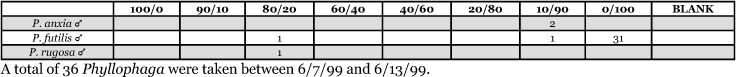
Minnesota, St. Paul #1 1999 Blends indicate the ratio of the methyl esters of L-valine/L-isoleucine

**Table 45.**
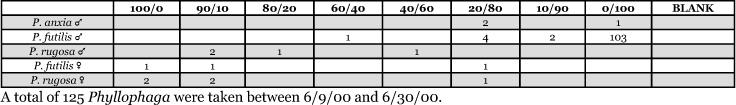
Minnesota, St. Paul #1 2000 Blends indicate the ratio of the methyl esters of L-valine/L-isoleucine

**Table 46.**
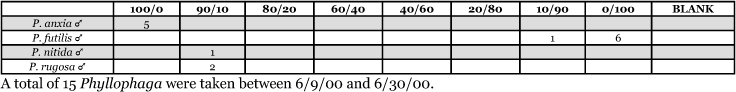
Minnesota, St. Paul #2 2000 Blends indicate the ratio of the methyl esters of L-valine/L-isoleucine

**Table 47.**
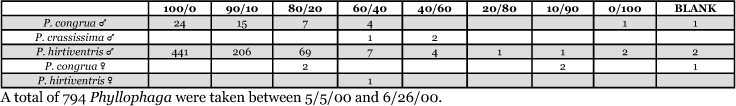
Mississippi, Leroy Percy State Park 2000 Blends indicate the ratio of the methyl esters of L-valine/L-isoleucine

**Table 48.**
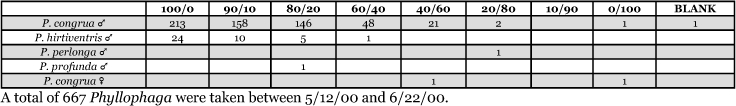
Mississippi, Sharkey County, 5 miles SE of Anguilla 2000 Blends indicate the ratio of the methyl esters of L-valine/L-isoleucine

**Table 49.**
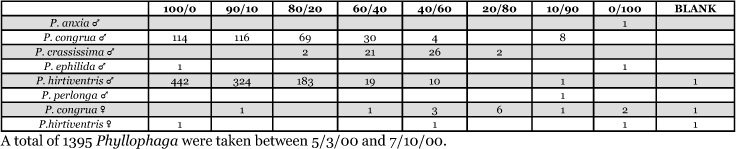
Mississippi, Stoneville 2000 Blends indicate the ratio of the methyl esters of L-valine/L-isoleucine

**Table 50.**
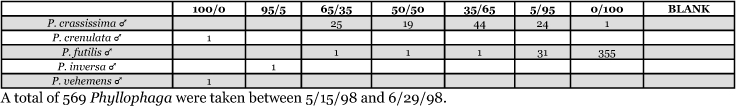
Nebraska, Lincoln 1998 Blends indicate the ratio of the methyl esters of L-valine/L-isoleucine

**Table 51.**
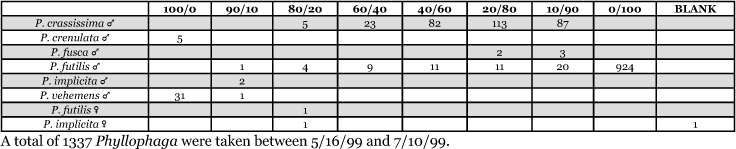
Nebraska, Lincoln 1999 Blends indicate the ratio of the methyl esters of L-valine/L-isoleucine

**Table 52.**
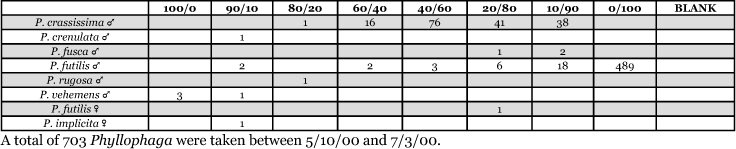
Nebraska, Lincoln 2000 Blends indicate the ratio of the methyl esters of L-valine/L-isoleucine

**Table 53.**
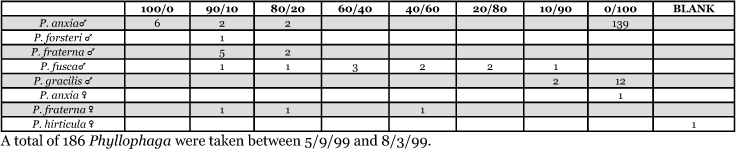
New Hampshire, Madbury 1999 Blends indicate the ratio of the methyl esters of L-valine/L-isoleucine

**Table 54.**
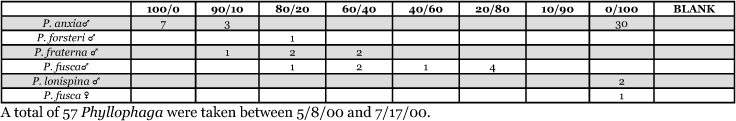
New Hampshire, Madbury 2000 Blends indicate the ratio of the methyl esters of L-valine/L-isoleucine

**Table 55.**
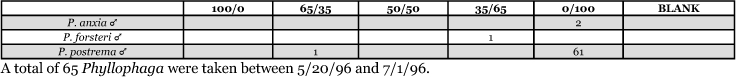
New Jersey, Chatsworth #1 1996 Blends indicate the ratio of the methyl esters of L-valine/L-isoleucine

**Table 56.**
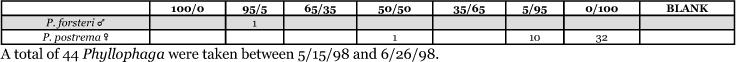
New Jersey, Chatsworth #1 1998 Blends indicate the ratio of the methyl esters of L-valine/L-isoleucine

**Table 57.**
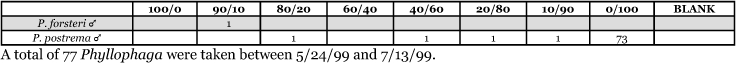
New Jersey, Chatsworth #1 1999 Blends indicate the ratio of the methyl esters of L-valine/L-isoleucine

**Table 58.**
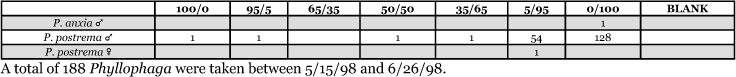
New Jersey, Chatsworth #2 1998 Blends indicate the ratio of the methyl esters of L-valine/L-isoleucine

Each of the three scenarios (1. sympatric and synchronically flying species captured using different blends; 2. sympatric and asynchronically flying species captured using the same blend; 3. sympatric and synchronically flying species captured using the same blend) were encountered in the course of this study, individually as well as in combination at different study sites. The following examples illustrate the three scenarios.

- In Lexington, Kentucky, in 1999, both P. futilis and P. rugosa flew synchronically between 5/10 and 7/7, but were captured in traps baited with different blends of L-valine methyl ester/L-isoleucine methyl ester (Figure 128).
- In Lincoln, Nebraska, in 1999, both P. vehemens and P. crenulata were captured in the trap baited with the 100% L-valine methyl ester, but their flight periods were separated by 18 days during which no males of either species were captured. P. vehemens flew from 5/16 through 5/25, whereas P. crenulata flew from 6/12 through 7/5 (Figure 129).
- In Amherst, Massachusetts, in 1999, both P. anxia and P. longispina flew to the 100% L-isoleucine methyl ester lure during the period between 5/18 and 6/15 (Figure 130). This latter scenario is the most engaging because it is in this case that the potential for conflict in terms of pheromonal space arises.

From the male capture data at the various trapping sites, many examples of different species flying synchronically, sympatrically, and to the same blends or blend groupings have been documented. However, nothing is known about whether these species fly at different times of the night or how close-range courtship behaviors are involved in mate recognition. Figures 131–133 display detailed information relating male response specificity curves and time of flight from three sites that demonstrate the complexity of interactions that can occur over a season. Figure 131 (KS Manhattan #2, 2001), demonstrates that P. inversa and P. vehemens flew synchronically to the 100% L-valine methyl ester lure. Similarly, there is potential for interaction between P. rubiginosa and P. sylvatica (middle L-valine methyl ester/L-isoleucine methyl ester blends), P. crassissima and P. fusca (lower L-valine methyl ester/L-isoleucine methyl ester blends), and P. futilis and P. bipartitia (100% L-isoleucine methyl ester lure). In the July and August flights at the same site, P. glabricula, P. affabilis, and P. ephilida were captured synchronically, but with different blends. Figure 132 (KY Lexington, 2000) shows that P. futilis and P. hirticula flew synchronically to the 100% L-isoleucine methyl ester lure, whereas P. ephilida was captured with the same lure, but later in the season. In Figure 133 (MA Amherst, 1999), synchronic species P. forsteri, P. fraterna, and P. fusca displayed overlapping male response curves to L-valine methyl ester/L-isoleucine methyl ester mixes, whereas P. anxia, P. longispina, and P. drakei also flew synchronically to the 100% L-isoleucine methyl ester lure.

Table 98 outlines, in a briefer format, other trapping locations and the species involved where inter-specific attraction might occur as a result of competition for pheromonal space. However, the possibility of interactions postulated by the overlap of the male response curves in time and space may not necessarily reflect the reality in the field in that females may or may not have a narrower range of sex pheromone blend production than is suggested by what the males are capable of responding to. The potential for inter-specific interactions might be predicted with greater accuracy by combining analysis of blend ratios produced by a number of individual females with knowledge of male captures by various blends and determining if male response curves overlap congeneric female production curves.

Interspecific copulation between Phyllophaga species has been reported in the literature. Fattig (1944) reports (p. 26) that he collected a male P. hirticula copulating with a female P. anxia. Referencing the data from the present study, P. hirticula males were captured in traps baited with the 100% L-isoleucine methyl ester lure (Figure 95). P. anxia males of both the northern and the southern genitalic form were also captured in traps baited with the 100% L-isoleucine methyl ester lure (Figures 66a, 66b, 66c, 66d, 66e and 67). It is likely that the male P. hirticula flew upwind following an L-isoleucine methyl ester plume to find not a conspecific female P. hirticula, but a congeneric female P. anxia. P. hirticula and P. anxia males possess genitalia whose cuticular structures are exceedingly different (see images in Woodruff and Beck, 1989). Their soft sac structures are very different as well (P.S. Robbins, personal observation). Their vestitures also differ, P. anxia being glabrous and P. hirticula being hirsute. Although interspecific genitalic differences could and sometimes do play a role in reproductive isolation of some taxa (Eberhard 1985, Sota and Kubota 1998), genitalic differences clearly did not prevent copulation from taking place in this case.

**Figure 66a:**
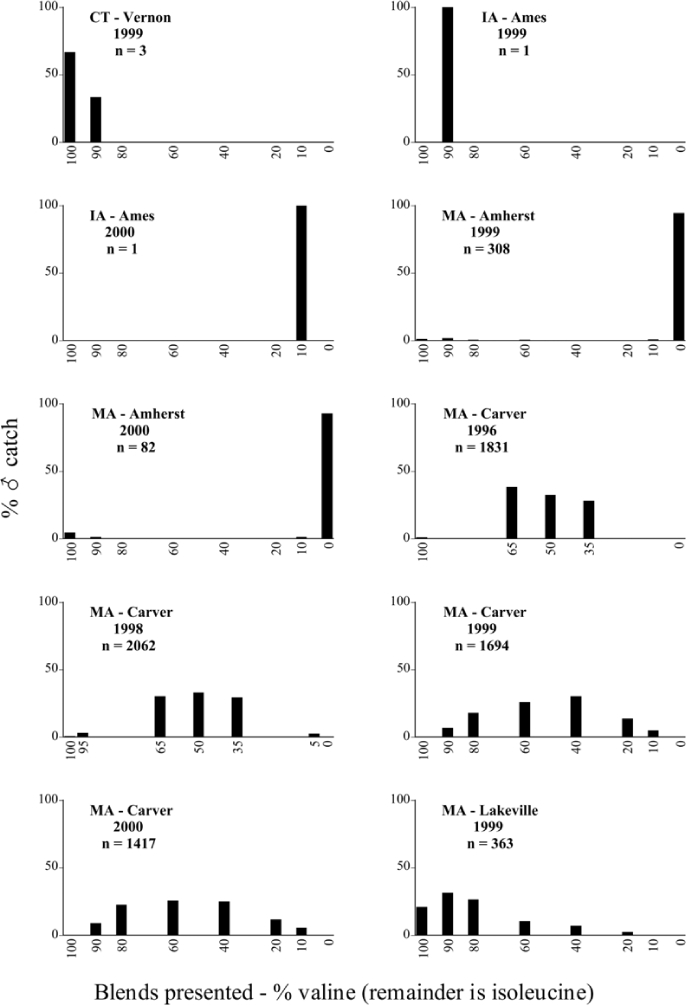
P. anxia (northern genitalic form) ♂ catches

**Figure 66b:**
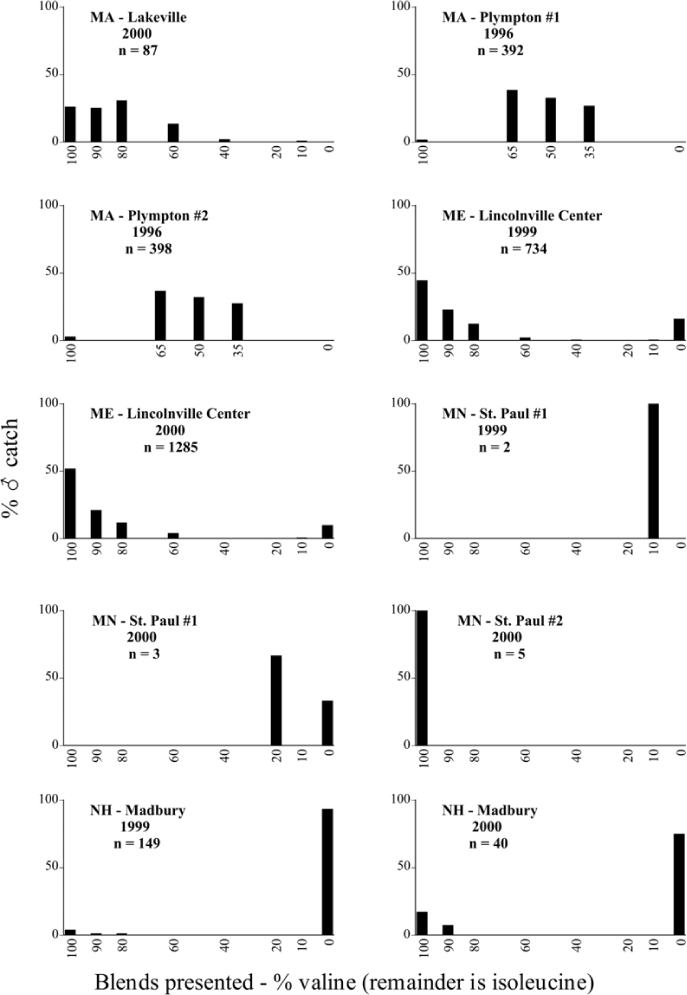
P. anxia (northern genitalic form) ♂ catches

**Figure 66c:**
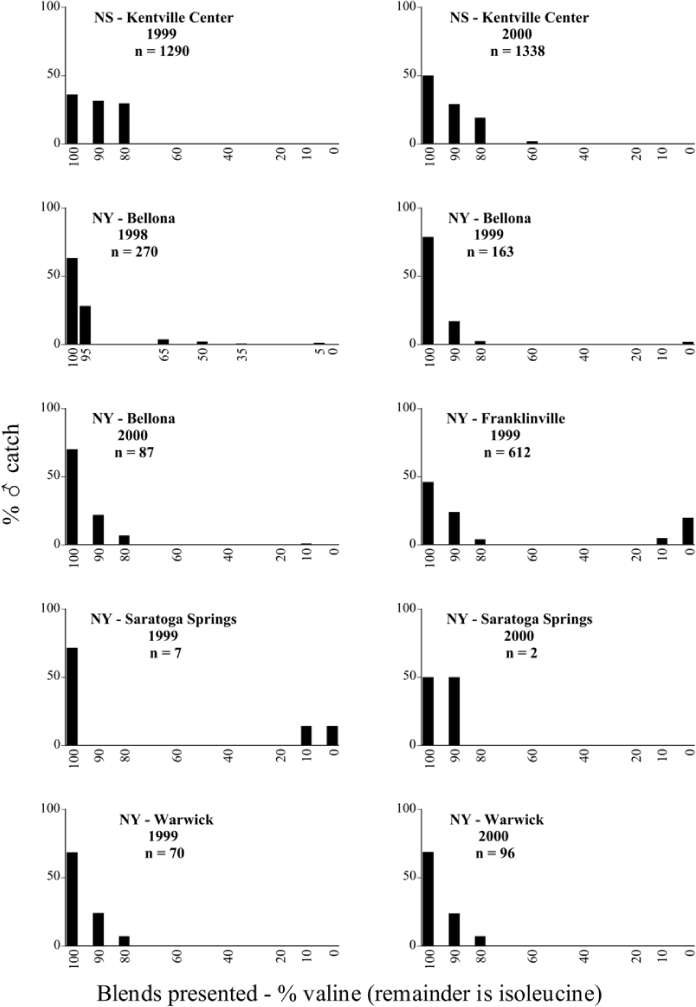
P. anxia (northern genitalic form) ♂ catches

**Figure 66d:**
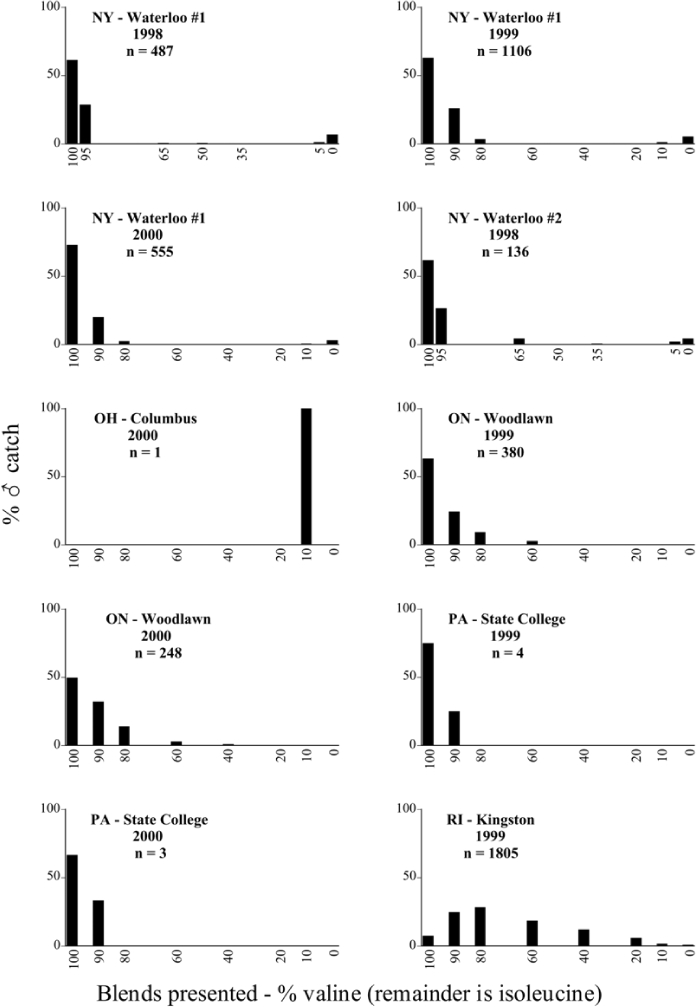
P. anxia (northern genitalic form) ♂ catches

**Figure 66e:**
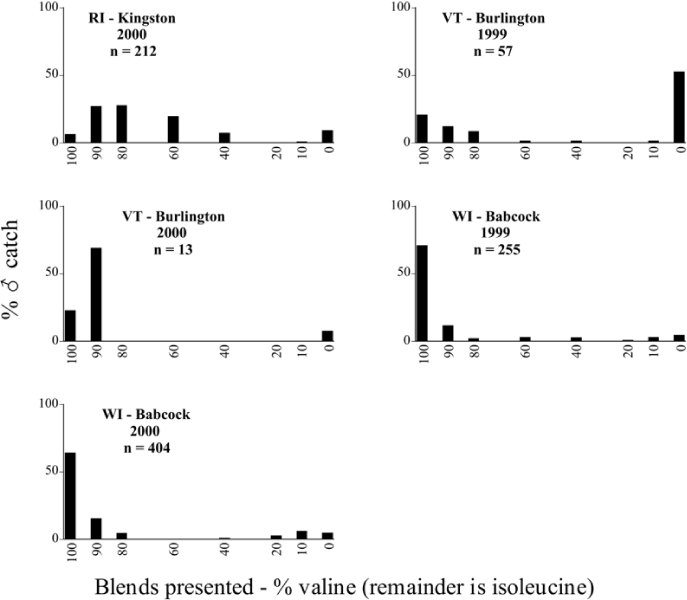
P. anxia (northern genitalic form) ♂ catches

**Figure 67:**
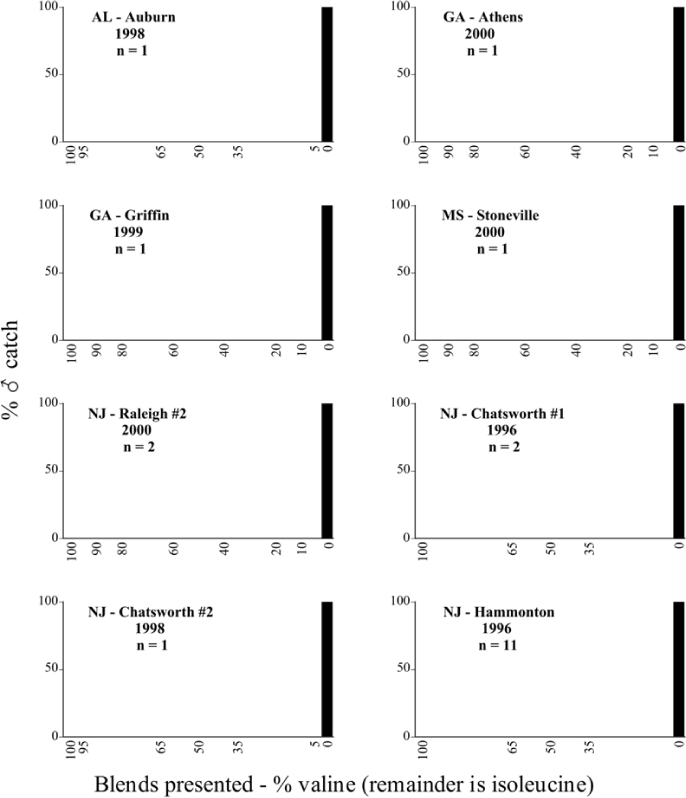
P. anxia (southern genitalic form) ♂ catches

**Figure 68:**
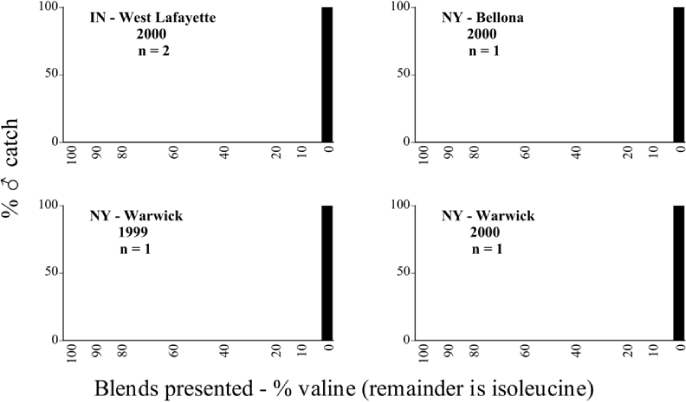
P. balia ♂ catches

**Figure 69:**
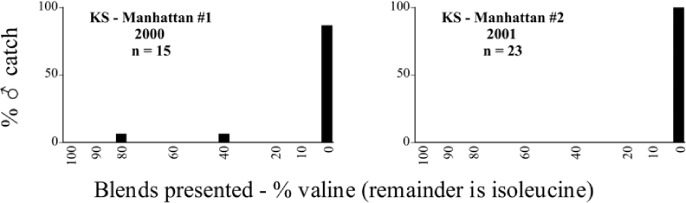
P. bipartita ♂ catches

**Figure 70:**
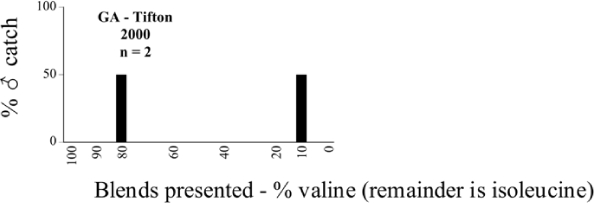
P. clypeata ♂ catches

**Figure 71:**
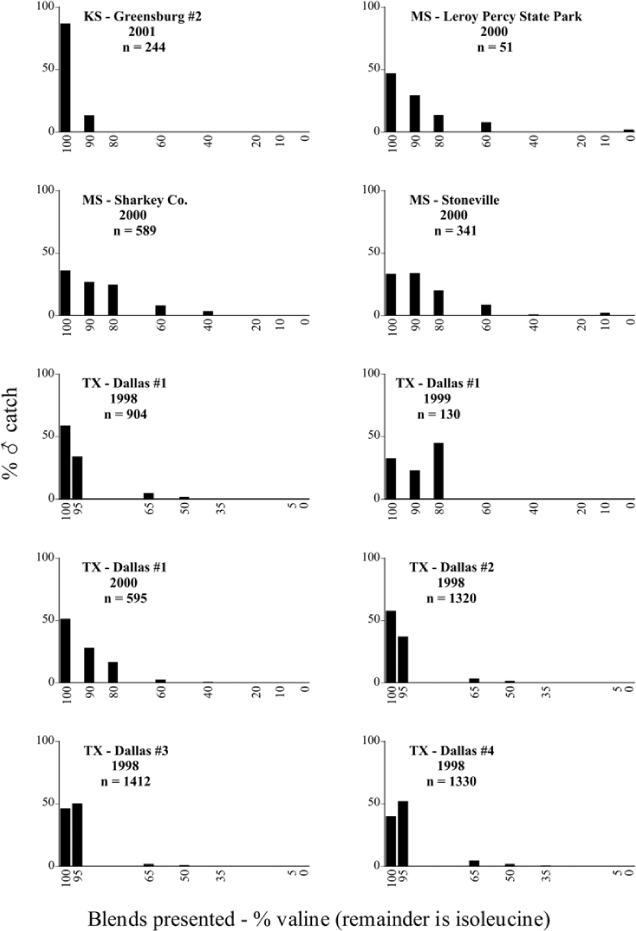
P. congrua ♂ catches

**Figure 72:**
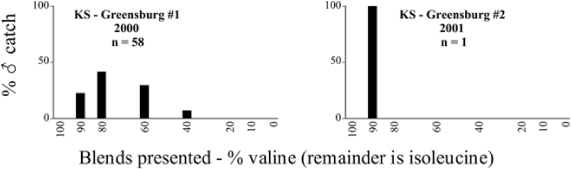
P. corrosa ♂ catches

**Figure 73a:**
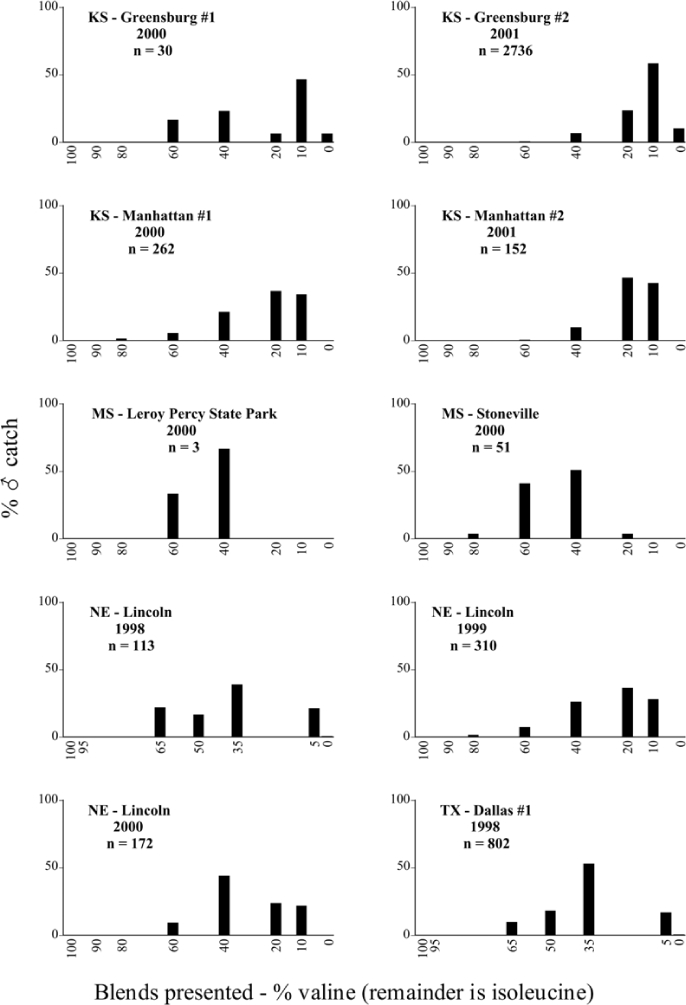
P. crassissima ♂ catches

**Figure 73b:**
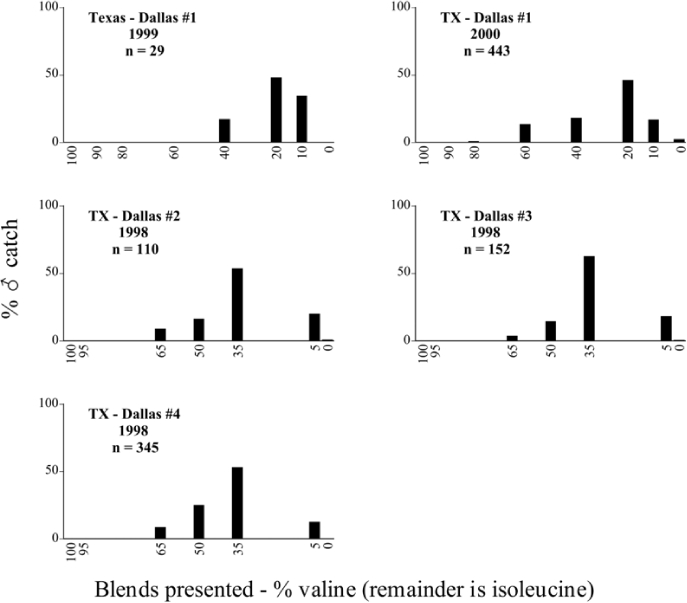
P. crassissima ♂ catches

**Figure 74:**
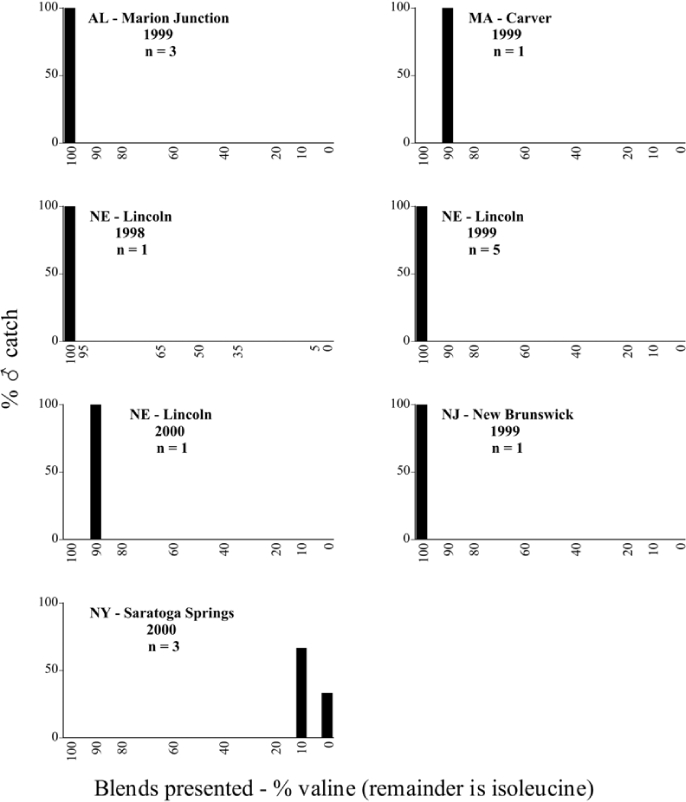
P. crenulata ♂ catches

**Figure 75:**
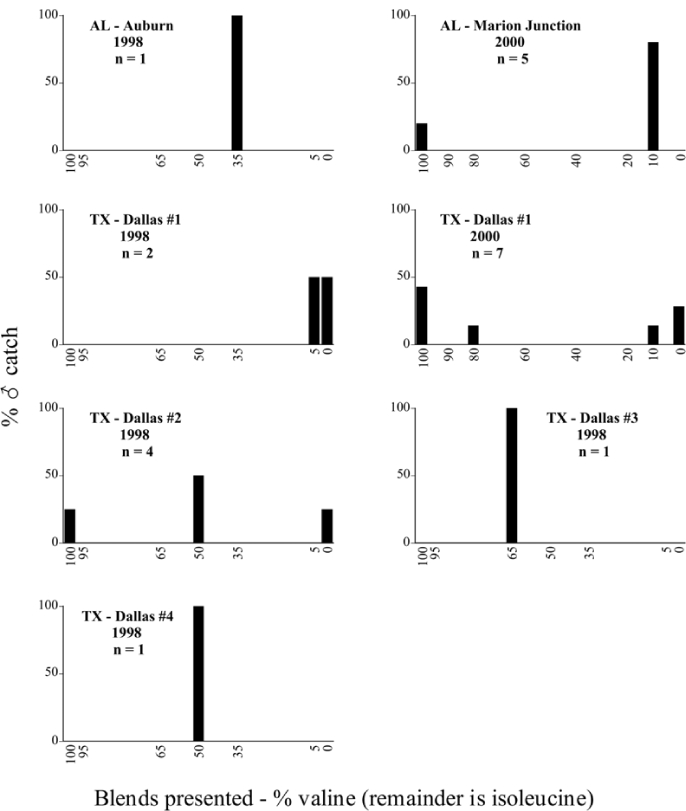
P. crinita ♂ catches

**Figure 76:**
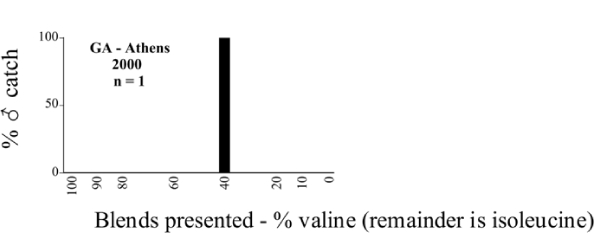
P. curialis ♂ catches

**Figure 77:**
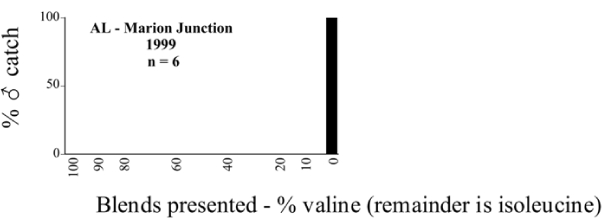
P. davisi ♂ catches

**Figure 78:**
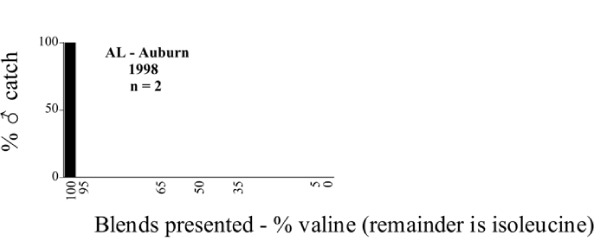
P. diffinis ♂ catches

Copulation does not inevitably lead to fertilization or production of offspring (Eberhard 1996), and similarly, attraction to a congeneric Phyllophaga female does not inevitably lead to copulation. In a study from Costa Rica, Eberhard (1993), reporting on the copulatory behavior of several species of Phyllophaga, states of P. vicina, that “Early in the evening solitary females rested immobile and apparently emitted an attractant, as males arrived in flight from downwind. The pheromone apparently also attracted males of P. valeriana, as on three occasions I saw one or more males of this species hover near a female P. vicina (beetles were captured to verify their species identity). One P. valeriana male landed on the female, then immediately took flight and left, suggesting that a second cue, possibly on the beetle's surface, was used to distinguish species identity. Contact pheromones may be used in the melolonthine genus Macrodactylus (Eberhard 1992).” These observations indicate that more studies are needed to clarify the role of morphology and/or contact pheromones in close-range mate recognition in Phyllophaga. Eberhard (1993) reports that “secondary sexual modifications of the sculpturing of the front legs, the ventral bristles, and the overall leg length of male Macrodactylus may function as courtship devices prior to and during copulation”. Although the Phyllophaga are renowned for their extravagant and often asymmetric genitalic morphology, male hind tibial spurs also often assume unique configurations that provide excellent taxonomic characters for species assignments (Luginbill and Painter 1953; Woodruff and Beck 1989; Woodruff and Sanderson 2004). The role tibial spurs play in close-range mate recognition or copulatory courtship in the Phyllophaga is unclear. Males of some Phyllophaga species also have extensive ventral bristles or possess unique morphological characters on the venter, similar to Macrodactylus (P.S. Robbins, personal observation).

### Intraspecific variation in male response: P. anxia

The consistency of intra-specific male responses over space and time that was discussed earlier contrasts with the extensive variation noted in the male-response curves of P. anxia to the various blends of L-valine methyl ester/L-isoleucine methyl ester sex attractants (Figures 66a, 66b, 66c, 66d, and 66e). The variations demonstrated in the male-response profiles of P. anxia are of four general forms:

- Those profiles from locations where the males flew primarily to L-valine methyl ester alone (Figure 134 and Figures 66a, 66b, 66c, 66d, and 66e including CT Vernon, 1999; NY Bellona, 1999 and 2000; NY Saratoga Springs, 1999 and 2000; NY Warwick, 1999 and 2000; NY Waterloo #1, 1998 NY Waterloo #1, 1999 and 2000; NY Waterloo #2, 1998; ON Woodlawn, 1999 and 2000; PA State College, 1999 and 2000, WI Babcock, 1999 and 2000)
- Those profiles from locations where the males primarily flew to L-isoleucine methyl ester alone (Figure 135 and Figures 66a, 66b, 66c, 66d, and 66e including MA Amherst, 1999 and 2000; NH Madbury, 1999 and 2000).
- Those profiles from locations where males flew to L-valine methyl ester alone or L-isoleucine methyl ester alone on the same night in the same place (Figure 136 and Figures 66a, 66b, 66c, 66d, and 66e, ME Lincolnville Center, 1999 and 2000; NY Franklinville, 1999; VT Burlington, 1999 and 2000).
- Those profiles from locations where the males were mainly captured in traps baited with blends of L-valine methyl ester and L-isoleucine methyl ester (Figure 137 and Figures 66a, 66b, 66c, 66d, and 66e, MA Carver, 1996 Figure 137 and Figures 66a, 66b, 66c, 66d, and 66e, MA Carver, 1998 Figure 137 and Figures 66a, 66b, 66c, 66d, and 66e, MA Carver, 1999, and 2000; MA Lakeville, 1999 and 2000; MA Plympton #1, 1996; MA Plympton #2, 1996; RI Kingston, 1999 and 2000).

All the P. anxia males from Figures 134 to 137, as well as all other P. anxia males in Figures 66a, 66b, 66c, 66d, and 66e are of the northern genitalic form (Luginbill and Painter 1953; Woodruff and Beck 1989). Male P. anxia of the southern genitalic form (Luginbill and Painter 1953; Woodruff and Beck 1989) were captured exclusively with L-isoleucine methyl ester alone (Figure 67). They were captured in both a smaller number of locations (8 vs.25) and in much smaller numbers (20 vs. 20,640) (Table 2a) than P. anxia males of the northern genitalic form. Unpublished data from studies conducted at the Franklinville, NY, site suggest that L-isoleucine methyl ester responding P. anxia males (both northern and southern genitalic forms) are more sensitive to the presence of L-valine methyl ester than are L-valine methyl ester responding males to the presence of L-isoleucine methyl ester. Male captures in the L-isoleucine methyl ester-baited traps may have been suppressed by the presence of the L-valine methyl ester at the trap sites.

The manner in which the different forms of the P. anxia male sex pheromone response profiles are distributed across North America reveals important information. The five locations yielding response profiles (10 site-years) from P. anxia males that were captured in blends of both L-valine methyl ester and L-isoleucine methyl ester are found only in southeast Massachusetts and Rhode Island (see Figures 66a, 66b, 66c, 66d, and 66e including all the MA Carver, MA Lakeville, MA Plympton #1, MA Plympton #2 and RI Kingston sites). Surrounding these five locations are trapping sites to the west (as far west as Wisconsin) and to the north (as far north as the provinces of Nova Scotia and Ontario) that represent those populations of P. anxia males that responded to only L-valine methyl ester or to only L-isoleucine methyl ester, but did not require a blend of the two for capture (Figure 138). The male response curves generated by the beetle captures at those trapping sites yield a distribution map that is patchy in terms of unequal distributions of the two populations. Some sites harbor only one of the two populations, while other sites hold both, thus generating a bimodal distribution curve.

A detailed examination (including the soft sacs) of male individuals of the L-valine methyl ester responding populations, the L-isoleucine methyl ester responding populations, and the blend responding populations of P. anxia revealed no character that could be used to differentiate the populations. Further work is planned that will use DNA sequence data from both mitochondrial and nuclear genes to generate gene genealogies with which to document genetic relationships within and among the three races across the US. Microsatellite markers will also be used to characterize allele frequencies in natural populations and consequently estimate the extent of gene exchange among different pheromone races where they occur together and between geographically isolated populations of single pheromone races.

### Intra-specific variation in male response: P. fraterna

A second instance of intra-specific variation in male response to blends presented was noted at the State College, Pennsylvania, trapping site in 1999 and 2000. Male individuals of a species determined as P. fraterna were captured by two blend groupings in both years (Tables 80 and 81 and Figures 85a, 85b, and 86). In 1999, 18 males were captured with the 0/100 L-valine methyl ester/L-isoleucine methyl ester blend, whereas 13 males were captured with the L-valine methyl ester/L-isoleucine methyl ester mixtures. In 2000, 19 males were captured with the 0/100 L-valine methyl ester/L-isoleucine methyl ester blend whereas 5 males were captured with the L-valine methyl ester/L-isoleucine methyl ester mixtures. Phyllophaga fraterna from locations other than Pennsylvania were captured exclusively in the blends of L-valine methyl ester/L-isoleucine methyl ester (Figures 85a and 85b).

**Figure 79a:**
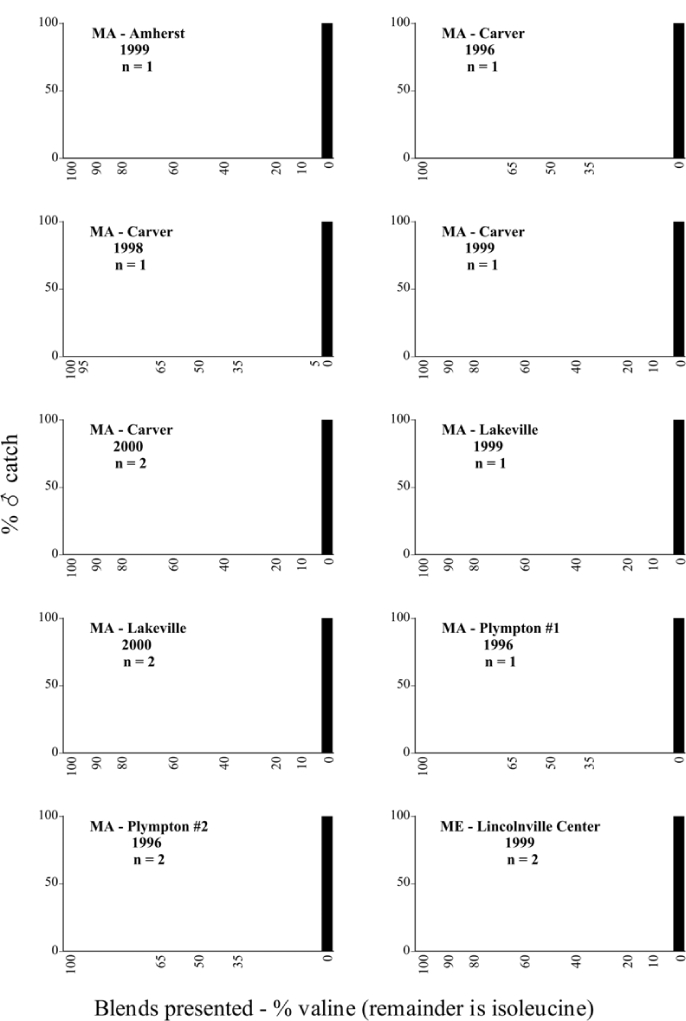
P. drakei ♂ catches

**Figure 79b:**
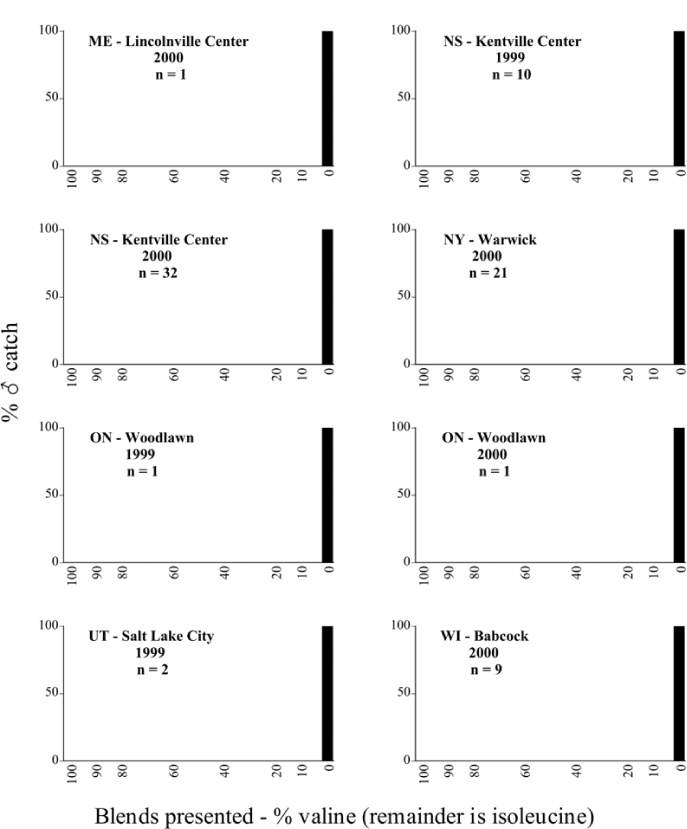
P. drakei ♂ catches

**Figure 80a:**
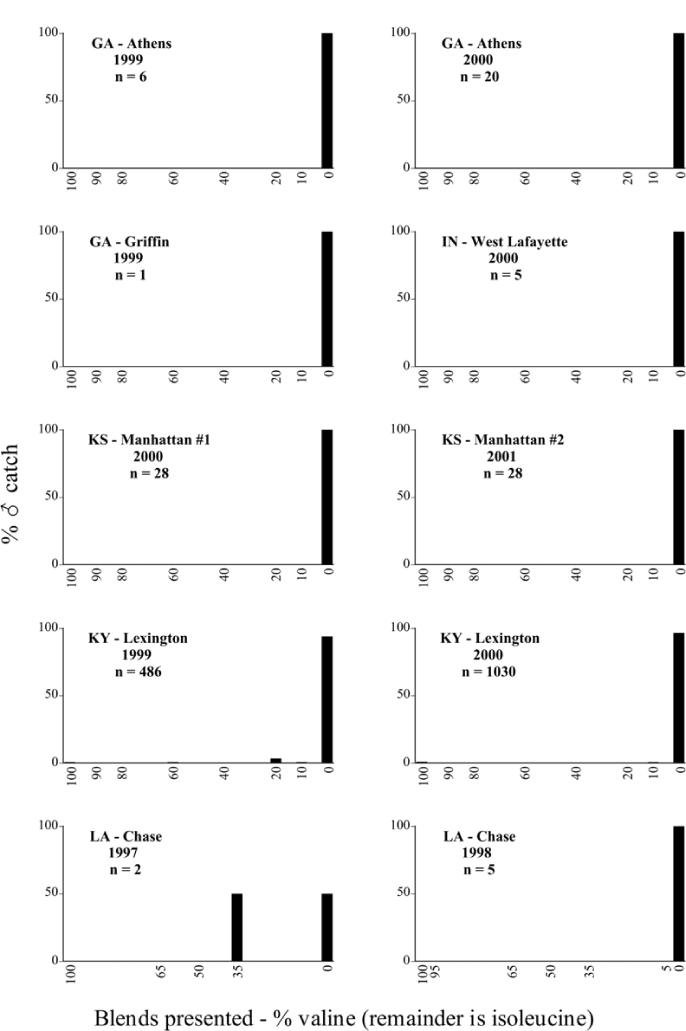
P. ephilida ♂ catches

**Figure 80b:**
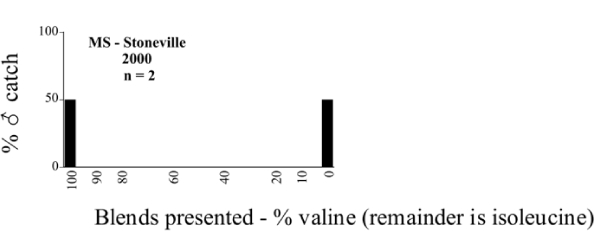
P. ephilida ♂ catches

**Figure 81:**
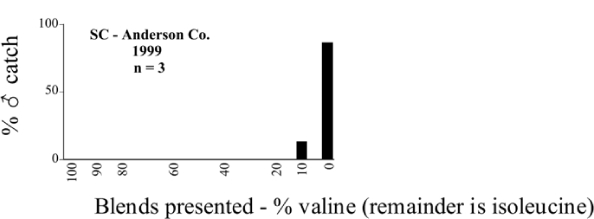
P.fervida ♂ catches

**Figure 82:**
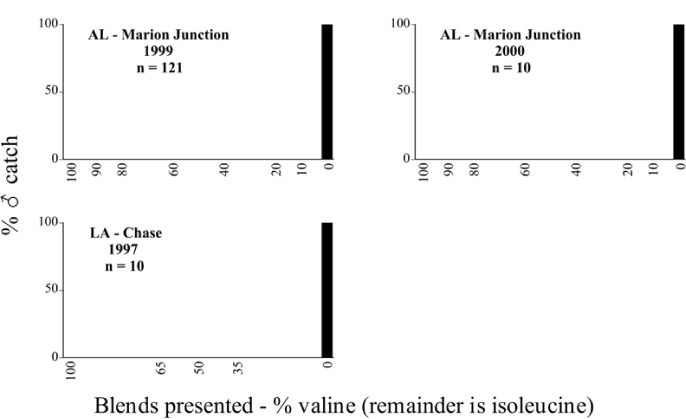
P. forbesi ♂ catches

**Figure 83a:**
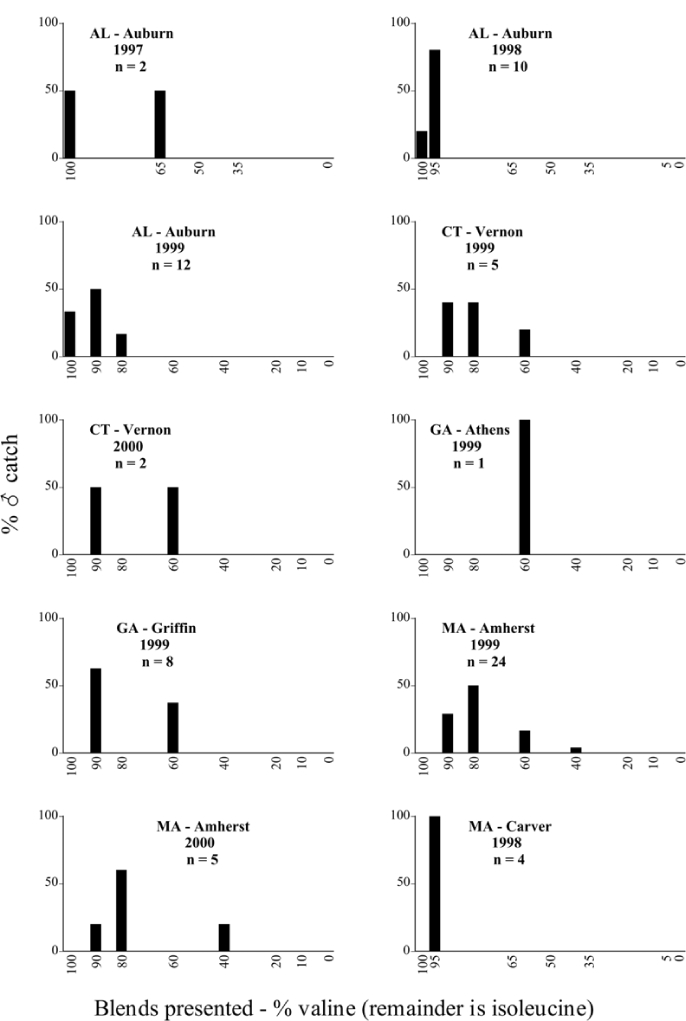
P. forsteri ♂ catches

**Figure 83b:**
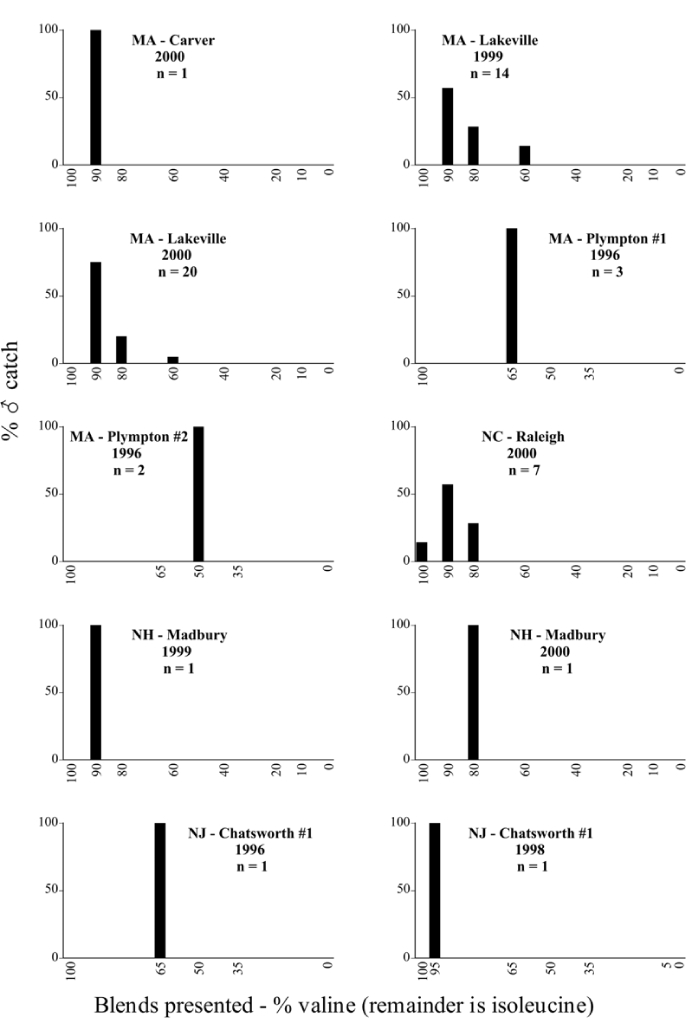
P. forsteri ♂ catches

**Figure 83c:**
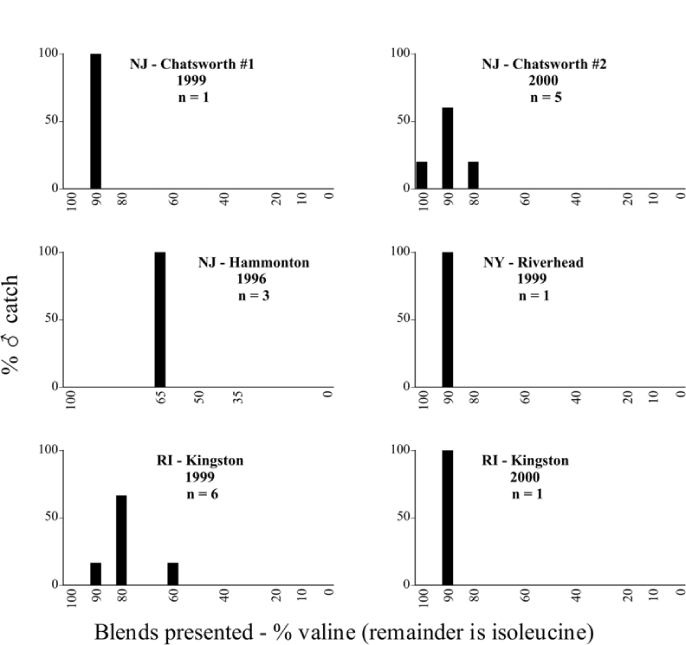
P. forsteri ♂ catches

**Figure 84:**
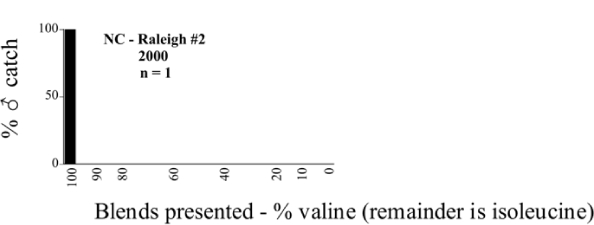
P.foxii ♂ catches

**Figure 85a:**
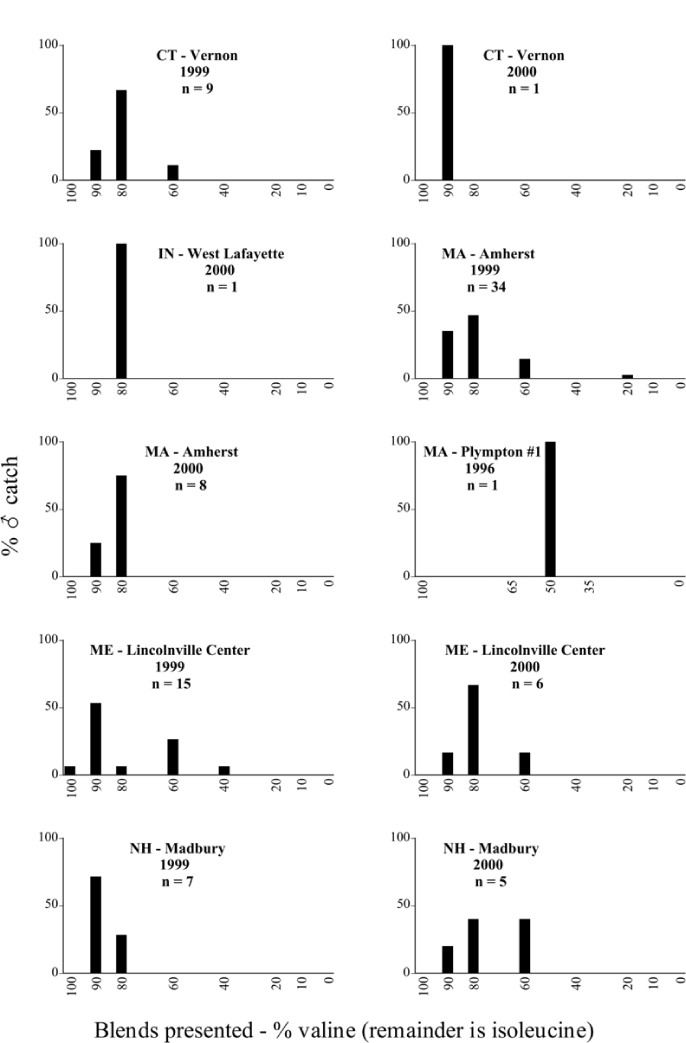
P. fraterna ♂ catches

**Figure 85b:**
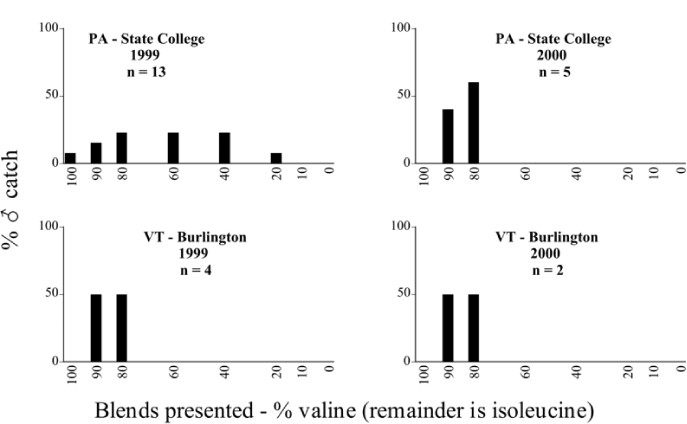
P. fraterna ♂ catches

**Figure 86:**
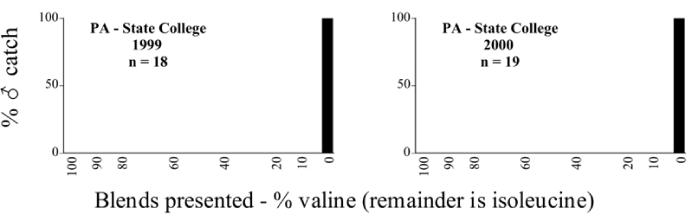
P. fraterna-like ♂ catches

**Figure 87a:**
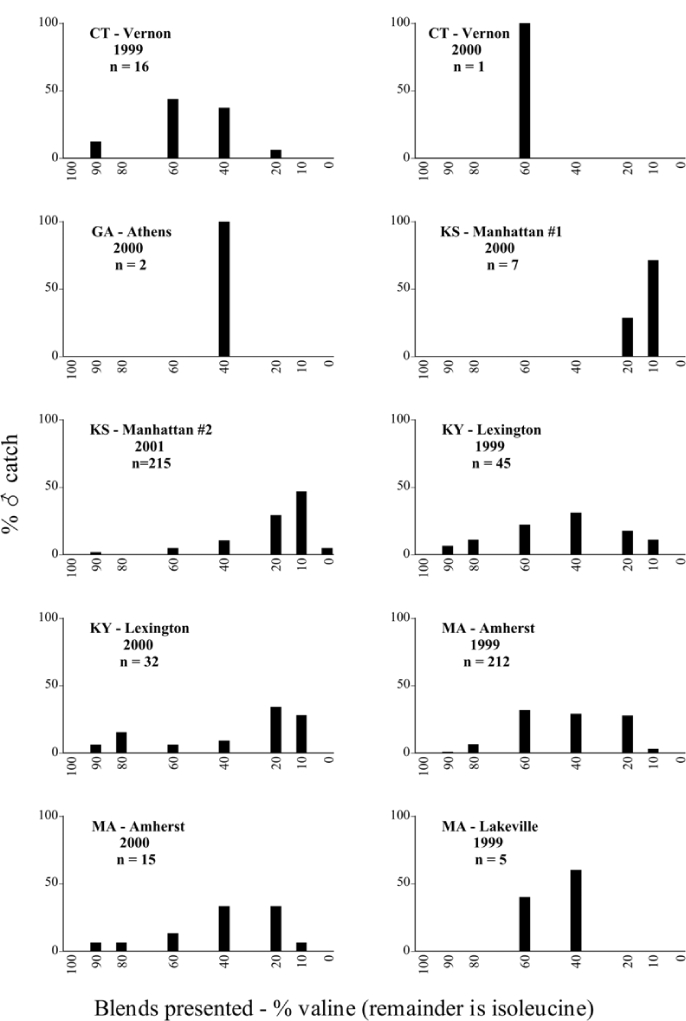
P. fusca ♂ catches

**Figure 87b:**
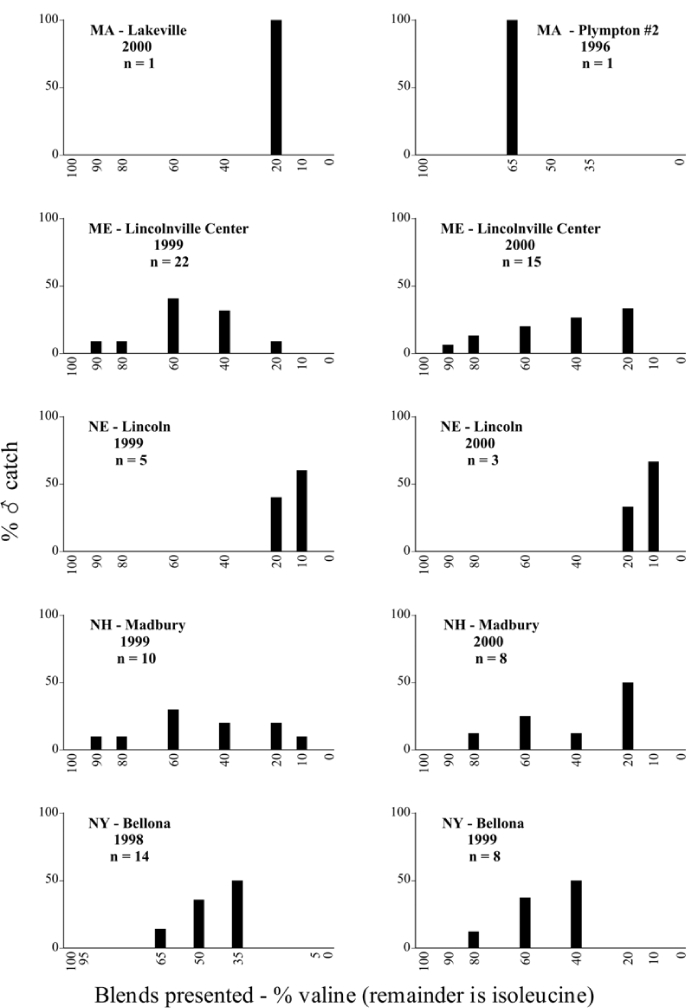
P. fusca ♂ catches

**Figure 87c:**
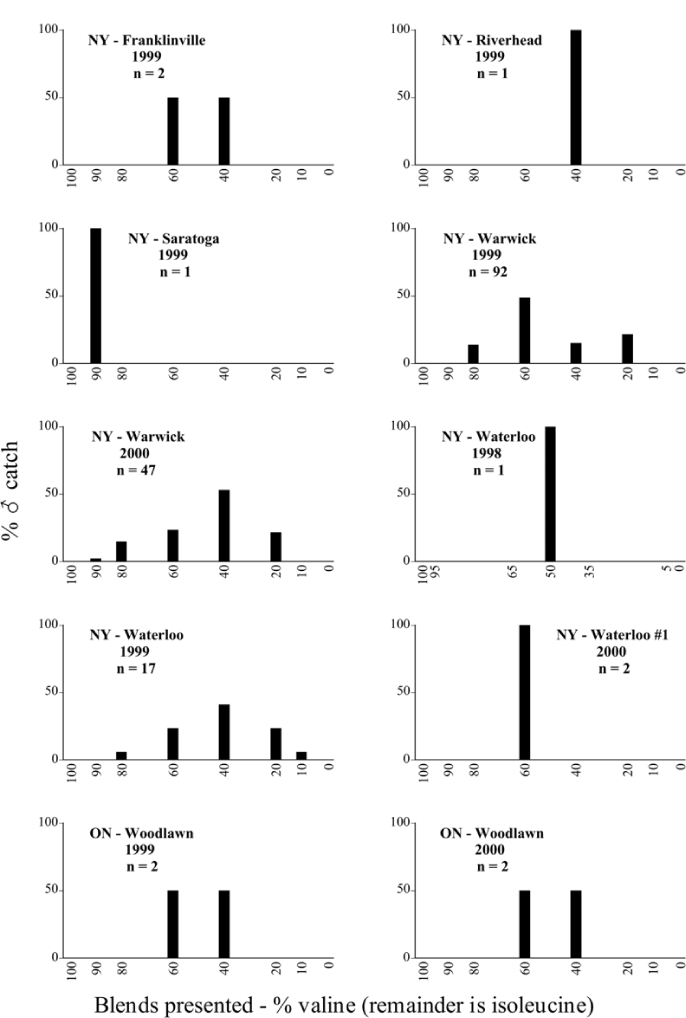
P. fusca ♂ catches

**Figure 87d:**
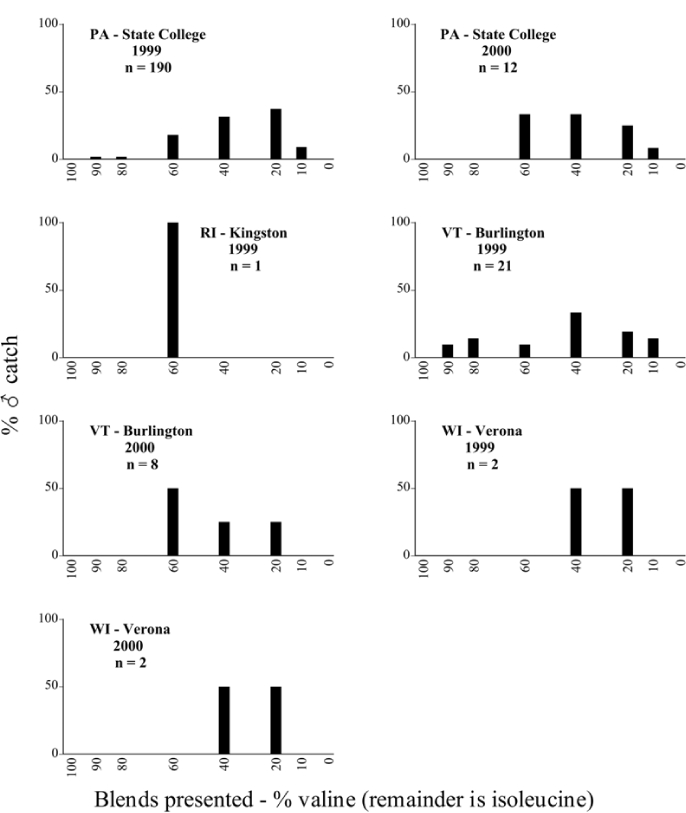
P. fusca ♂ catches

**Figure 88a:**
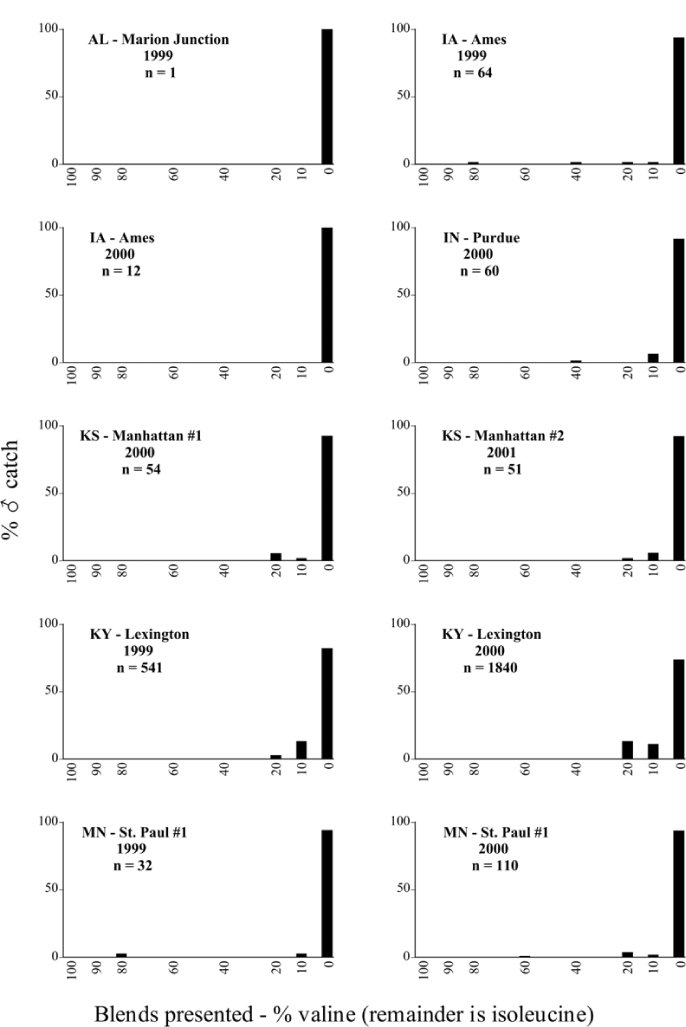
P. futilis ♂ catches

**Figure 88b:**
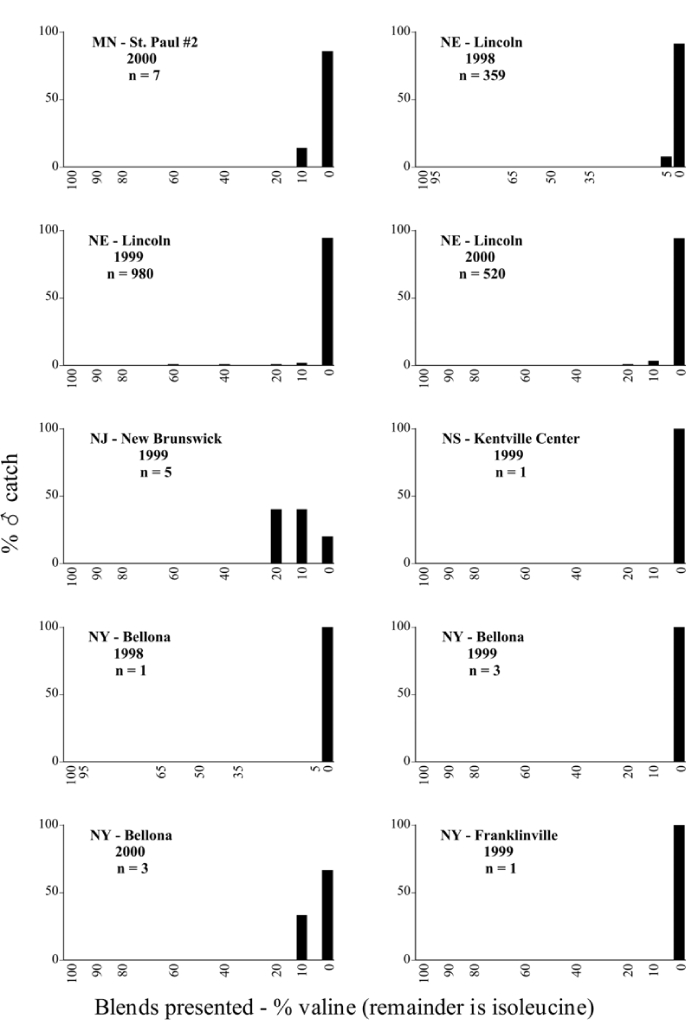
P. futilis ♂ catches

**Figure 88c:**
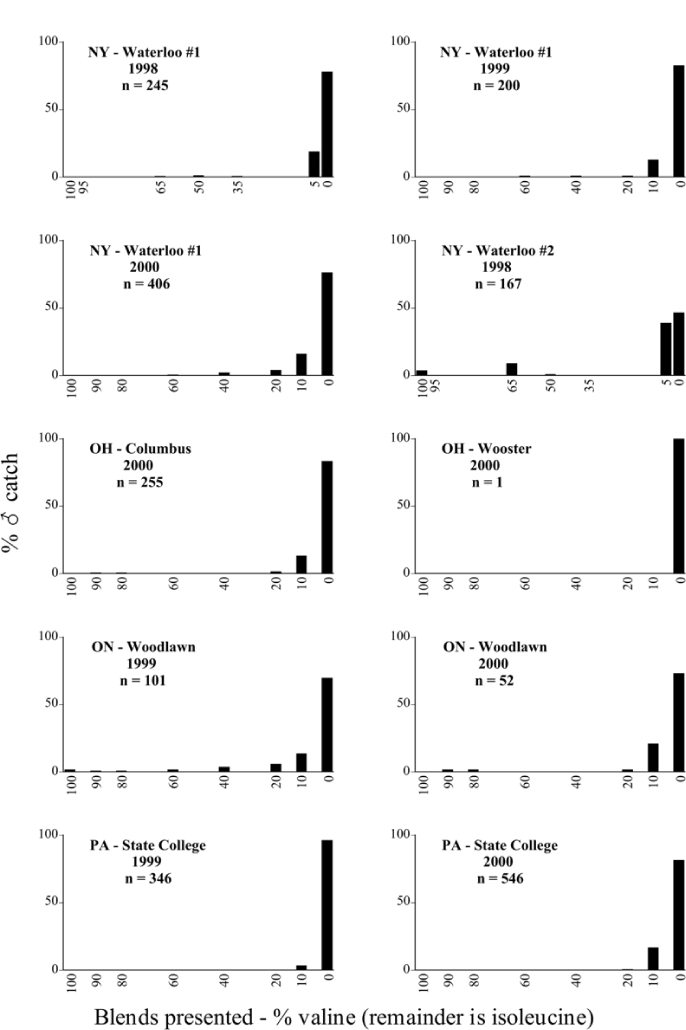
P. futilis ♂ catches

**Figure 88d:**
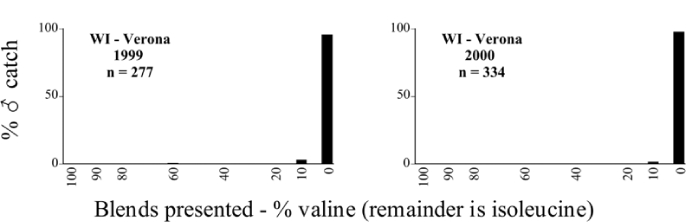
P. futilis ♂ catches

**Figure 89:**
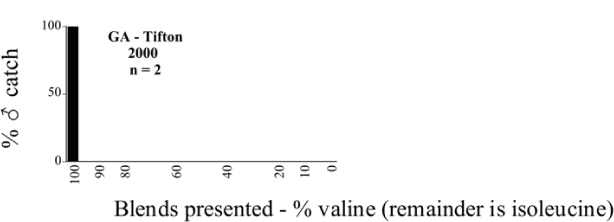
P. (Phytalus) georgiana ♂ catches

**Figure 90:**
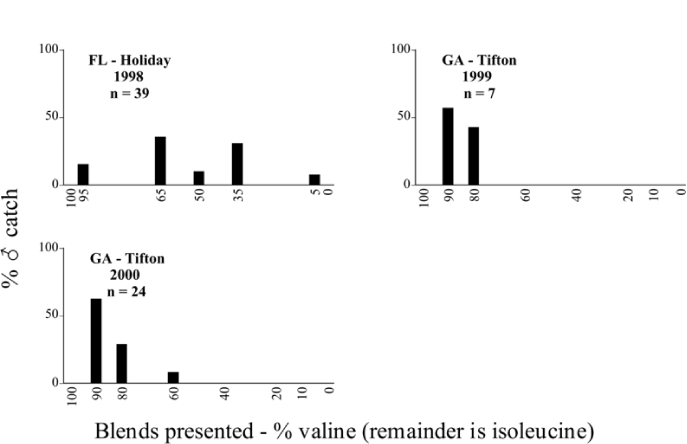
P. glaberrima ♂ catches

**Figure 91:**
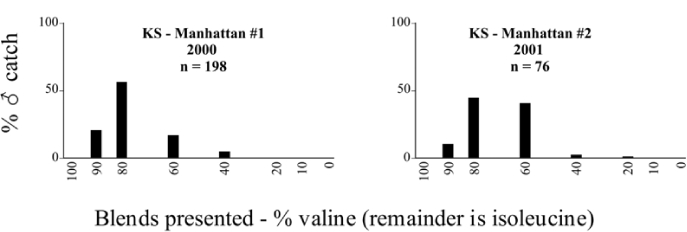
P. glabricula ♂ catches

**Figure 92:**
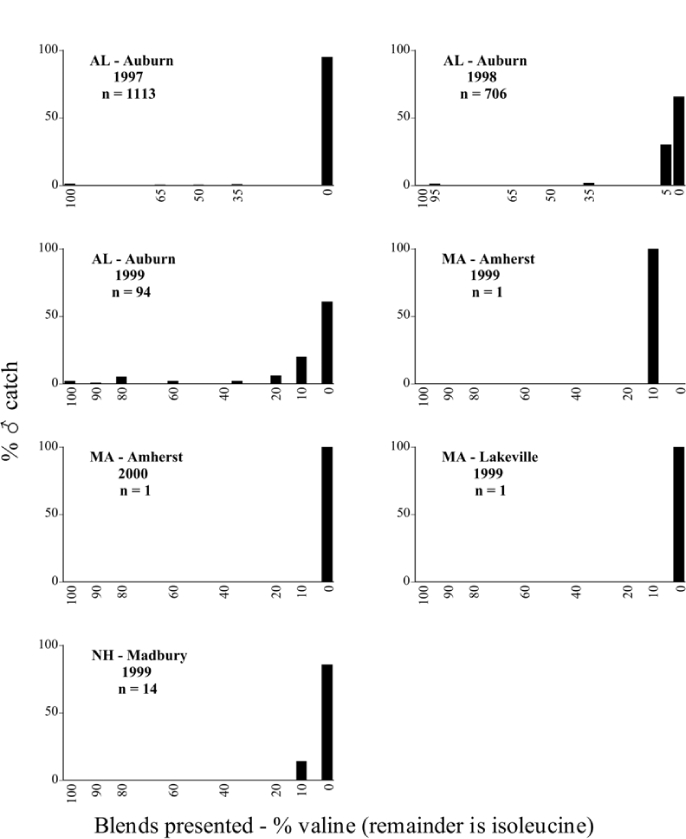
P. gracilis ♂ catches

**Figure 93:**
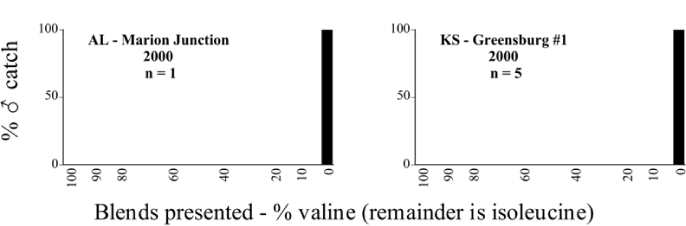
P. gracilis var. angulata ♂ catches

**Figure 94:**
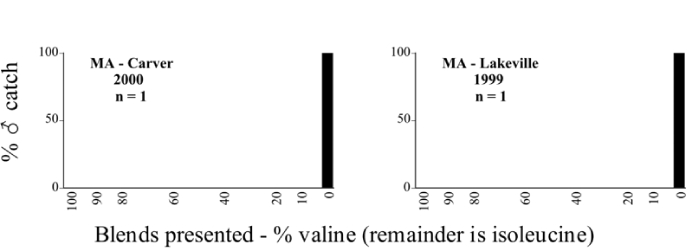
P. hirsuta ♂ catches

**Figure 95:**
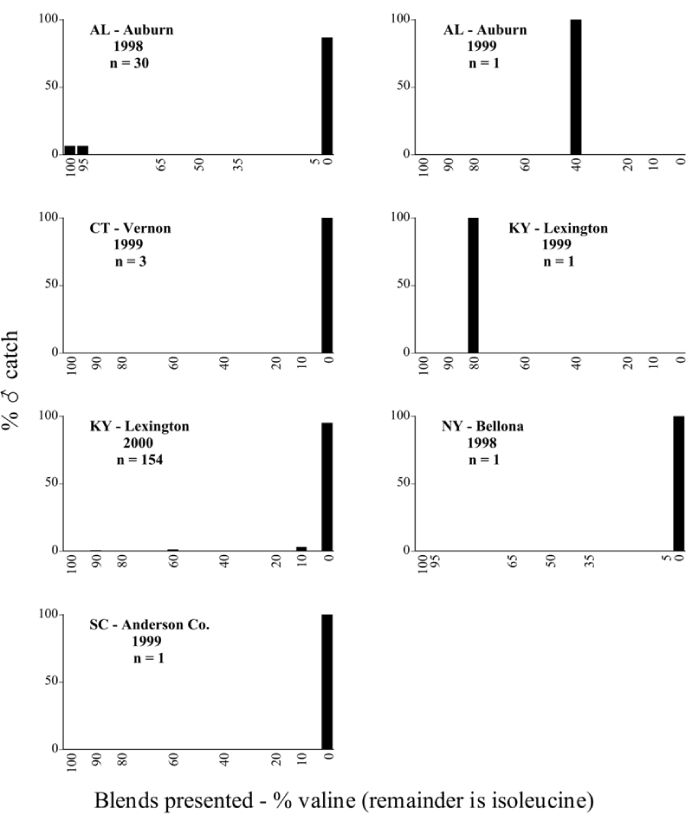
P. hirticula ♂ catches

**Figure 96:**
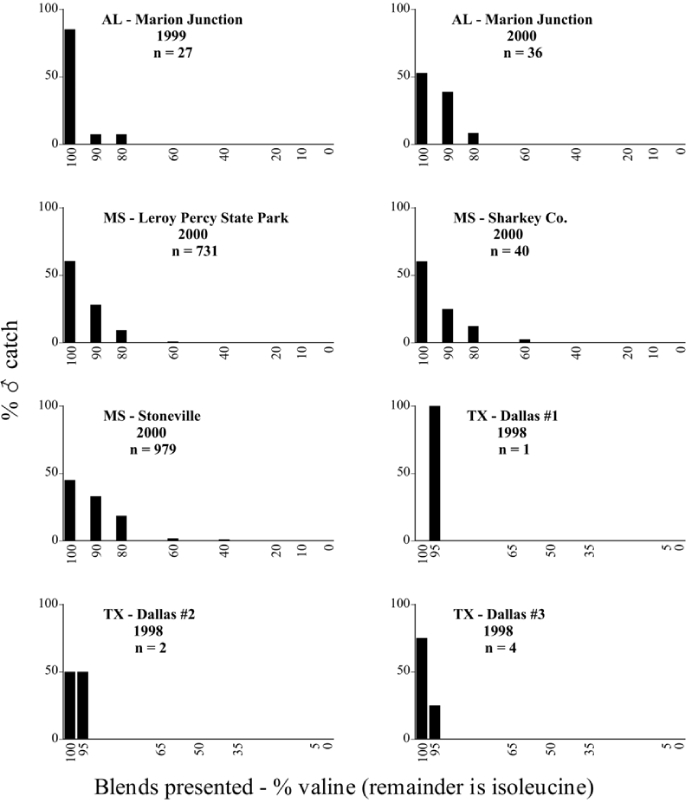
P. hirtiventris ♂ catches

**Figure 97:**
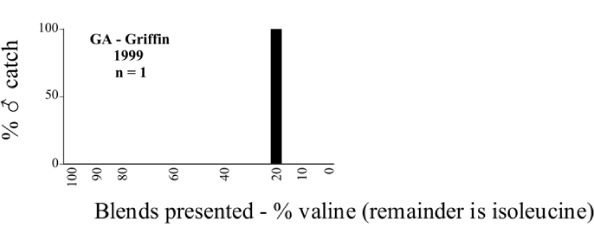
P. ilicis ♂ catches

**Figure 98:**
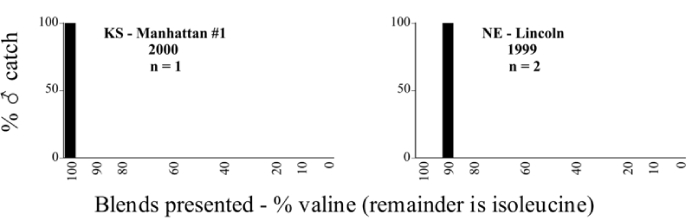
P. implicita ♂ catches

**Figure 99:**
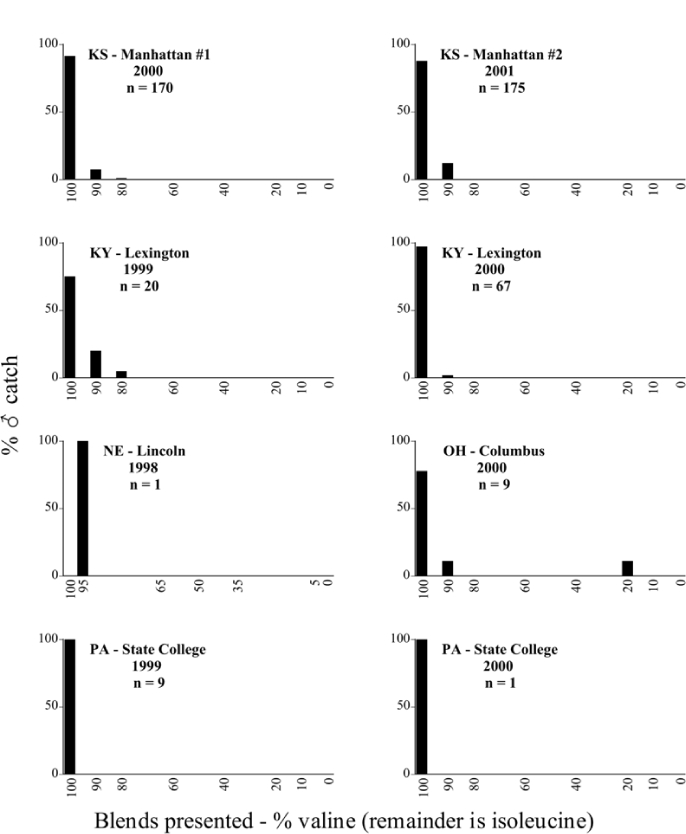
P. inversa ♂ catches

**Figure 100:**
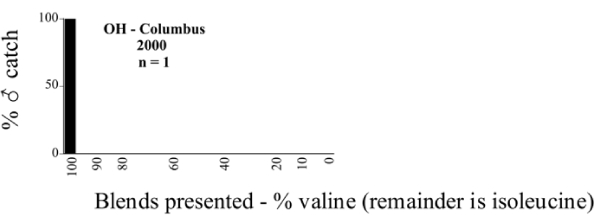
P. kentuckiana ♂ catches

**Figure 101:**
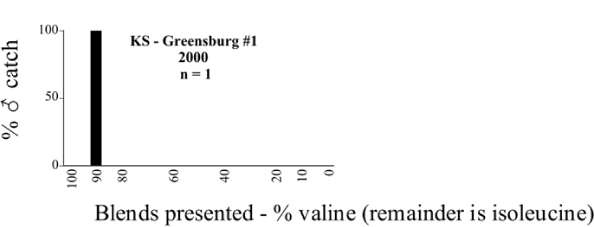
P. lanceolata ♂ catches

**Figure 102:**
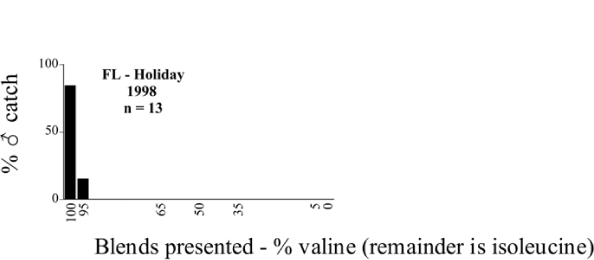
P. latifrons ♂ catches

**Figure 103:**
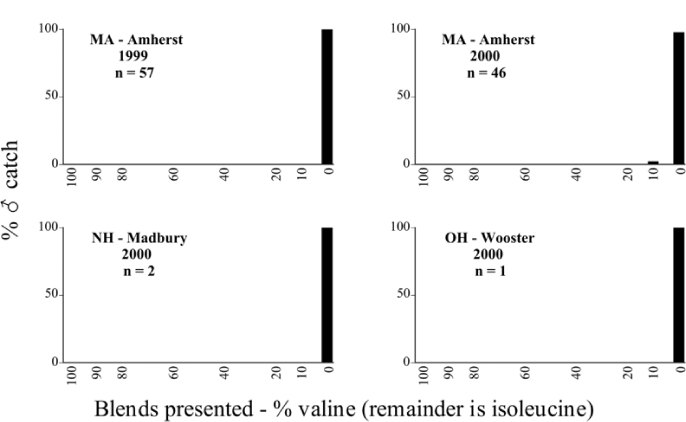
P. longispina ♂ catches

**Figure 104:**
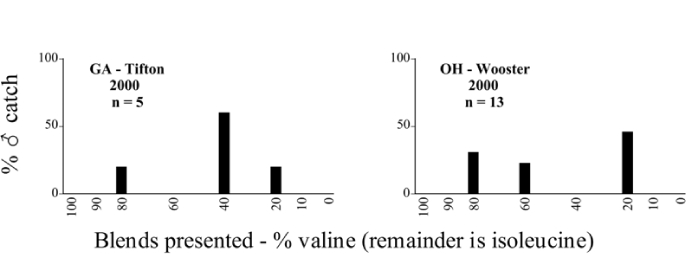
lota ♂ catches

**Figure 105:**
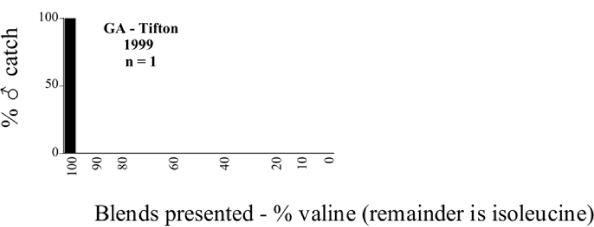
P. luctuosa ♂ catches

**Figure 106:**
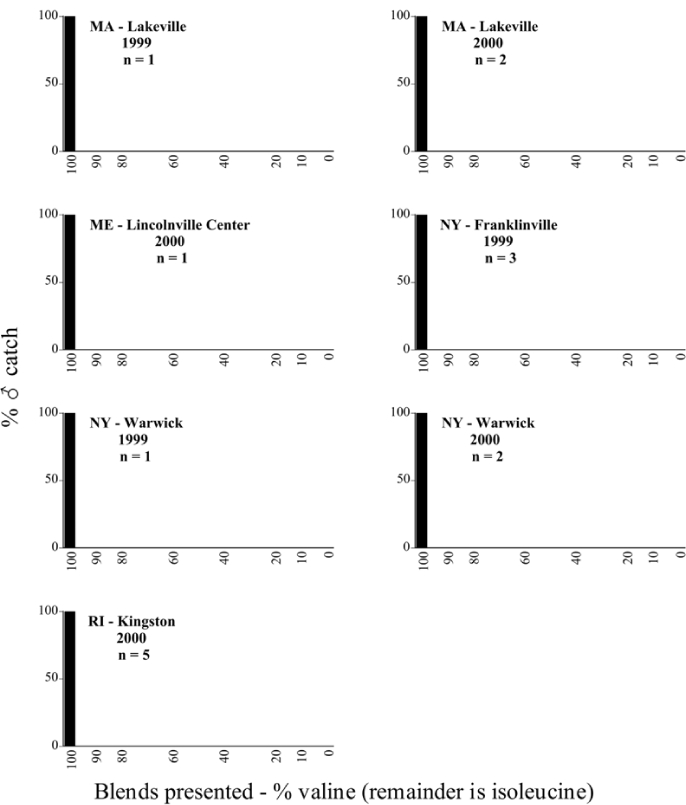
P. marginalis ♂ catches

**Figure 107:**
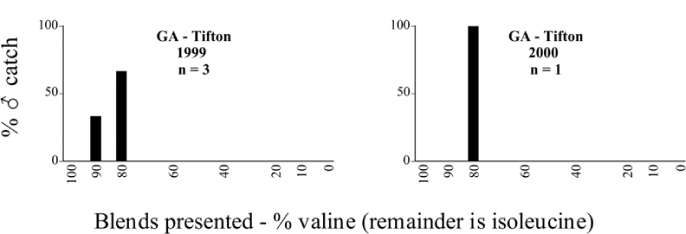
P. mariana ♂ catches

**Figure 108:**
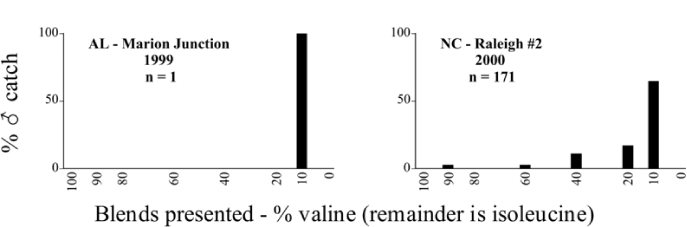
P. micans ♂ catches

**Figure 109:**
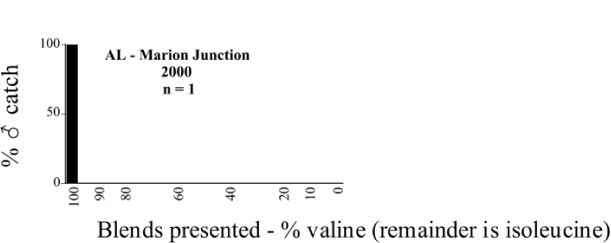
P. (fraterna) mississippiensis ♂ catches

**Figure 110:**
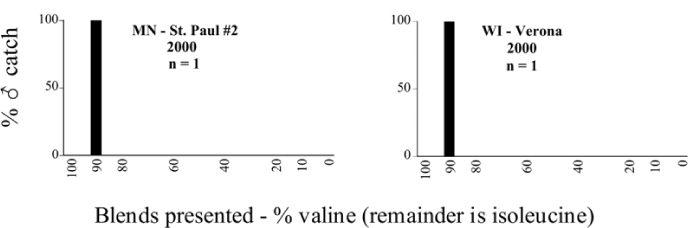
P. nitida ♂ catches

**Figure 111:**
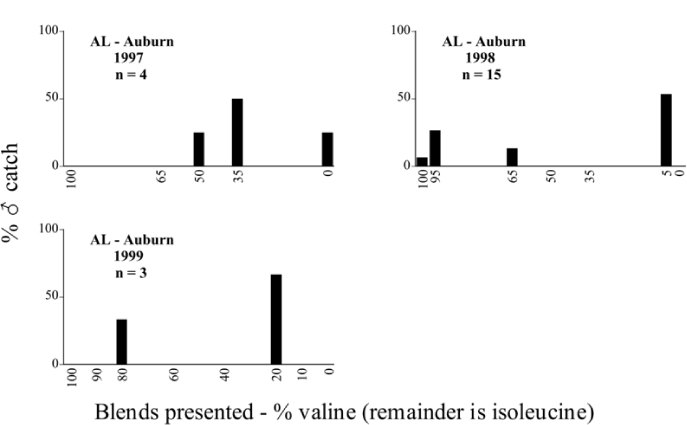
P. obsoleta ♂ catches

**Figure 112:**
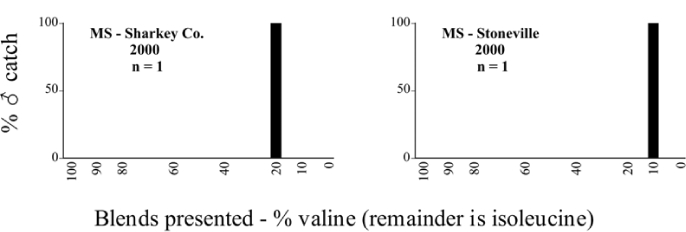
P. perlonga ♂ catches

**Figure 113:**
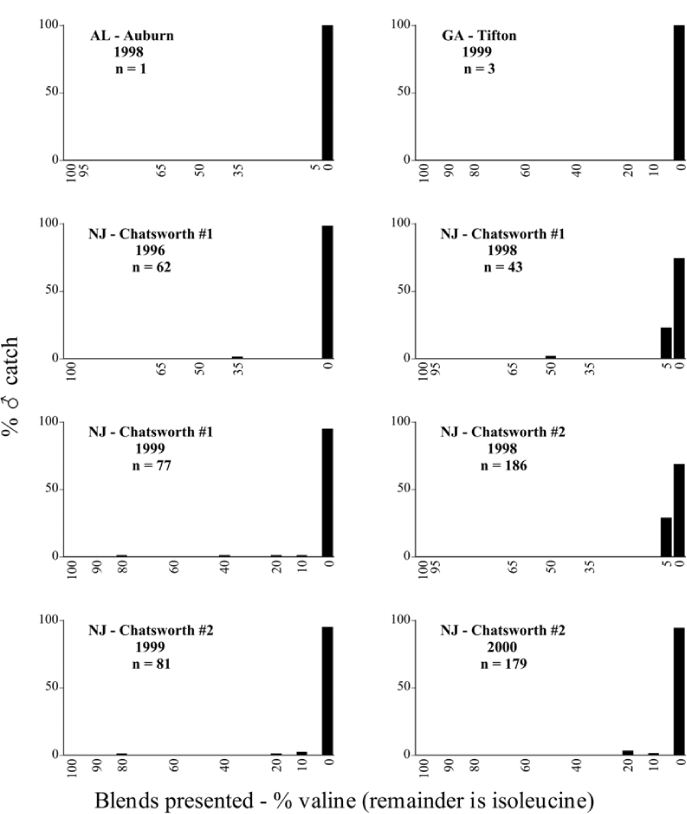
P. postrema ♂ catches

**Figure 114:**
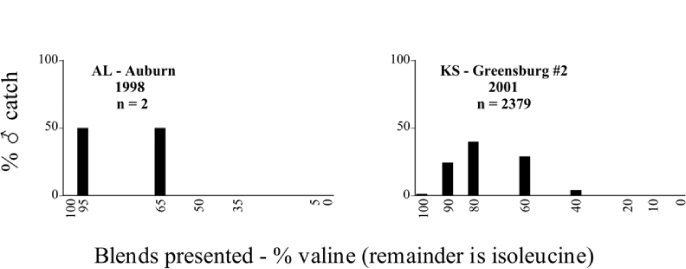
P. praetermissa ♂ catches

**Figure 115:**
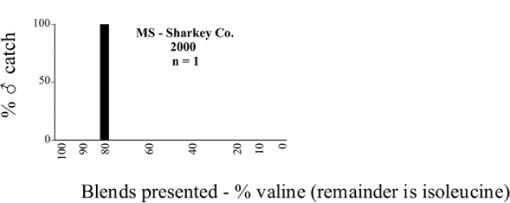
P. profunda ♂ catches

**Figure 116:**
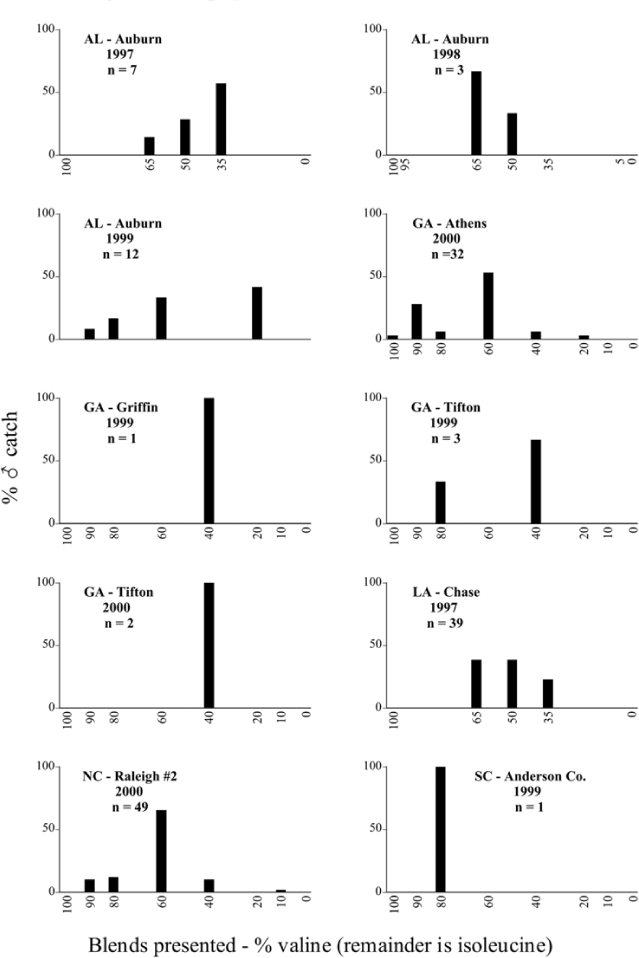
P. quercus ♂ catches

**Figure 117:**
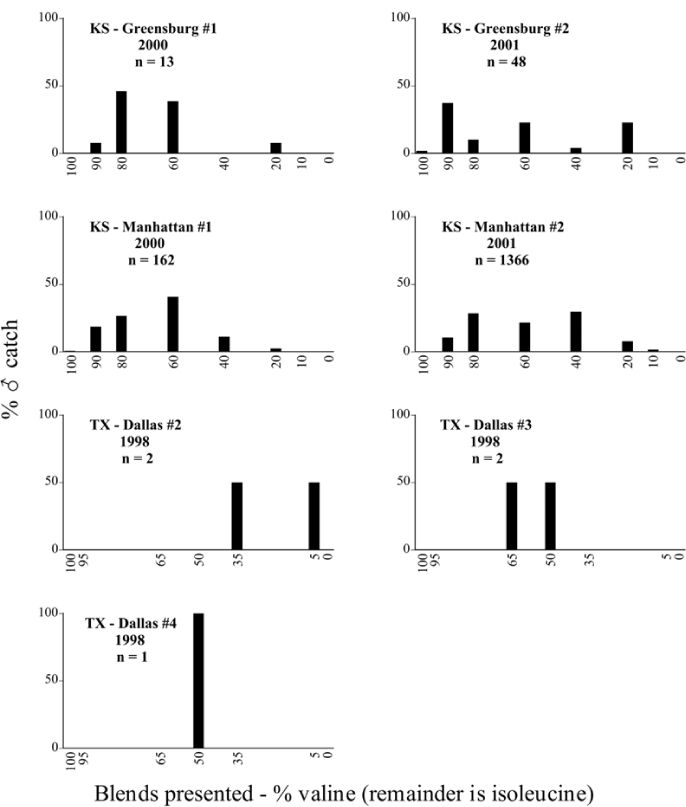
P. rubiginosa ♂ catches

**Figure 118a:**
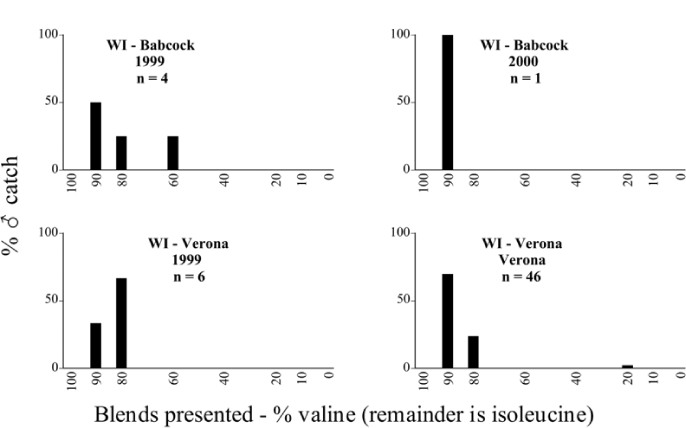
P. rugosa ♂ catches

**Figure 118b:**
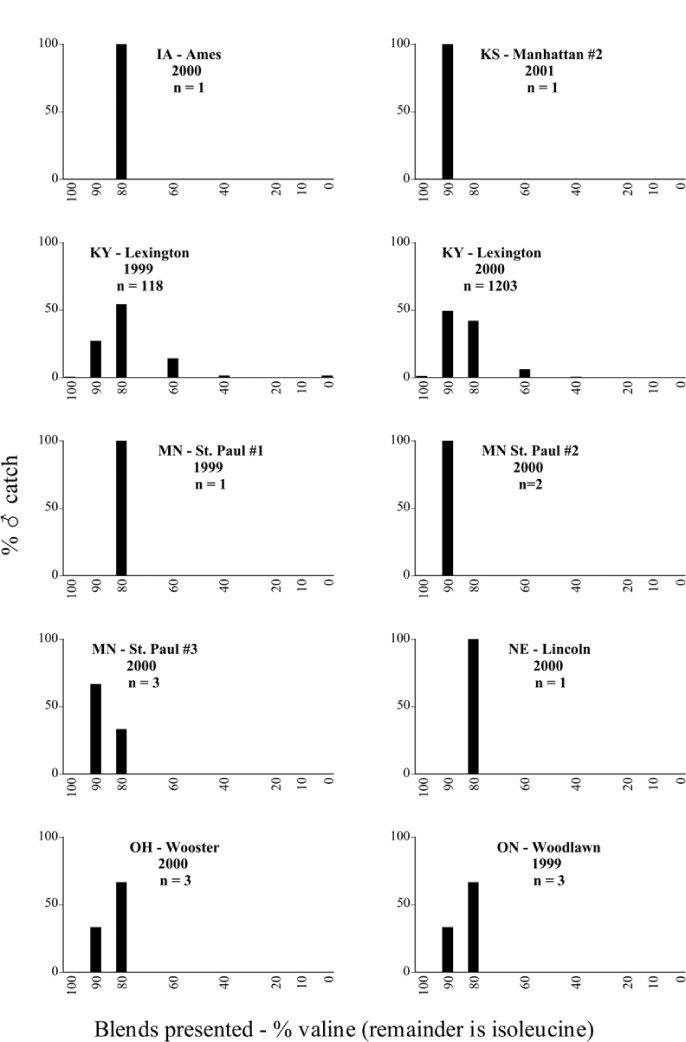
P. rugosa ♂ catches

**Figure 119:**
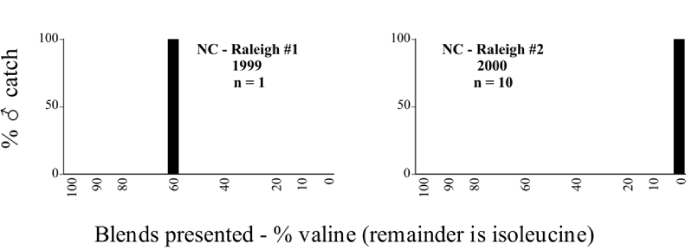
P. soror ♂ catches

**Figure 120:**
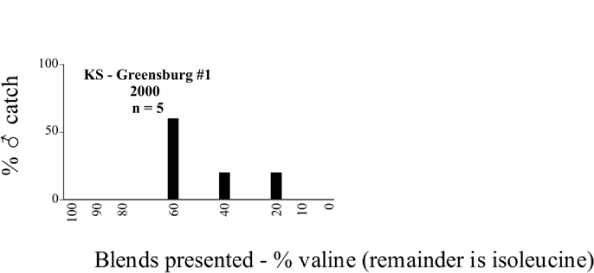
P. submucida ♂ catches

**Figure 121:**
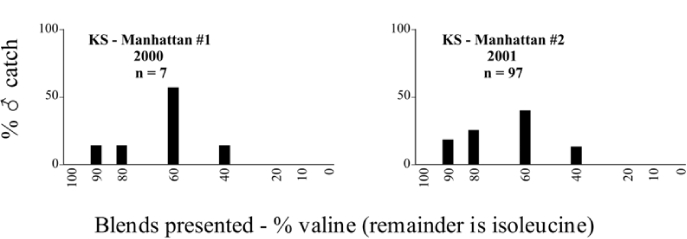
P. sylvatica ♂ catches

**Figure 122:**
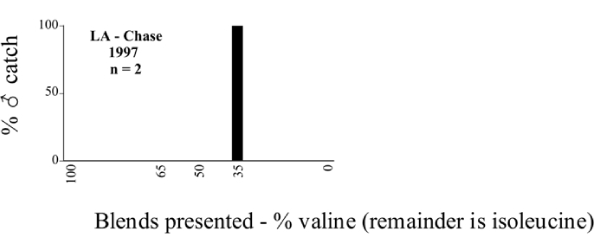
P. taxodii ♂ catches

**Figure 123:**
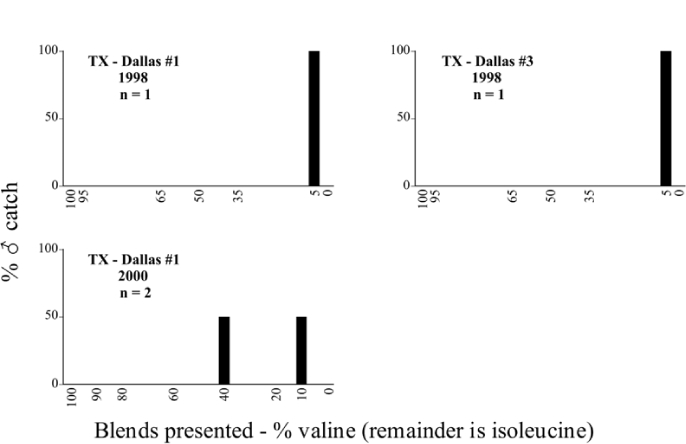
torta ♂ catches

**Figure 124:**
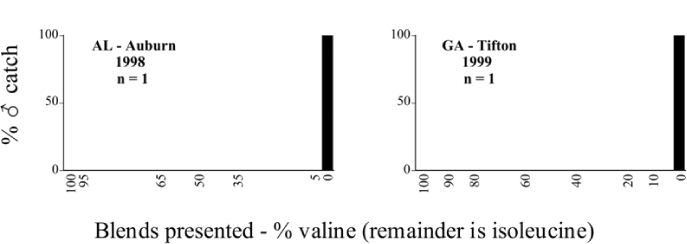
P. ulkei ♂ catches

**Figure 125:**
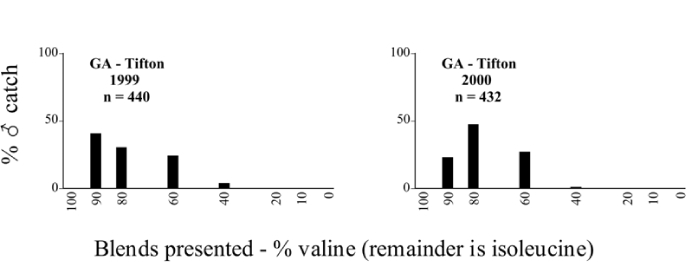
P. uniformis ♂ catches

**Figure 126:**
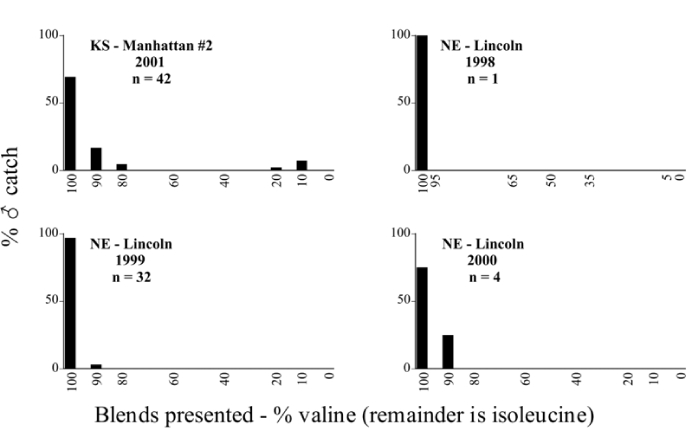
P. vehemens ♂ catches

**Figure 127:**
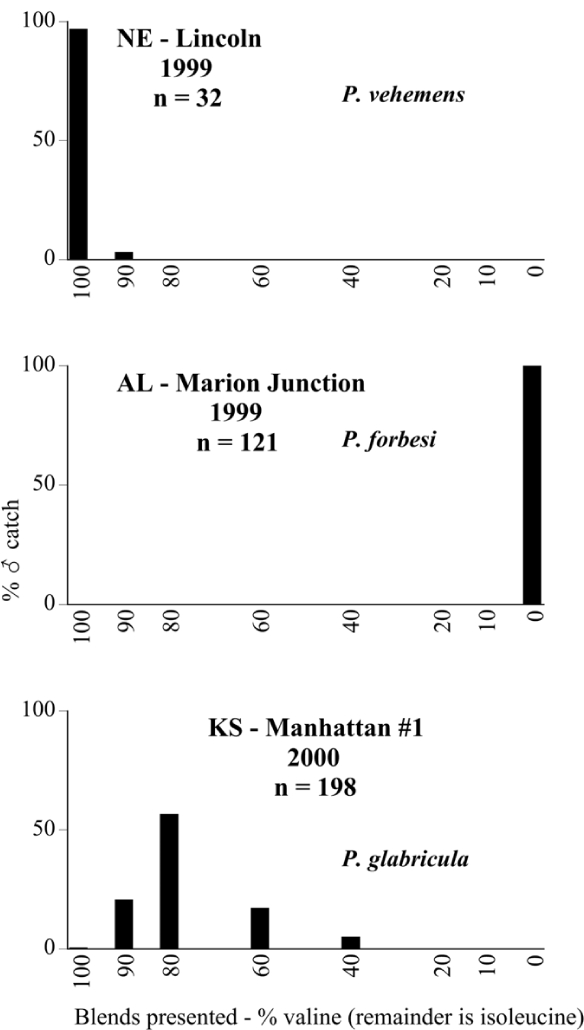
Response curves of ♂ Phyllophaga to sex attractants.

**Figure 128:**
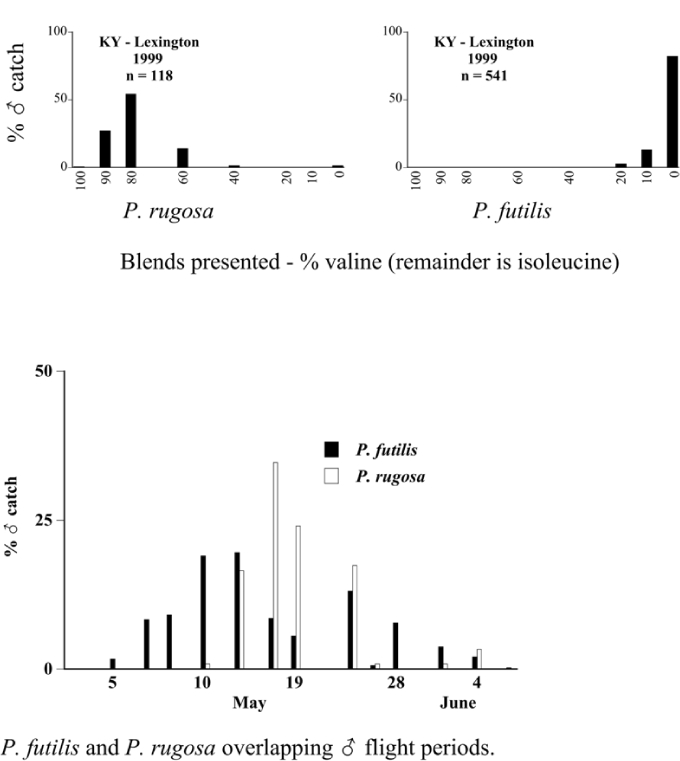
Sympatric Phyllophaga species flying synchronically to different blends of valine/isoleucine. Kentucky, Lexington, 1999.

**Figure 129:**
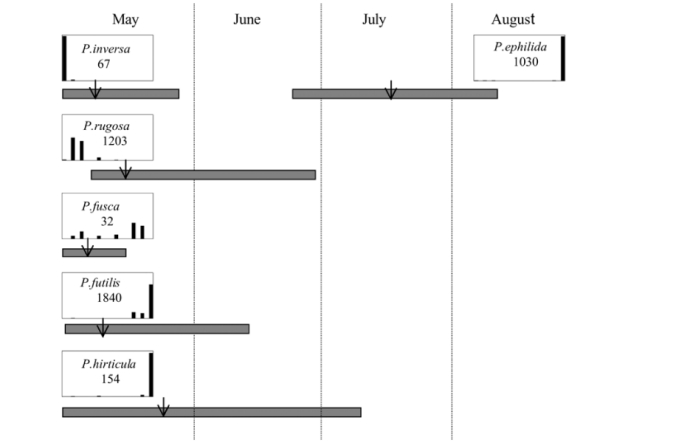
Sympatric Phyllophaga species flying asynchronically to the same pheromone. Nebraska, Lincoln, 1999.

**Figure 130:**
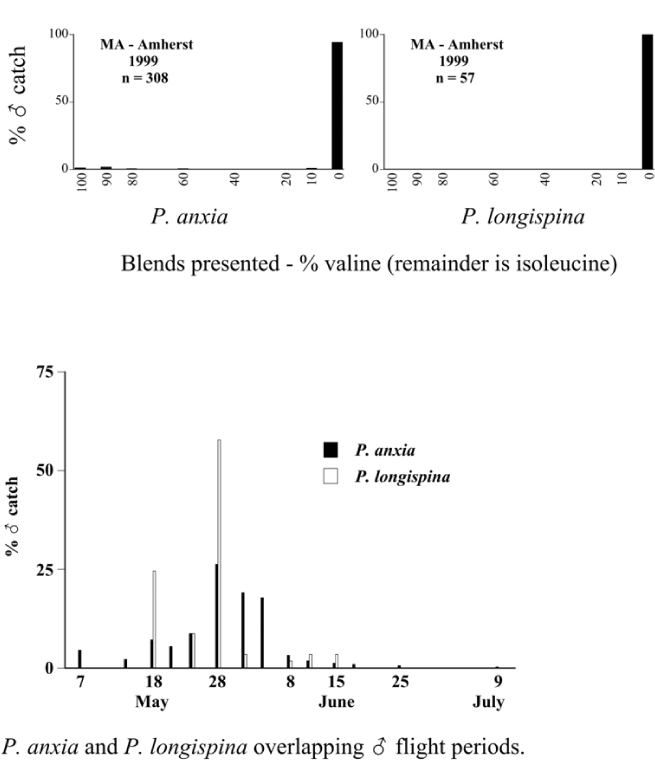
Sympatric Phyllophaga species flying synchronically to the same pheromone. Massachusetts, South Amherst, 1999.

**Figure 131:**
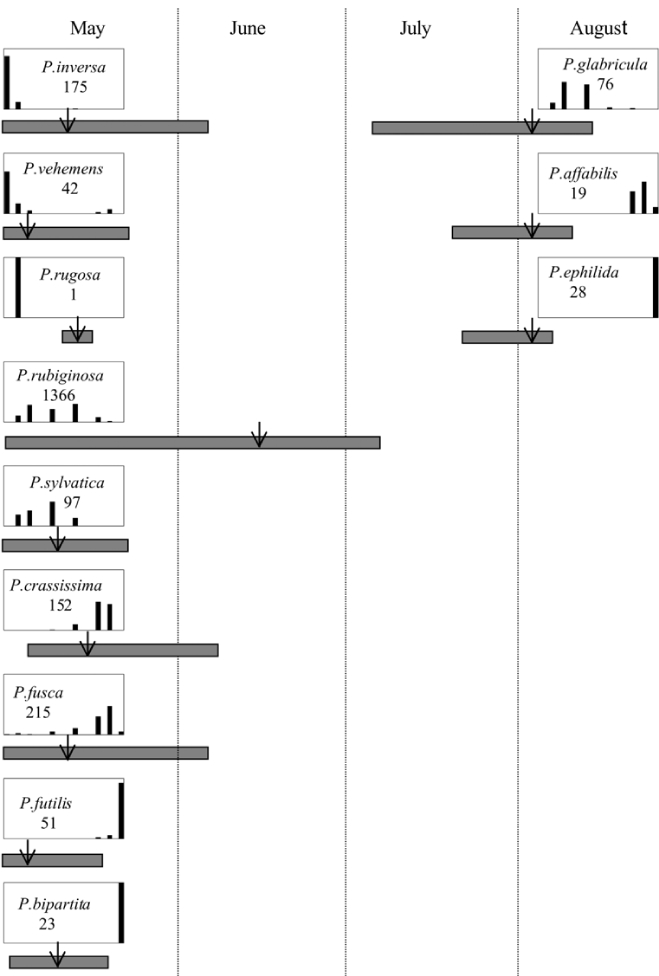
Kansas, Manhattan #2, 2001. Timing and duration of flight and ♂ pheromone response curves. Arrows indicate numerical midpoints of flights.

**Figure 132:**
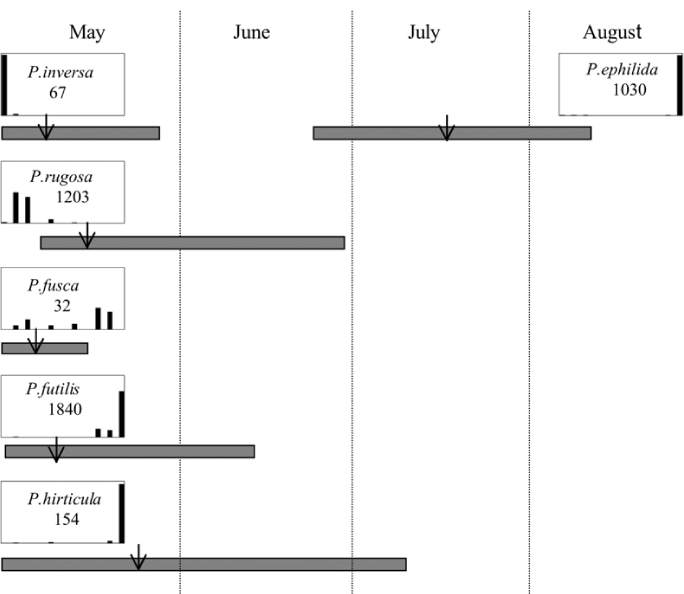
Kentucky, Lexington, 2000. Timing and duration of flight and ♂ pheromone response curves. Arrow indicates numerical midpoint of flight.

**Figure 133:**
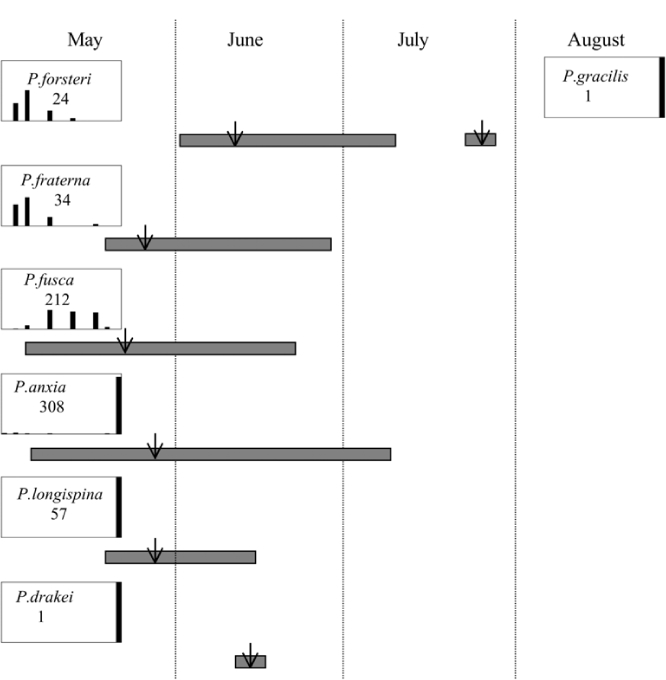
Massachusetts, Amherst, 1999. Timing and duration of flight and ♂ pheromone response curves. Arrow indicates numerical midpoint of flight.

**Figure 134:**
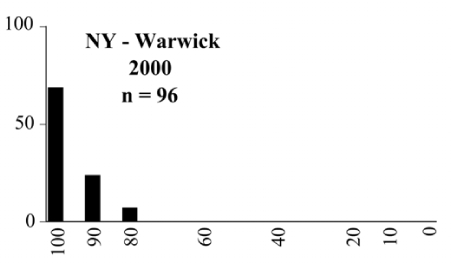
Valine responding ♂ capture curve, P. anxia.

**Figure 135:**
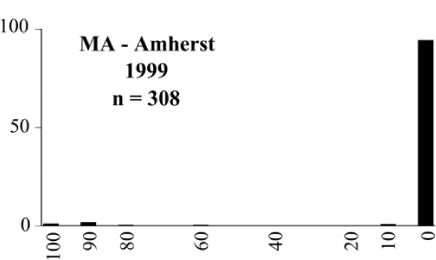
Isoleucine responding ♂ capture curve, P. anxia.

**Figure 136:**
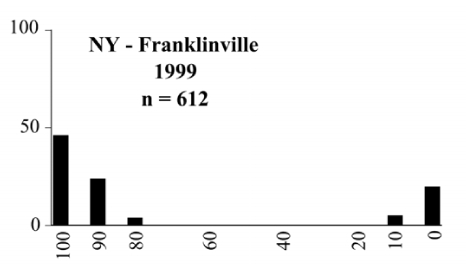
Bimodal ♂ capture curve, P. anxia.

**Figure 137:**
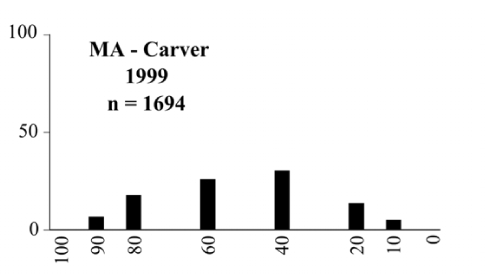
Blend responding ♂ capture curve, P. anxia.

**Figure 138:**
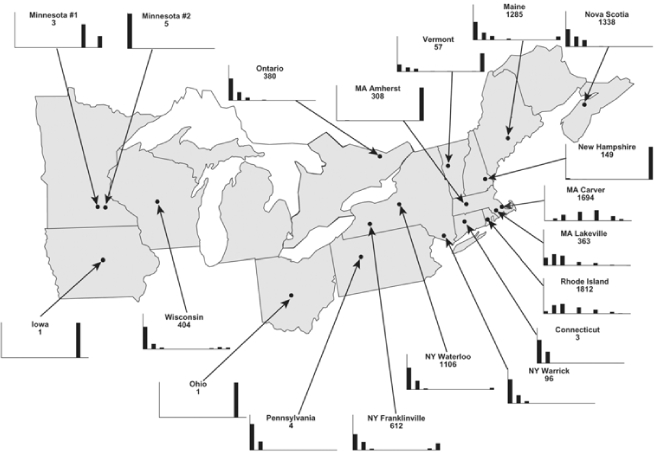
♂ capture curves for Phyllophaga anxia (LeConte), northern genitalic form (n = 20,460. Not all locations shown in this figure.)

**Table 59.**
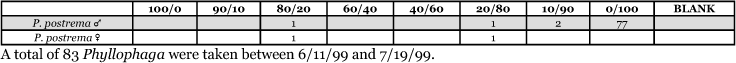
New Jersey, Chatsworth #2 1999 Blends indicate the ratio of the methyl esters of L-valine/L-isoleucine

**Table 60.**
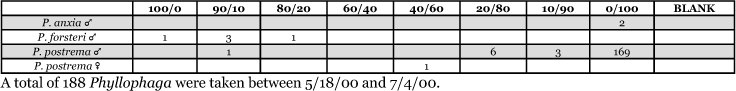
New Jersey, Chatsworth #2 2000 Blends indicate the ratio of the methyl esters of L-valine/L-isoleucine

**Table 61.**
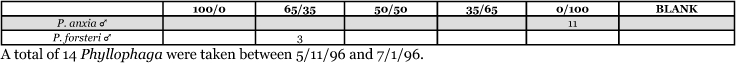
New Jersey, Hammonton 1996 Blends indicate the ratio of the methyl esters of L-valine/L-isoleucine

**Table 62.**
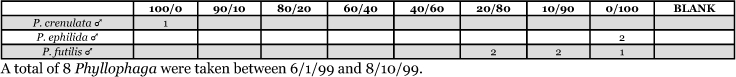
New Jersey, New Brunswick 1999 Blends indicate the ratio of the methyl esters of L-valine/L-isoleucine

**Table 63.**
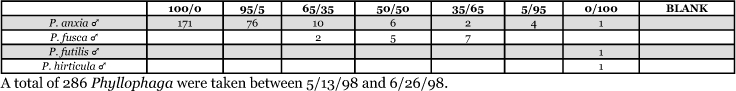
New York, Bellona 1998 Blends indicate the ratio of the methyl esters of L-valine/L-isoleucine

**Table 64.**
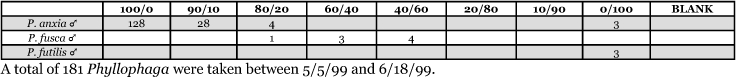
New York, Bellona 1999 Blends indicate the ratio of the methyl esters of L-valine/L-isoleucine

**Table 65.**
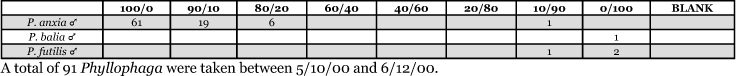
New York, Bellona 2000 Blends indicate the ratio of the methyl esters of L-valine/L-isoleucine

**Table 66.**
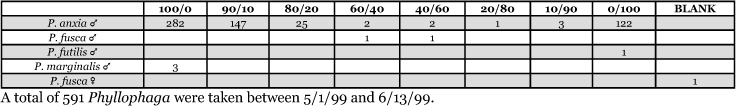
New York, Franklinville 1999 Blends indicate the ratio of the methyl esters of L-valine/L-isoleucine

**Table 67.**
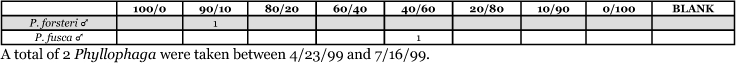
New York, Riverhead 1999 Blends indicate the ratio of the methyl esters of L-valine/L-isoleucine

**Table 68.**
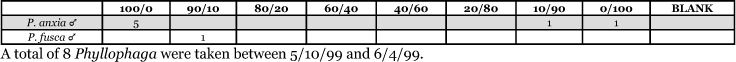
New York, Saratoga Springs 1999 Blends indicate the ratio of the methyl esters of L-valine/L-isoleucine

**Table 69.**
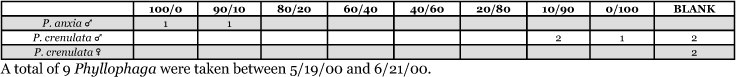
New York, Saratoga Springs 2000 Blends indicate the ratio of the methyl esters of L-valine/L-isoleucine

**Table 70.**
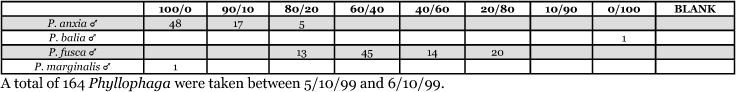
New York, Warwick 1999 Blends indicate the ratio of the methyl esters of L-valine/L-isoleucine

**Table 71.**
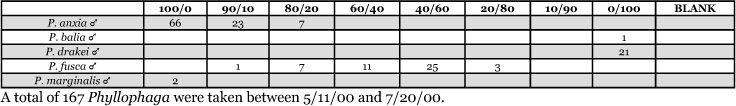
New York, Warwick 2000 Blends indicate the ratio of the methyl esters of L-valine/L-isoleucine

**Table 72.**
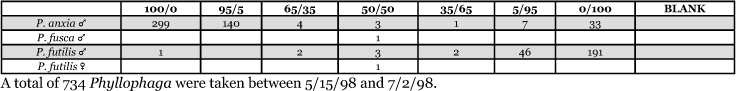
New York, Waterloo #1 1998 Blends indicate the ratio of the methyl esters of L-valine/L-isoleucine

**Table 73.**
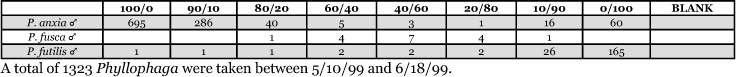
New York, Waterloo #1 1999 Blends indicate the ratio of the methyl esters of L-valine/L-isoleucine

**Table 74.**
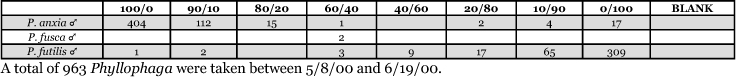
New York, Waterloo #1 2000 Blends indicate the ratio of the methyl esters of L-valine/L-isoleucine

**Table 75.**
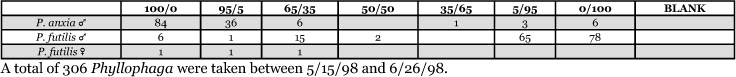
New York, Waterloo #2 1998 Blends indicate the ratio of the methyl esters of L-valine/L-isoleucine

**Table 76.**
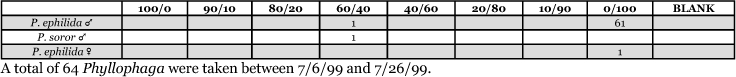
North Carolina, Raleigh #1 1999 Blends indicate the ratio of the methyl esters of L-valine/L-isoleucine

**Table 77.**
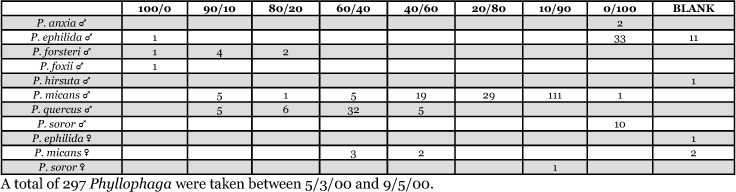
North Carolina, Raleigh #2 2000 Blends indicate the ratio of the methyl esters of L-valine/L-isoleucine

**Table 78.**
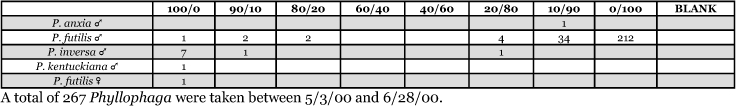
Ohio, Columbus 2000 Blends indicate the ratio of the methyl esters of L-valine/L-isoleucine

**Table 79.**
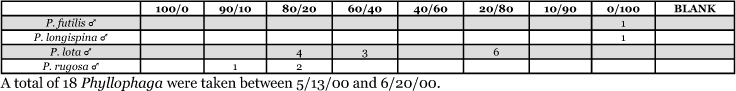
Ohio, Wooster 2000 Blends indicate the ratio of the methyl esters of L-valine/L-isoleucine

**Table 80.**
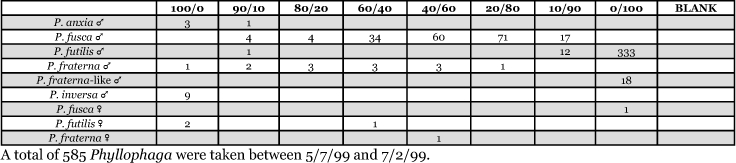
Pennsylvania, State College 1999 Blends indicate the ratio of the methyl esters of L-valine/L-isoleucine

**Table 81.**
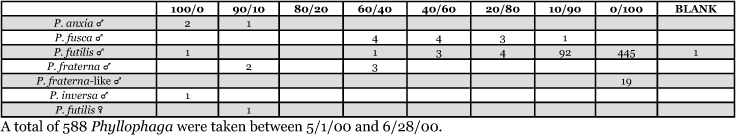
Pennsylvania, State College 2000 Blends indicate the ratio of the methyl esters of L-valine/L-isoleucine

**Table 82.**
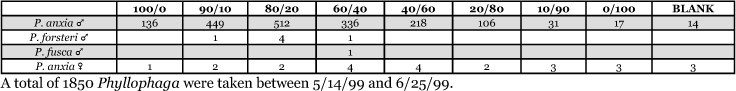
Rhode Island, Kingston 1999 Blends indicate the ratio of the methyl esters of L-valine/L-isoleucine

**Table 83.**
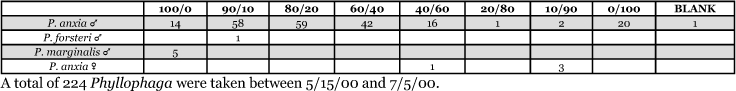
Rhode Island, Kingston 2000 Blends indicate the ratio of the methyl esters of L-valine/L-isoleucine

**Table 84.**
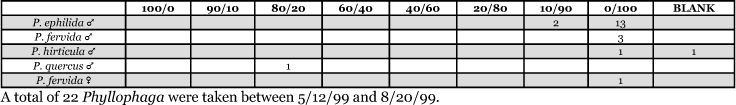
South Carolina, Anderson County 1999 Blends indicate the ratio of the methyl esters of L-valine/L-isoleucine

**Table 85.**
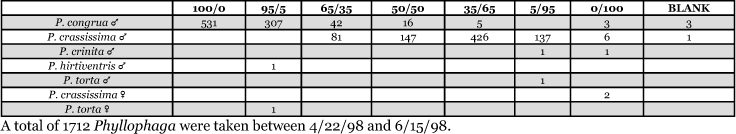
Texas, Dallas #1 1998 Blends indicate the ratio of the methyl esters of L-valine/L-isoleucine

**Table 86.**
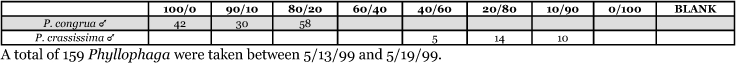
Texas, Dallas #1 1999 Blends indicate the ratio of the methyl esters of L-valine/L-isoleucine

**Table 87.**
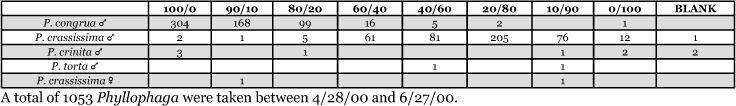
Texas, Dallas #1 2000 Blends indicate the ratio of the methyl esters of L-valine/L-isoleucine

**Table 88.**
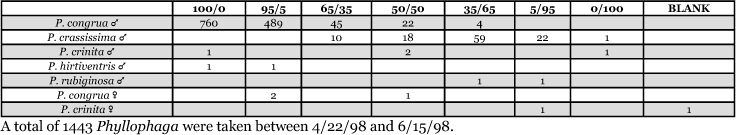
Texas, Dallas #2 1998 Blends indicate the ratio of the methyl esters of L-valine/L-isoleucine

**Table 89.**
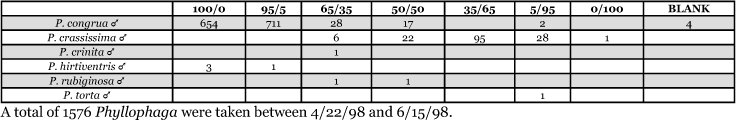
Texas, Dallas #3 1998 Blends indicate the ratio of the methyl esters of L-valine/L-isoleucine

**Table 90.**
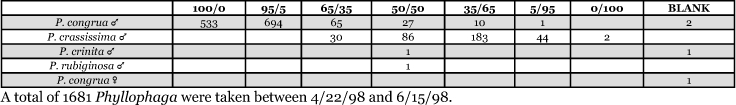
Texas, Dallas #4 1998 Blends indicate the ratio of the methyl esters of L-valine/L-isoleucine

**Table 91.**
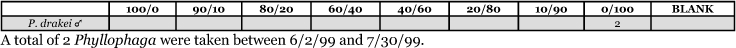
Utah, Salt Lake City 1999 Blends indicate the ratio of the methyl esters of L-valine/L-isoleucine

**Table 92.**
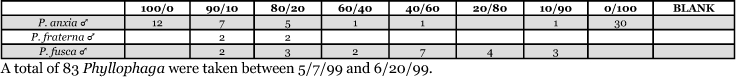
Vermont, Burlington 1999 Blends indicate the ratio of the methyl esters of L-valine/L-isoleucine

**Table 93.**
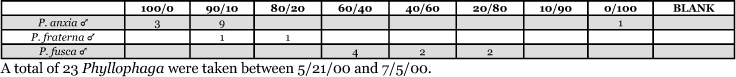
Vermont, Burlington 2000 Blends indicate the ratio of the methyl esters of L-valine/L-isoleucine

**Table 94.**
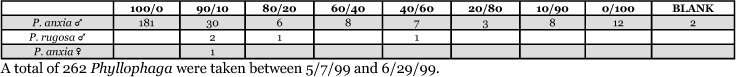
Wisconsin, Babcock 1999 Blends indicate the ratio of the methyl esters of L-valine/L-isoleucine

**Table 95.**
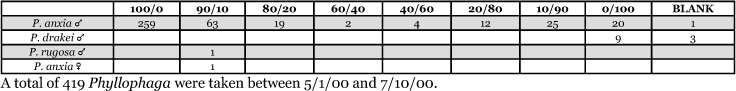
Wisconsin, Babcock 2000 Blends indicate the ratio of the methyl esters of L-valine/L-isoleucine

**Table 96.**
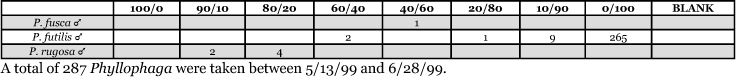
Wisconsin, Verona 1999 Blends indicate the ratio of the methyl esters of L-valine/L-isoleucine

**Table 97.**
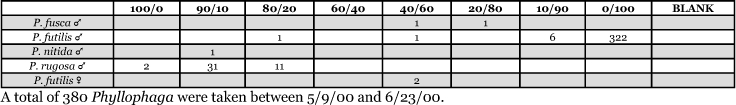
Wisconsin, Verona 2000 Blends indicate the ratio of the methyl esters of L-valine/L-isoleucine

**Table 98a:**
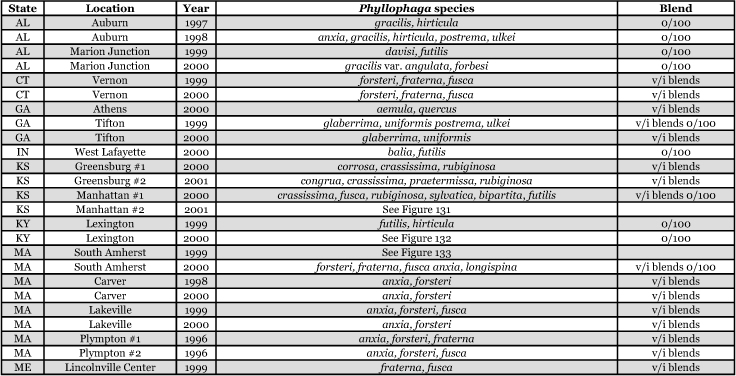
Synchronic species captured by the same or nearby sex attractant blends, by state.

**Table 98b:**
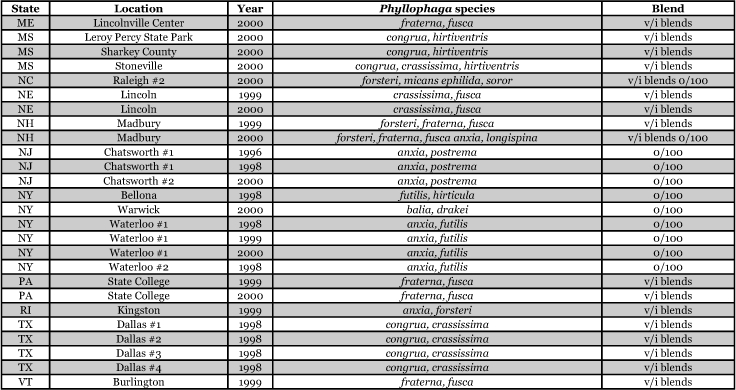
Synchronic species captured by the same or nearby sex attractant blends, by state.

## Summary and Conclusions

This study demonstrates the extensive use of the methyl esters of L-valine and L-isoleucine in the mate recognition systems of a widely distributed and speciose taxon. Since each trapping site is a snapshot of activity over a very restricted area, it is compelling that nearly 40% of the Phyllophaga (sensu stricto) species in America north of Mexico were captured, despite all the possible locations that were not trapped.

Consistency in male response among geographically separated populations of conspecifics was demonstrated in numerous examples. This was useful to document in itself because it provided information concerning sex attractant use in a taxon that had never before been investigated in this fashion. However, this information also served, perhaps more importantly, to contrast and highlight the unusual geographic variation in male response to sex attractants between various populations of P. anxia and in a more minor way, P. fraterna.

This study also documents the interspecific interactions between Phyllophaga species, through both literature citations and capture results. These interactions may be as benign as attraction to congeneric females (Eberhard 1993), with presumably little or no accompanying loss of fitness, or may involve copulation and perhaps exchange of genetic material (Fattig 1944), resulting in a more expensive or even fatal conclusion. Interactions between species are of great interest because of their connection to both species concepts and hybridization studies (Arnold 1997, Claridge et al. 1997, Rand and Harrison 1989).

Since this trapping study was terminated in 2001, additional research on sex pheromones of the Phyllophaga has been accomplished that extends the understanding of mate finding in this large genus. This research will have bearing on the phylogenetic relationships of this group as well.

The sex pheromone of Phyllophaga crinita was identified as methyl 2-(methylthio)benzoate (Robbins et al. 2003). Interestingly, Coca-Abia (2002) has recently resurrected the genus Trichesthes and removed P. crinita as well as a number of other species from the Phyllophaga (sensu stricto) to Trichesthes. Among the species moved to Trichesthes were P. lenis and P. tristis, two species that have also been captured in large numbers using methyl 2-(methylthio)benzoate (P.S. Robbins, unpublished data).

The sex pheromone of Phyllophaga (Tostegoptera) lanceolata was identified as L-leucine methyl ester (Nojima et al. 2003). Tostegoptera is a small sub-genus of the Phyllophaga consisting of only two species, Phyllophaga (Tostegoptera) lanceolata and Phyllophaga (Tostegoptera) squamipilosa. Both species have been captured using L-leucine methyl ester and field tests have demonstrated that L-valine and L-isoleucine methyl esters function as antagonists to Phyllophaga (Tostegoptera) lanceolata males.

## Footnotes

^1^ see Figure 99, P. inversa; Figure 102, P. latifrons; Figure 106, P. marginalis; Figure 126, P. vehemens

^2^ see Figure 68, P. balia; Figure 69, P. bipartita; Figure 74, P. crenulata; Figures 79a and 79b, P. drakei; Figure 82, P. forbesi; Figures 88a, 88b, 88c, and 88d, P. futilis; Figure 92, P. gracilis; Figure 94, P. hirsuta; Figure 95, P. hirticula; Figure 103, P. longispina; Figure 113, P. postrema; Figure 124, P. ulkei

^3^ see Figure 64, P. aemula; Figure 65, P. affabilis; Figure 72, P. corrosa; Figures 73a and 73b, P. crassissima; Figures 83a, 83b, and 83c, P. forsteri; Figures 85a and 85b, P. fraterna; Figures 87a, 87b, 87c, and 87d, P. fusca; Figure 90, P. glaberrima; Figure 91, P. glabricula; Figure 104, P. lota; Figure 107, P. mariana; Figure 108, P. micans; Figure 112, P. perlonga; Figure 114, P. praetermissa; Figure 116, P. quercus; Figure 117, P. rubiginosa; Figures 118a and 118b, P. rugosa; Figure 121, P. sylvatica; Figure 123, P. torta; Figure 125, P. uniformis

^4^ see Figures 73a and 73b, P. crassissima; Figures 87a, 87b, 87c, and 87d, P. fusca; Figure 117, P. rubiginosa

^5^ see Figure 64, P. aemula; Figure 65, P. affabilis; Figures 83a, 83b, and 83c, P. forsteri; Figures 85a and 85b, P. fraterna; Figures 118a and 118b, P. rugosa; Figure 125, P. uniformis

^6^ see Figure 64, P. aemula; Figure 65, P. affabilis; Figure 69, P. bipartita; Figure 72, P. corrosa; Figure 91, P. glabricula; Figure 107, P. mariana; and Figure 125, P. uniformis

^7^ see Figures 11, 73a and 73b, P. crassissima; Figures 12 and 74, P. crenulata; Figures 17, 79a and 79b, P. drakei; Figures 20 and 82, P. forbesi; Figures 21, 83a, 83b, and 83c, P. forsteri; Figures 23, 85a and 85b, P. fraterna; Figures 24, 87a, 87b, 87c, and 87d, P. fusca; Figures 25, 88a, 88b, 88c, and 88d, P. futilis; Figures 27 and 90, P. glaberrima; Figures 29 and 92, P. gracilis; Figures 31 and 94, P. hirsuta; Figures 32 and 95, P. hirticula; Figures 33 and 96, P. hirtiventris; Figures 36 and 99, P. inversa; Figures 40 and 103, P. longispina; Figures 41 and 104, P. lota; Figures 43 and 106, P. marginalis; Figures 45 and 108, P. micans; Figures 47 and 110, P. nitida; Figures 49 and 112, P. perlonga; Figures 50 and 113, P. postrema; Figures 51 and 114, P. praetermissa; Figures 53 and 116, P. quercus; Figures 54 and 117, P. rubiginosa; Figures 55, 118a and 118b, P. rugosa; Figures 58 and 121, P. sylvatica; Figures 60 and 123, P. torta; Figures 61 and 124, P. ulkei; and Figures 63 and 126, P. vehemens
